# A pan-cancer multi-omic *SuperLearner* for regulated cell death survival topologies

**DOI:** 10.3389/frai.2026.1895164

**Published:** 2026-08-20

**Authors:** Emanuell Rodrigues de Souza, Higor Almeida Cordeiro Nogueira, Victor dos Santos Lopes, Enrique Medina-Acosta

**Affiliations:** Laboratório de Biotecnologia, Centro de Biociências e Biotecnologia, Universidade Estadual do Norte Fluminense, Campos dos Goytacazes, Brazil

**Keywords:** explainable AI, multi-omics, non-proportional hazards, pan-cancer, precision oncology, regulated cell death, *SuperLearner*

## Abstract

**Introduction:**

Regulated cell death (RCD) pathways influence tumor progression and immune modulation. We previously constructed a signature database mapping 25 RCD forms across seven multi-omic layers and 33 tumor types (CancerRCDShiny). Despite their ability to identify risk populations, translating these signatures into personalized clinical workflows requires a shift from cohort stratification to individualized risk mapping by modeling patient risk (survival topologies) to capture the non-linear dynamics of RCD signatures.

**Methods:**

We engineered a pan-cancer multi-omic *SuperLearner* pipeline across 33 cancer types. Phase I performed zero-leakage harmonization and groupwise imputation to prevent cross-cohort amalgamation. Phase II deployed Elastic Net-regularized Cox regression as a CANARY diagnostic to map proportional hazards failures. Strata with a 35% missingness barrier entered Phase III, deploying a Quadripartite ensemble: *Random Survival Forests*, *XGBoost*, Survival-*Boruta*, and Multi-Task Logistic Regression, fused within an Elastic Net Multi-View Meta-Learner (*MVL*), with *post-hoc TreeSHAP* and *LIME* interpretability.

**Results:**

The CANARY diagnostic demonstrated the structural invalidity of pan-cancer geometric proportional hazards. Across 96 admissible strata, Phase III executed algorithmic displacement: continuous multi-omic topologies suppressed static genomic mutations and copy number variations (85.7% vs. 0.0% apex retention). The MVL stabilized predictions against extreme variance; *LIME* surrogate validations (*R*^2^ < 0.10) confirmed the systematic failure of linear interpretative proxies. N-dimensional *TreeSHAP* interaction mapping exposed synergistic and antagonistic rescue trajectories defining individualized Survival Topologies, which were invisible to additive models. The architecture was deployed as CancerRCDPredictor, a digital molecular tumor board with integrated LLM capabilities. The *MVL SuperLearner* achieved a median C-index of 0.749 (IQR: 0.722–0.836) across 96 modelable strata, with 95% bootstrap confidence intervals confirming precision (median width: 0.052) and permutation significance in 93.8% of strata (*p* < 0.001). External CPTAC validation across ten cancer types demonstrated significant cross-cohort generalizability in clear cell renal carcinoma (KIRC; C-index 0.675, *p* = 0.017) and modest performance across the remaining adequately powered cancers (median 0.582), underscoring the need for larger multi-institutional validation cohorts.

**Conclusion:**

This pan-cancer multi-omic *SuperLearner* bypasses linear topological failures, advancing beyond generalized stratification to establish a deterministically mapped architecture for predicting RCD-related survival topologies. Through the CancerRCDPredictor interface, multi-omic insights translate into individualized survival topology exploration, providing a foundation for future precision oncology validation.

## 1 Introduction

Regulated cell death (RCD) plays an essential role in tissue homeostasis and cancer treatment response (Galluzzi et al., 2018). Various forms of RCD, including apoptosis, necroptosis, and ferroptosis, distinctly influence tumor progression, immune microenvironment modulation, and therapeutic outcomes. This diverse set of mechanisms provides a unique opportunity to unravel interactions between tumor cells and their microenvironment, particularly within precision oncology frameworks. However, the inherent complexity and interdependence of these processes have historically posed challenges for comprehensive functional characterization and clinical application (Tang et al., 2019).

In a recent comprehensive analysis (Rodrigues de Souza et al., 2025), we constructed an integrated prognostic model that systematically correlated 25 distinct forms of RCD with extensive multi-omic data, including mRNA, miRNA, transcript isoforms, DNA methylation, copy number variation (CNV), mutations, and protein expression, across 33 cancer types from the TCGA study (Goldman et al., 2020; Wang et al., 2022; Li et al., 2024) (see Supplementary Table S1 for specific cancer type abbreviations and full lineage descriptions). In that foundational work, employing a terminological association-based gene selection method, we identified a baseline library of 44,641 multi-omic signatures significantly correlated with various clinical outcomes such as overall survival, mutational burden, and immune infiltration profiles. This model allowed us to distinguish clinically relevant molecular signatures, effectively associating them with immunologically active (“hot”) and immune-suppressed (“cold”) deconvoluted immune cell and tumor microenvironment phenotypes. This classification provides critical insights for selecting appropriate immune-based therapeutic strategies tailored to individual patient profiles.

To facilitate practical clinical applications, we also developed a standardized alphanumeric nomenclature system categorizing multi-omic signatures by biological function, specific RCD mechanisms, key clinical variables, and survival metrics (Rodrigues de Souza et al., 2025). See Supplementary Note 1 for a detailed methodological framework of the 11-component signature identifier. This approach enhances interpretability, accelerates biomarker identification, and supports the seamless integration of these signatures into clinical workflows, promoting their practical use in precision medicine.

Our prior studies also highlighted the critical importance of transcript-level specificity (Rodrigues de Souza et al., 2025). For instance, *MAPK10* demonstrated significant clinical correlations predominantly through specific transcript isoforms, emphasizing the complexity and importance of alternative splicing and transcriptional control mechanisms in cancer biology. Conversely, genes such as *COL1A1* and *UMOD* exhibited consistent phenotypic correlations across isoforms, suggesting coordinated regulatory mechanisms at the gene level linked to tumor stemness and aggressiveness. We identified 879 clinically meaningful multi-omic signatures that included known immunotherapy targets under clinical investigation, reinforcing their immediate translational relevance. To further enhance the practical exploration and visualization of these comprehensive datasets, we developed CancerRCDShiny,^1^ an interactive analysis tool accessible to the broader research community.

Despite the robust prognostic insights generated by this foundational multi-omic catalog, translating these findings into routine precision oncology workflows requires a shift from descriptive stratification toward precise, individual-level prediction. Prognostic models inherently identify generalized biological risk groups, but they lack the algorithmic complexity necessary to deliver exact patient survival predictions. Achieving this transition requires overcoming substantial multidimensional challenges, including extreme data sparsity, missingness, and structural hazards that routinely cause traditional unregularized models to fail when faced with high-dimensional RCD omic signatures.

To bridge the gap between descriptive prognosis and computational prediction, the present study establishes a pan-cancer Audit-Compliant *SuperLearner*, a formal mathematical architecture for Multi-View Meta-Learning ensemble integration (van der Laan et al., 2007), engineered specifically for rigorous predictive benchmarking of patient survival based on RCD omic signatures.

Rather than applying predictive algorithms blindly, we deploy a strictly deterministic, three-phased architecture. Phase I and Phase II act as formal execution barriers to prevent cross-layer biological data leakage and to mathematically diagnose the geometric survival topologies of 33 distinct tumor types. Only mathematically verified strata advance to Phase III, where an advanced non-linear supervised Quadripartite ML ensemble, combining Random Survival Forests (*RSF*) (Ishwaran et al., 2008), Extreme Gradient Boosting (*XGBoost*) (Chen and Guestrin, 2016), Survival-*Boruta* (Kursa and Rudnicki, 2010), and Multi-Task Logistic Regression (*MTLR*) (Yu et al., 2011), operates concurrently with strict *post-hoc* interpretability algorithms: *SHAPley* Additive exPlanations (*SHAP*) (Lundberg and Lee, 2017) and Local Interpretable Model-agnostic Explanations (*LIME*) (Ribeiro et al., 2016). Ultimately, by resolving the high-dimensional sparsity of the prior multi-omic catalog, this framework successfully shifts the biological logic of the 25 distinct forms of RCD into an actionable, fully transparent predictive architecture.

This transition from identifying prognostic factors to developing actionable predictive models represents a significant methodological advancement essential for personalized medicine. This approach holds great promise for improving patient stratification, clinical decision-making, and therapeutic efficacy, substantially enhancing patient management in oncology.

## 2 Materials and methods

### 2.1 Overview of the analytical pipeline

To execute robust patient survival prediction via RCD omic signatures, the analysis is organized as a pan-cancer, audit-compliant *SuperLearner* pipeline. Designed to separate data transformation, eligibility gating, and inferential modeling into distinct operational stages, this framework prevents cross-layer biological leakage and prohibits outcome-driven adaptation.

**Figure 1:**
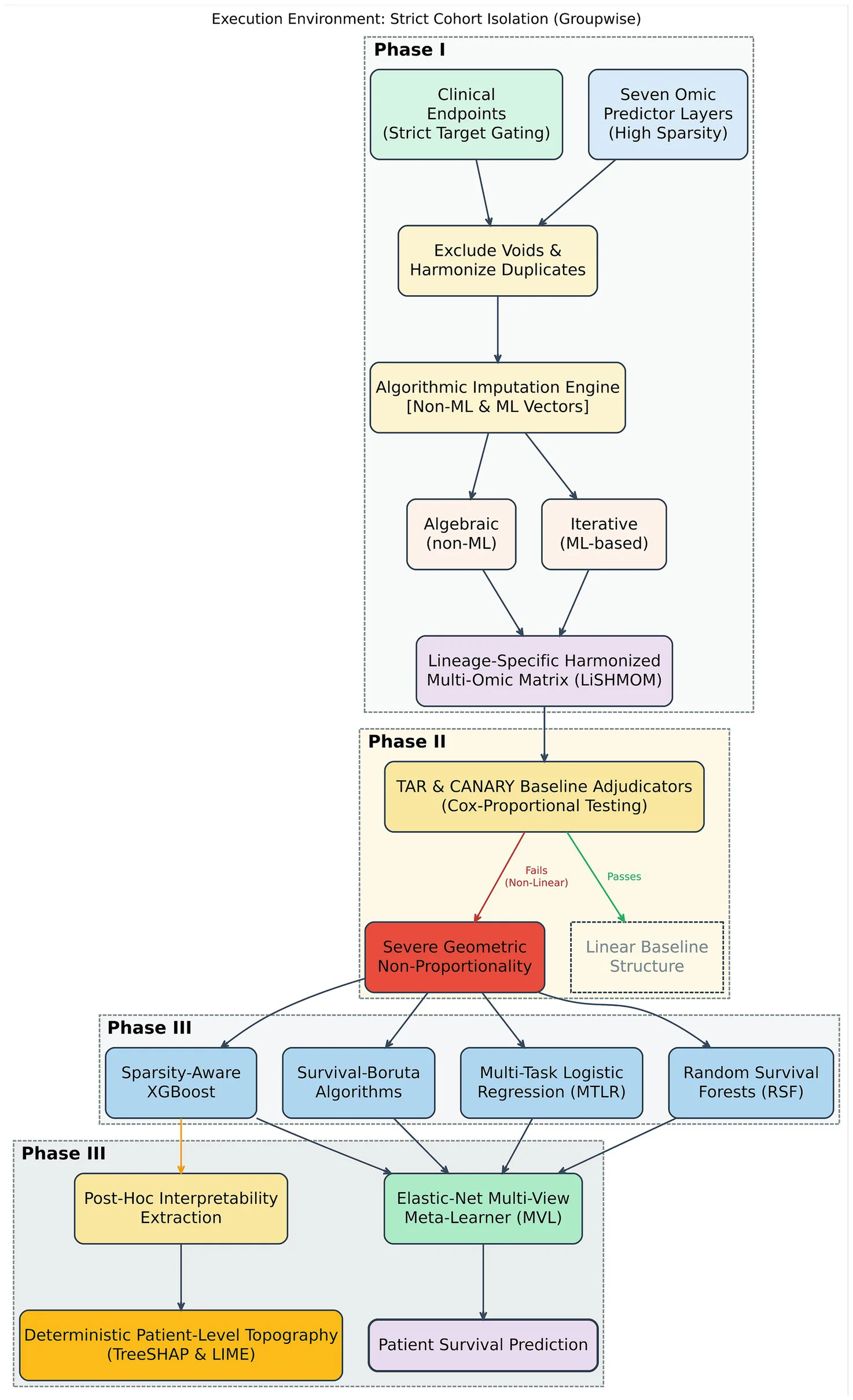
Analytical pipeline architecture: strict cohort isolation and *SuperLearner* execution strategies. Phase I: Raw multi-omic inputs (seven highly sparse omic predictor layers) and strictly gated clinical endpoints undergo a domain-specific harmonization process. All computations operate under a mathematically enforced zero-leakage policy; variables are processed within strictly isolated, cancer-specific groupwise layers. Missing feature spaces are addressed using a dual Algorithmic Imputation Engine, which employs both non-ML algebraic techniques and ML-based iterative solutions to yield standardized, robust Lineage-Specific Harmonized multi-omic Matrices (LiSHMOM) for each pan-cancer lineage. Phase II: The CANARY Baseline Adjudicator rigorously tests the resulting matrices for Cox proportional topological baseline failures. Phase III: Features exhibiting severe geometric non-proportionality are routed into a sophisticated Quadripartite ML Ensemble. This array leverages four discrete, non-linear algorithms: *RSF*, Sparsity-Aware *XGBoost*, Survival-*Boruta* parameters, and *MTLR*. Finally, the predictive vectors from these individual base-learners are systematically synthesized via the *MVL*. This definitive *SuperLearner* integration mitigates individual algorithmic bias, establishing patient survival predictions while utilizing *post-hoc* Interpretability Extraction (*TreeSHAP* and *LIME*) to yield highly transparent, deterministic patient-level topographies for downstream translational exploration.

Phase I generates a library of candidate preprocessing regimes using standard statistical transformations within isolated cancer cohorts. Phase II applies deterministic eligibility and feasibility gates, implemented as Transformation Admissibility Routing (TAR) and a CANARY model, to certify which of these clinical-omic regimes are structurally sound. Only mathematically verified cohorts pass to Phase III, which serves as the exclusive locus for predictive inference. Within Phase III, an advanced non-linear Quadripartite ML Ensemble is derived solely from TAR-admissible regimes, ensuring that any resulting patient survival prediction reflects intrinsic biological structure rather than hidden optimization. A schematic overview of this audit-compliant pipeline, defining phase boundaries and execution gates, is provided in Figure 1.

All analyses in Phases I-II were executed under a fixed set of constitutional contracts defining admissible operations on cohorts, predictors, and execution units. These contracts enforce strict groupwise analysis by cancer type and survival endpoint, prohibit predictor-driven sample reduction, and require explicit, auditable justification for any predictor exclusion. A full specification of these contracts and their audit requirements is provided in Supplementary Note 2.

### 2.2 Phase I: programmatic retrieval, harmonization, and algorithmic reconstruction of multi-omic and clinical data

The primary function of Phase I is the programmatic harmonization and deterministic reconstruction of high-dimensional prognostic matrices. Crucially, Phase I operates strictly as an outcome-independent data processing boundary enforced by rigorous, multi-layered missingness gates designed to prevent the indiscriminate fabrication of synthetic data.

For clinical survival metrics, we enforced an absolute prohibition on the imputation of survival events (OS, DSS, DFI, PFI) to prevent label leakage and optimism bias. Conversely, the imputation of survival times was permitted but governed by three stringent clinical constraints: (1) a variable-level missingness threshold (≤35%), (2) a type-pair gate requiring adequate intra-cohort event prevalence (0.05–0.95), and (3) a strict row-wise constraint mandating that the event status and foundational clinical anchors (e.g., diagnosis year) be observed.

For multi-omic predictors (both binary/categorical and continuous), imputation was strictly limited by the same ≤35% intra-cohort missingness barrier. To maintain the structural integrity of the dataframe without extensive synthetic data fabrication, omic variables exceeding this missingness threshold were deliberately preserved; their missing values were carried forward unchanged to downstream machine learning evaluators. Furthermore, for continuous subsets where extreme sparsity triggered dimensionality failures in multivariate algorithms (which mathematically require ≥2 features), we implemented a deterministic, non-informative dummy-variable injection protocol to satisfy algorithmic constraints without compromising biological integrity or resorting to cross-cohort data pooling.

The 35% threshold was identified through a formal sensitivity analysis across thresholds of 20, 30, 35, 40, and 50% missingness. This analysis revealed a stable plateau at 30–50% where patient and variable retention remained essentially invariant. At 20%, over 2,200 patients with partial but genuine multi-omic data would be unnecessarily excluded; at 50%, near-empty patient vectors would be included. The 35% value was selected as the midpoint of this plateau, conservatively excluding only patients with entirely absent multi-omic profiling (97.9% of excluded patients had 100% missing omic data). The full analysis is documented in Supplementary Note 3.

By passing through these criteria, eligible molecular values were then inferred in a layer-specific, self-reconstruction mode using a comprehensive suite of algorithmic strategies. This suite ranged from deterministic methods (mean, median, mode) and Bernoulli-based probabilistic sampling for binary features (preserving sample-level heterogeneity) to advanced multivariate approaches, including k-nearest neighbors (*KNN*) (Cover and Hart, 1967), Multiple Imputation by Chained Equations (*MICE*) (Buuren and Groothuis-Oudshoorn, 2011), Iterative Rank-Restricted Soft Singular Value Decomposition (*softImpute iSVD*) (Hastie et al., 2015), and non-parametric architectures like *missForest* (Stekhoven and Buhlmann, 2012), light gradient boosting machine (*LightGBM*) (Ke et al., 2017), and *XGBoost* (Chen and Guestrin, 2016). Throughout this entire process, survival endpoints remained fully insulated from the predictor structure, serving exclusively as downstream audit sentinels.

To operationalize this rigorous architectural framework, molecular and clinical data were programmatically retrieved from TCGA using the UCSCXenaShiny resource (Wang et al., 2022; Li et al., 2024). To structure this high-dimensional encoding, we established the unified baseline decoder (Supplementary Dataset S1). Derived from an initial baseline universe of 44,641 pan-cancer multi-omic signatures from our previous work (Rodrigues de Souza et al., 2025), this master decoder was meticulously filtered to isolate features demonstrating robust clinical relevance. Specifically, signatures retained in Supplementary Dataset S1 were required to achieve statistical significance in at least one univariate survival metric (log-rank and Cox proportional hazards indicating definitive risk or protection) and demonstrate significant associations with deconvoluted immune infiltrates (classified as “Hot,” “Cold,” “Variable,” or “NS”) and tumor microenvironment profiles (“anti-tumoral,” “pro-tumoral,” “dual,” or “NS”). Through this rigorous selection, we curated a highly stable catalog of 14,907 distinct multi-omic prognostic nomenclatures, which collectively encompass 17,875 unique biological target elements (spanning gene symbols, transcript isoforms, miRNAs, and proteins). By formally formatting these targets under the variables Signature, Nomenclature, CTAB (cancer type abbreviation), and Omic_feature, and mapping each abstract alphanumeric token directly to its constituent genetic elements and functional RCD mechanics, Supplementary Dataset S1 served as the invisible architectural blueprint dictating the extraction and formatting of the multi-omic features subsequently compiled into the clinical modeling matrix. Importantly, this filtering was conducted exclusively via univariate population-level tests and did not involve any machine learning model training. Dataset S1 therefore represents a population-level candidate screening, not a model-optimized feature selection. All predictive model training occurs downstream in Phase III, within strictly isolated cancer–endpoint strata, and no model-internal feature selection based on survival outcomes was performed at any pipeline phase.

### 2.3 External validation strategy

The Clinical Proteomic Tumor Analysis Consortium (CPTAC) cohort (Edwards et al., 2015) was selected as the external validation resource following a systematic evaluation of publicly available multi-omic cancer cohorts. No other consortium currently provides simultaneous coverage of the seven distinct omic layers required by our predictive framework (protein abundance, somatic mutation, CNV, DNA methylation, mRNA expression, miRNA expression, and transcript isoform abundance) across multiple cancer types. The Pan-Cancer Analysis of Whole Genomes (PCAWG) consortium provides only two to three omic layers (mutation and CNV, with partial mRNA coverage), and its multi-cancer projects are predominantly TCGA-contributed, precluding independent external validation. The Molecular Taxonomy of Breast Cancer International Consortium (METABRIC) is restricted to a single cancer type (breast) with four layers but lacks protein, miRNA, and isoform data. The ICGC-25 K and ICGC-ARGO initiatives provide variable omic coverage (one to four layers) but lack proteomic characterization across all evaluated cancer types and remain incomplete for several tumor cohorts. CPTAC uniquely offers harmonized, mass-spectrometry-based proteogenomic profiling across ten cancer types with complete seven-layer omic coverage, making it the only currently available resource suitable for independent multi-omic external validation of a pan-cancer *SuperLearner* architecture.

For independent external validation, we generated a TCGA-compatible CPTAC dataframe using a fully independent pan-cancer preprocessing pipeline. Raw CPTAC molecular profiles were obtained from ten tumor cohorts: breast (BRCA, *N* = 121), clear cell renal (CCRCC, *N* = 103), colorectal (COAD, *N* = 107), glioblastoma (GBM, *N* = 99), head and neck squamous (HNSCC, *N* = 108), lung squamous (LSCC, *N* = 108), lung adenocarcinoma (LUAD, *N* = 110), ovarian (OV, *N* = 83), pancreatic ductal (PDAC, *N* = 105), and uterine corpus endometrial (UCEC, *N* = 95), totaling 1,039 tumor patients. Each cohort was uniformly processed across the same seven omic layers used in the discovery framework: protein abundance, somatic mutation, CNV, transcript isoform expression, mRNA expression, DNA methylation, and miRNA expression. Transcript-level FPKM matrices were converted to TPM, and mRNA TPM matrices were harmonized to log2(TPM + 0.001). All omic tables were then combined into patient-by-feature matrices, and Ensembl gene identifiers were mapped to HGNC symbols where possible (99.7% of protein, 99.3% of mutation, and 97.9% of methylation features resolved). CPTAC tumor labels were deterministically mapped to their TCGA equivalents (CCRCC to KIRC, HNSCC to HNSC, LSCC to LUSC, PDAC to PAAD) to ensure compatibility with the signature definitions and the df005 reference schema. Each of the 3,672 RCD-associated signatures, ranging from 40 for OV to 688 for KIRC, was reconstructed by resolving its component genes through direct feature matching, gene symbol-to-Ensembl conversion, or alias-based lookup. Multi-gene signatures were computed as arithmetic row sums only when all required components were available, with unresolved signatures retained in audit logs rather than artificially imputed. Signature values were assigned exclusively within the tumor type for which each signature was originally defined, preventing cross-cancer leakage. Only CPTAC patients with tumors were subsequently merged with their available clinical data, which included overall survival (OS.time and OS event indicator; available for 92.3 and 96.8% of rows, respectively). TCGA-standard columns from the df005 reference (including DSS, DFI, PFI, PFS, race, and vital status) were incorporated for schema compatibility but remain unpopulated for the CPTAC cohort, as no patient overlap exists between the two datasets. Clinical variables were renamed to match the df005 TCGA nomenclature, and the finalized matrix was serialized as CPTAC_validation.rds. Arithmetic reconstruction audits confirmed that all multi-gene signature values were correctly generated from their constituent features, and gene-coverage audits verified that no molecular features were lost during table integration prior to downstream independent validation.

Predictive performance on the CPTAC external cohort was assessed by independently applying all five predictive components of each frozen TCGA Phase III model bundle (XGBoost, RSF, MTLR, Boruta-RSF, and the MVL *SuperLearner* ensemble) to the imputed CPTAC matrices. No model was retrained, re-tuned, or re-selected. XGBoost and RSF generated risk scores through their native prediction interfaces. MTLR risk scores were reconstructed from the stored model coefficients and training feature terms, as the serialized model objects do not support direct prediction on new data. Boruta-RSF predictions were obtained by applying the TCGA-trained random survival forest to the Boruta-confirmed features present in the CPTAC matrix. The MVL ensemble was synthesized by scaling the four base-learner risk columns to the TCGA-derived meta-matrix and applying the stored Elastic Net combination weights. A pre-flight verification script confirmed the integrity and alignment of all validation assets.

Because the CPTAC omic predictor space is a strict subset of the TCGA feature list (i.e., every CPTAC feature exists within the TCGA matrices, but not all TCGA features are present in CPTAC), the models encounter structurally missing columns during prediction. This missingness pattern is unidirectional: no CPTAC feature exists outside the TCGA predictor space, so the models never encounter novel or out-of-distribution variables. The missing columns are handled natively by each algorithm’s prediction-time architecture: *XGBoost* routes missing variables via default-branch routing, *RSF* and the *Boruta*-proxy *RSF* use internal proximity-based imputation (na.action = “*na.impute*”) computed from the CPTAC data itself rather than from TCGA training statistics, and *MTLR* applies *missForest* imputation prior to linear combination. Using CPTAC-native imputation at prediction time is the methodologically correct choice for external validation: injecting TCGA training statistics would introduce cross-cohort information leakage, whereas on-the-fly imputation from the validation data preserves the independence of the external test.

This design makes the validation conservative by construction. The TCGA-trained models encounter a reduced feature set in CPTAC, which provides a realistic lower-bound assessment of cross-cohort generalizability. No information pathway exists by which the reduced feature set could inflate C-index, AUC, or calibration metrics above their TCGA-derived values. CPTAC performance estimates are therefore lower-bound estimates of the model’s cross-cohort generalizability, reflecting both genuine biological validity and the dimensional constraints inherent to current multi-omic external validation resources.

Clinical, demographic, and survival metadata were programmatically retrieved from the TCGA using the UCSCXenaShiny package (Wang et al., 2022; Li et al., 2024). To construct the foundational dataset, distinct multi-omic expression profiles, encompassing mRNA, miRNA, protein, transcript isoforms, CNVs, DNA methylation, and somatic mutations, were systematically aggregated into a unified, high-dimensional matrix. To ensure structural integrity during assembly, malformed entries were excluded, and the molecular profiles were merged with the clinical survival records via unique sample identifiers. Finally, the resulting matrix underwent strict deduplication and was filtered to remove any instances exhibiting total systemic missingness across all molecular and response features. This finalized, cleaned dataset was subsequently serialized as the foundational baseline to feed the downstream harmonization and imputation architectures.

During this deduplication process, repeated patient entries were systematically isolated and examined. For each duplicated group, clinical survival variables were cross-referenced. Where discrepancies existed, a strict administrative harmonization protocol was applied, replacing fragmented records with values from the most complete row to ensure internal consistency across all binary outcome indicators and survival time variables [Overall Survival (OS), Disease-Specific Survival (DSS), Disease-Free Interval (DFI), and Progression-Free Interval (PFI)]. Crucially, this procedure functioned purely as deterministic harmonization for schema auditing; it did not involve any statistical modeling, probabilistic sampling, or stochastic alteration of survival outcomes. These administratively consolidated blocks were subsequently used to overwrite the original duplications, yielding the definitive dataset.

Prior to advanced imputation, foundational predictors were structurally preprocessed. CNV features (marked by the “0.3” nomenclature suffix) were transformed into explicit categorical states (“Deleted,” “Normal,” or “Duplicated”), while somatic mutation features (denoted by “0.2”) were binarized to indicate wild-type (0) or mutated (1) status. This native observed matrix was designated as the df005 pre-imputation reference baseline (Supplementary Dataset S2). Serving as the fixed architectural anchor against which all downstream imputation variants were evaluated, the harmonized df005 baseline comprises 10,489 pan-cancer samples and 14,657 distinct variables (consisting of 62 clinical/demographic anchors and 14,595 multi-omic features).

### 2.4 Omic token encoding and predictor layer abstraction

The biological definition of multi-gene signatures and their molecular composition have been described in detail in our prior work (Rodrigues de Souza et al., 2025; Almeida Cordeiro Nogueira and Medina-Acosta, 2026; Almeida Cordeiro Nogueira et al., 2026). In the present study, we focus on the computational encoding used to operationalize these predictors for large-scale preprocessing and modeling. Each molecular predictor was encoded using a tokenized nomenclature system in which a numeric suffix embedded in the Nomenclature field identifies its omic layer. Seven layers were represented: protein abundance (0.1), somatic mutation status (0.2), CNV (0.3), microRNA expression (0.4), transcript isoform abundance (0.5), mRNA expression (0.6), and CpG methylation (0.7). This token-based abstraction enabled programmatic parsing of feature type, enforcement of layer-specific preprocessing and imputation rules, and stratified handling of predictors across cancer types. By decoupling biological identity from measurement modality, the pipeline ensured that transformations and missing data strategies were applied consistently within each omic class while preserving cross-layer comparability. The token system further supported deterministic audit logging and downstream admissibility evaluation in Phase II.

### 2.5 Clarification on survival variable handling in Phase I

To prevent label leakage and optimism bias, survival variables were strictly insulated from statistical imputation. Instead, a highly restricted administrative completion protocol was applied exclusively to resolve deterministic schema inconsistencies between event indicators and their paired time-to-event axes. Limited to a negligible fraction of records, these logical alignments did not introduce synthetic biological signals or alter the underlying cohort censoring structure. Consequently, all Phase I datasets containing administratively aligned survival fields were treated strictly as tentative preprocessing variants. Their final admissibility for downstream machine learning was determined by the topological baseline adjudicator in Phase II, where survival endpoints served exclusively as quality-control sentinels. This structural audit ensured that the tightly controlled multi-omic imputation, which was mathematically required to resolve NA values and feed the non-linear Phase III architectures, did not distort the baseline outcome topography.

### 2.6 Distinction between survival harmonization and time completion in Phase I

Survival-related processing in Phase I comprised two conceptually distinct operations. First, deterministic harmonization was performed to reconcile endpoint labels and observed time-to-event values across authoritative clinical inputs and duplicate records, yielding a coherent ground-truth survival schema that was finalized prior to downstream processing. Second, limited administrative completion was applied exclusively to time-to-event variables that were missing at baseline, under strict eligibility and consistency constraints, with policy enforcement restricted to imputed cells only. Survival event indicators were not modified after harmonization, and observed (non-missing) baseline times were never altered. These two operations were intentionally separated to preserve censoring structure and ensure that downstream analyses evaluated preprocessing sensitivity auditing rather than inferential survival estimates.

### 2.7 Missing data imputation

To address the challenge of missing data in high-dimensional pan-cancer datasets, we implemented a modular, fault-tolerant imputation pipeline that sequentially integrates CNVs, mutation status, and continuous molecular features across seven omic layers. Survival endpoints were retained throughout the pipeline exclusively as audit and consistency variables and were not subjected to statistical imputation for inferential purposes. Importantly, survival endpoints were never used as predictors, targets, loss functions, or optimization objectives for the reconstruction of omic features with missing values.

Limited administrative completion of survival outcome indicators (OS, DSS, DFI, PFI) and their corresponding time-to-event variables was performed under strict, deterministic logical consistency rules to resolve internal schema inconsistencies (e.g., alignment between event indicators and time variables) and to support downstream preprocessing sensitivity auditing. These operations were deterministic, auditable, and affected a mathematically negligible fraction of the global dataset, exclusively related to the alignment of time-to-event variables [Overall Survival Time (OS.time) = 0.52%; Disease-Specific Survival Time (DSS.time) = 0.94%; Progression-Free Interval Time (PFI.time) = 0.42%]. The administratively aligned fraction for Disease-Free Interval Time (DFI.time = 9.96%) was proportionately elevated solely due to the expected structural missingness of ‘disease-free’ clinical states in specific cohorts (e.g., glioblastoma, where total resection is clinically implausible). Crucially, the fundamental clinical event indicators (OS, DSS, PFI, DFI) were explicitly locked and mathematically prohibited from alteration, ensuring no synthetic biological signal was introduced into the survivorship topography. Administrative completion was applied per cancer type, yielding three preprocessing variants with administrative completion of survival time fields (df006–df008), used exclusively for preprocessing sensitivity auditing. The DFI.time completion fraction (9.96% of DFI records) is concentrated in cancers where disease-free clinical status is structurally inapplicable (e.g., glioblastoma and acute myeloid leukemia, where surgical resection and subsequent DFI documentation are not clinically meaningful). None of the DFI-modelable CANARY-admitted strata derive from these cohorts, ensuring that DFI performance metrics are unaffected by administrative time completion.

Each of the three administratively completed survival variants was then subjected to CNV imputation using three methods: mode (most frequent value), random sampling, and k-nearest neighbors (*KNN* via VIM::KNN), executed groupwise by tumor type. CNV values were pre-standardized to categorical states (“Normal,” “Duplicated,” “Deleted”). When *KNN* failed because of low sample size or convergence limitations, mode imputation was used as a fallback. This produced nine CNV-imputed datasets (df009–df017).

Mutation features, previously binarized (0 = wild-type, 1 = altered), were imputed in these nine datasets using four strategies: mean, median, mode, and Bernoulli sampling based on empirical alteration frequencies. This resulted in 36 mutation-imputed datasets (df018–df053).

Each mutation-imputed dataset served as input for the imputation of continuous omic features, including protein expression (0.1), miRNA (0.4), transcript isoform abundance (0.5), mRNA expression (0.6), and CpG methylation (0.7). Nine distinct imputation algorithms were applied independently to each dataset: mean, median, random sampling, *KNN*, *MICE*, *missForest*, *XGBoost*, *LightGBM*, and *iSVD*. This final step produced 372 Lineage-Specific Harmonized multi-omic Matrices (LiSHMOM), 324 of which are continuous-layer–imputed datasets organized by method: mean (df054–df089), median (df090–df125), random (df126–df161), *KNN* (df162–df197), *missForest* (df198–df233), *XGBoost* (df234–df269), *LightGBM* (df270–df305), *MICE* (df306–df341), and *iSVD* (df342–df377) (Supplementary Figure S1; Supplementary Table S2).

At each stage of the pipeline, intermediate matrices were systematically version-controlled and dynamically purged from active memory to optimize computational efficiency. A dynamic checkpoint engine automatically resumes the pipeline from the last successfully validated output in the event of hardware failure. Every generated dataset was indexed and registered in a centralized architectural ledger, enabling strict deterministic traceability for downstream modeling. This framework yielded 372 version-controlled preprocessing variants (df006–df377), materialized as indexed data objects, which constitute candidate preprocessing regimes generated in Phase I. These variants were not assumed to be admissible for modeling *a priori*; their admissibility was evaluated exclusively by TAR in Phase II on a cancer- and endpoint-specific basis.

### 2.8 Statistical rationale for layer-specific imputation architectures

The multi-layered imputation strategy described above was explicitly tailored to respect the distinct biological and statistical characteristics of each omic data type. For categorical CNV states (“Normal,” “Duplicated,” “Deleted”), imputation was restricted to domain-appropriate strategies to ensure cytogenetic constraints were not violated. Crucially, a programmatic fallback logic automatically defaulted to mode imputation whenever *KNN* convergence failed due to cohort-specific multicollinearity or low sample sizes.

For binarized somatic mutations, Bernoulli-based probabilistic sampling was specifically integrated alongside standard estimators. The Bernoulli method was critical for mutation layers because it maintains the empirical alteration frequency across tumors, thereby preserving the stochastic representation of both rare and common driver events without introducing fractional artifacts.

Finally, the deployment of nine distinct algorithms across the continuous features (protein abundance, miRNA, mRNA, transcript isoforms, and CpG methylation) was designed to comprehensively cover the spectrum of modern imputation philosophies: parametric estimators (mean, median), local topological similarity (*KNN*), iterative ensemble regression (*missForest*), gradient boosting engines (*XGBoost*, *LightGBM*), and probabilistic chaining (*MICE*, *iSVD*). By deploying this architectural diversity across five highly heterogeneous continuous layers, the pipeline enabled a robust sensitivity audit. This ensured that the predictive Phase III machine learning models could be evaluated against preprocessing strategies capable of handling extreme data sparsity, non-linear dependencies, and massive dimensionality.

### 2.9 Evaluation strategy for imputation performance across output datasets

To ensure the mathematical validity and interpretability of each imputation method, a systematic evaluation framework was implemented to audit the downstream effect of predictor imputation on survival-related signal preservation. At every stage of the pipeline, intermediate matrices were systematically version-controlled and registered in a centralized tracking ledger. This architectural design ensured deterministic mapping from raw inputs to imputed variants, guaranteeing strict fault tolerance and reproducibility throughout the pipeline.

The complete set of 372 preprocessing variants was analyzed using a structured diagnostic module that computed concordance indices (C-index) for each survival endpoint (OS, DSS, DFI, PFI) via Cox proportional hazards models. Crucially, these models were deployed strictly as diagnostic probes of signal preservation rather than as inferential endpoints. For each imputation method, C-index trajectories were compared across endpoints and plotted against the baseline percentage of missingness in the original data. This permitted a rigorous evaluation of algorithmic robustness (stability under high missingness) versus informativeness (preservation of prognostic topological structure).

Furthermore, pairwise correlations between data missingness and diagnostic performance were computed per imputation strategy and endpoint. This structural audit enabled the explicit identification of methods susceptible to artifactually inflated performance (e.g., complete-case baseline distortions) versus algorithms that remained unaffected by missing data proportions. Ultimately, these diagnostics did not dictate direct model selection; rather, they served as strict admissibility gates to qualify matrices for downstream machine learning evaluation.

### 2.10 Phase I imputation constraint and hallucination audit

To explicitly prevent synthetic data hallucination and preserve cohort geometry, the Phase I architecture enforced strict algorithmic gates and downstream topological brakes. Multi-omic parametric imputation was restricted exclusively to predictors exhibiting ≤35% groupwise missingness. Across the pan-cancer matrix, this eligible pool comprised 592,191 global missing cells. However, due to strict secondary algorithmic constraints, such as matrix convergence limitations and multicollinearity brakes integrated within the *MICE* and *iSVD* engines, the pipeline effectively imputed only ~11.8% of these eligible cells. The remaining 523,587 cells were safely carried through the pipeline as untouched residual NA values in the final matrices (e.g., df377). Furthermore, to ensure strict architectural separation between predictor imputation and clinical outcomes, the pipeline enforced a hard structural lock on all survival endpoints. Tracking the 852 baseline NA cells present across the eligible survival time variables in the raw matrix revealed that exactly 852 NA cells remained in the fully imputed variants. This mathematically proves that none of the nine continuous machine learning imputation engines ever breached the clinical survival boundary, definitively ensuring that no inferential survival signal was synthetically generated.

### 2.11 Universal resume engine and execution ensures

Imputation procedures in Phase I were executed using a fault-tolerant, audit-oriented execution engine implemented in R for large-scale, groupwise multi-omic processing. The engine supports multiple imputation strategies spanning parametric, similarity-based, ensemble, boosting, and matrix completion approaches. Imputation was performed independently within cancer-type strata to prevent cross-cohort contamination.

The execution framework enforces deterministic checkpointing, full provenance tracking, and version-controlled materialization of each preprocessing regime as an indexed RDS object registered in a centralized audit table. Intermediate failures are isolated at the variable or method level and do not interrupt global pipeline completion. This design ensures that all preprocessing variants entering Phase II are reproducible, auditable, and free of cross-endpoint or cross-cancer information leakage.

Throughout the manuscript, the term *candidate preprocessing regime* refers to any Phase I variant in the df006–df377 series, whereas *TAR-admissible preprocessing regimes* refer specifically to the subset of these variants classified as Unchanged or Improved by TAR for a given cancer × endpoint stratum. Canonical terminology definitions used throughout Phase I–III analyses are summarized in Supplementary Table S3.

Implementation details, operational audit criteria, and software validation procedures for the Phase I imputation engine are provided in the Supplementary Information.

### 2.12 Phase II: TAR-compliant survival modeling feasibility and execution framework

Phase II introduces supervised survival modeling exclusively as a diagnostic mechanism rather than a predictive exercise. Elastic Net-regularized Cox (*CoxNet*) regression is deployed as a CANARY model to probe the structural admissibility of proportional hazards geometry within each cancer–endpoint–preprocessing stratum, under fixed and auditable constraints. Models are executed with fixed, pre-specified identifiability constraints and deterministic regularization ladders, and are evaluated solely for feasibility properties such as convergence, coefficient stability, and the absence of degenerate risk scores.

No predictive optimization, hyperparameter tuning, feature selection, or model comparison is performed in Phase II. Survival outcomes are used only to test whether a given preprocessing regime supports a coherent hazard representation under auditable constraints. Model failure is interpreted as a diagnostic signal of data geometry (e.g., nonlinearity, time dependence, information insufficiency), rather than a modeling deficiency.

Thus, Phase II uses supervised modeling as a structural audit mechanism, not as a predictive exercise.

Phase II was designed as a strictly executional stage that operationalizes survival modeling under the constraints imposed by TAR, which functions as a pre-modeling validity gate that evaluates whether upstream preprocessing regimes preserve survival-relevant structure for each cancer type and survival endpoint. Each preprocessing regime represents a distinct, auditable combination of clinical harmonization and omic missingness handling generated in Phase I (materialized as indexed datasets df006–df377). Using survival endpoints exclusively as external quality-control probes within the preprocessing admissibility stage, TAR classifies each regime, for each cancer–endpoint pair, as Unchanged, Improved, or Degraded relative to a fixed unimputed reference (df005). Only regimes classified as Unchanged or Improved are admissible for Phase II. TAR is explicitly diagnostic and exclusionary; it does not constitute model selection, performance optimization, feature selection, or cohort harmonization, and TAR decisions are never revised during Phase II. Phase II did not generate or modify preprocessing datasets. Instead, TAR evaluated Phase I candidate preprocessing regimes (df006–df377) and annotated their admissibility on a cancer- and endpoint-specific basis.

Phase II respects TAR admissibility as a hard gate and executes supervised survival model forms as feasibility probes only on TAR-approved preprocessing regimes. All Phase II operations are endpoint-specific: OS, DSS, DFI, and PFI define independent cohorts. Missingness in one endpoint never excludes a sample from the analysis of another endpoint. No global completeness constraints are imposed at any stage; in particular, Phase II prohibits complete-case filtering across predictors or omic layers. The only permissible sample-level exclusion is missing or schema-invalid survival information for the specific endpoint under analysis. Phase II does not modify, tune, or re-evaluate upstream preprocessing strategies and does not select preprocessing regimes based on Phase II outcomes.

All Phase II decisions operate within a single execution unit defined by the tuple (cancer type *c*, survival endpoint *m*, dataset variant *d*, algorithm *a*). No rule, filter, or feasibility decision is permitted to operate outside this unit. This design prevents implicit information sharing across cancers, endpoints, preprocessing regimes, or algorithms.

Survival cohort definition in Phase II is strictly endpoint-scoped. For a given cancer type and endpoint, survival time and event indicators are extracted and validated against a canonical schema: survival time must be finite and non-negative when observed, with time equal to zero explicitly permitted; negative survival times are invalid and trigger hard failure. Event indicators must be binary (0/1) when observed. Samples are retained only if both time and event are observed and schema-valid for the selected endpoint. No predictor-driven row deletion is permitted. Cohort masking is therefore defined exclusively by survival feasibility for the endpoint under analysis, ensuring that cohorts are neither intersected across endpoints nor altered by predictor missingness.

Predictor handling in Phase II is algorithm-aware. Algorithms are categorized according to their tolerance for missing predictor values. Tree-based survival models are permitted to operate directly on matrices containing missing values, whereas linear or finite-matrix models require fully finite numeric predictors.

Residual predictor missingness after TAR-approved preprocessing is resolved exclusively at the machine learning execution stage through a hierarchical safety mechanism. Feature-level removal is applied first to eliminate numerically inadmissible predictors within a local cancer × endpoint × preprocessing regime stratum. Only if missingness persists and the selected algorithm requires a finite design matrix is deterministic, outcome-independent imputation applied as a last resort. Sample-level exclusion due to predictor missingness is explicitly disallowed.

For algorithms requiring finite matrices, residual predictor missingness after TAR-approved preprocessing is handled through a strictly ordered, local repair hierarchy applied only within the execution unit. First, the dataset is used as provided. Second, predictors with excessive missingness are removed at the feature level only; sample-level deletion is prohibited. Third, remaining missing values are deterministically imputed using outcome-independent, model-independent rules computed locally within the endpoint-scoped cohort. All such operations are auditable and logged. Global predictor intersections, stochastic imputation, outcome-aware imputation, and cross-endpoint cohort enforcement are explicitly prohibited.

A central component of Phase II is the use of CoxNet regression as a structural feasibility probe rather than as a universal predictive model. CoxNet operationalizes the CANARY diagnostic under fixed statistical identifiability requirements, including minimum sample size and minimum number of observed events (detailed in Supplementary Note 2), and under a deterministic *μ*-ladder that progressively applies increasingly permissive structural relaxations. These relaxations include variance filtering, elastic-net path constraint adjustments, univariate prescreening with collinearity pruning, and ridge stabilization. At each μ level, model fitting is evaluated solely for structural feasibility: convergence, stability of coefficients, and non-degenerate risk scores. No performance optimization, hyperparameter tuning, or cross-validation-based selection is performed.

To independently corroborate the CANARY diagnostic using a complementary parametric approach, Schoenfeld residual analyses (*cox.zph*) (Grambsch and Therneau, 1994) were performed on standard unpenalized Cox proportional hazards models applied to the same 120 cancer–endpoint LiSHMOM strata under the same gating constraints used during the Phase II CANARY audit. Prior to model fitting, zero-variance predictors were removed, and records with remaining missing values were excluded via complete-case analysis. The global Schoenfeld test was then applied to each converged model; strata for which the high-dimensional predictor space precluded valid Schoenfeld computation were documented as computationally non-evaluable.

Successful feasibility indicates that the stratum geometrically admits a sparse, approximately linear hazard representation consistent with proportional hazards assumptions under auditable constraints, without implying superiority over alternative modeling families. Conversely, systematic *μ*-ladder exhaustion or early data infeasibility is interpreted as concrete evidence of a dense, non-linear, time-varying, or information-deficient survival structure incompatible with sparse proportional hazards modeling. Importantly, these outcomes are treated as intrinsic diagnostic properties of the data rather than modeling failures. The CANARY role of CoxNet therefore provides a principled, model-based structural audit prior to any downstream inference.

Throughout Phase II execution, extensive structural audits are enforced to ensure reproducibility and traceability. These include strict schema validation, row-identity preservation utilizing explicit sample identifiers, alignment checks between predictor matrices and survival vectors, and external-universe audits that verify cancer and endpoint coverage against a reference manifest rather than relying on TAR-filtered inputs. Parallel execution is performed using deterministic, audited multisession workflows, with per-stratum worker provenance permanently recorded to certify actual parallel execution.

The strict output of Phase II is a stratum-resolved comprehensive feasibility log indexed by cancer type, survival endpoint, and preprocessing regime. This log explicitly records survival cohort sizes, event counts, predictor dimensionality before and after feasibility repair, feature-level retention ratios, sample-level retention ratios, *μ*-ladder diagnostics, explicit failure codes, and audit metadata. It functions exclusively as a formal eligibility and classification artifact that captures survival data sufficiency and geometric compatibility; it does not encode model performance, parameter estimates, or inferential results. In the subsequent Results section, these feasibility outcomes are summarized descriptively to document the distribution of identified survival geometry classes.

No parameter estimates, risk scores, model coefficients, or performance metrics generated in Phase II are retained, reported, or reused in any downstream analysis.

### 2.13 Phase III: supervised ML for outcome prediction

Only after preprocessing regimes have passed formal eligibility and feasibility gates does the analysis proceed to Phase III. This phase constitutes the exclusive stage for predictive modeling, in which supervised survival models are applied under strictly controlled, TAR-compliant conditions.

Phase III model-family authorization is determined exclusively by Phase II structural diagnostics. In principle, strata supporting sparse proportional hazards geometry would be eligible for Cox-based inference. However, Phase II feasibility analysis revealed that no evaluable cancer–endpoint stratum satisfied sparse proportional hazards compatibility under the specified auditable constraints. All modelable strata were therefore classified as structurally proportional hazards-incompatible yet modelable, indicating the absence of a sparse, approximately linear hazard structure despite sufficient survival information for downstream inference (see Supplementary Note 2 for mathematical justification).

Accordingly, Phase III is designed to deploy survival modeling families capable of accommodating non-linear, interaction-rich, or time-varying hazard structures, including non-linear ensemble methods and non-proportional hazards architectures. These modeling families are incorporated sequentially within the same Phase III framework, with each algorithm specified, implemented, and evaluated independently under identical structural and audit constraints.

All Phase III analyses are restricted to TAR-admissible preprocessing regimes, and cohort composition remains fixed within each cancer–endpoint stratum. No preprocessing, feature filtering, or cohort modification occurs at this stage. Phase III does not revisit TAR classifications or Phase II feasibility outcomes, and no Phase III model performance metric is permitted to retroactively alter upstream eligibility determinations.

To preserve the strict biological fidelity of the ensemble predictions and mathematically isolate the models from healthcare administration bias, a rigorous runtime geometric exclusion filter was deployed prior to ensemble synthesis. During the dynamic execution of the Phase III *SuperLearner*, the topology of missing data was audited row-wise within the boundaries of each cancer-specific predictive matrix (Supplementary Table S4). Crucially, the 35% topological missingness barrier was established as a strict operational policy to intercept severe bimodal sparsity patterns endemic to high-dimensional pan-cancer arrays. This conservative threshold was explicitly designed to capture and surgically excise computationally excluded vectors, namely patient samples where the absence of clinical profiling outweighs verifiable biological mass. By categorically excluding patient samples presenting with missing data spanning ≥ 35% of their explicit lineage-specific omic predictors, the architecture was computationally protected from algorithms falsely mapping extensive missingness (NA) into artificial default trajectories of protective or deleterious risk.

Patients exhibiting partial missingness (< 35%) were retained to preserve the topological density of the cohort matrix. For these retained samples, missing data were systematically managed according to the mathematical structure of the terminal algorithms: tree-based gradient boosting (*XGBoost*) actively utilized sparsity-aware split finding to incorporate missingness patterns natively without data alteration. Concurrently, *RSF* derived out-of-bag (OOB) proximity consensus to execute dynamic topological imputation at individual tree nodes. To prevent matrix singularity in linear modeling modules (*MTLR*), remaining partial sparsity was definitively resolved using multi-layered non-linear hierarchical imputation (powered by the *missForest* algorithm) prior to dimensional shrinkage.

We deployed a *SuperLearner* Architecture (*RSF*, *XGBoost*, *MTLR*, and Survival-*Boruta*) not to optimize an arbitrary accuracy metric singularly, but to benchmark and evaluate the multi-omic survival topology from four distinct mathematical dimensions. While *XGBoost* explores high-order non-linear epistatic interactions, *RSF* establishes a rigorous OOB robustness baseline, and *MTLR* discretizes longitudinal hazard to capture temporal biological shifts. Concurrently, Survival-*Boruta* functions as a master topological assessor to decisively isolate true omic drivers from stochastic noise. Together, they ensure our topographical mapping of patient survival is neither algorithm-biased nor blind to proportional hazard violations.

### 2.14 Phase III algorithm 1: *RSF*–based inference for structurally non-proportional hazards (non-PH) survival geometry

As the first implemented Phase III algorithm, *RSF* was used to perform supervised survival inference for all TAR-admissible and modelable cancer–endpoint strata classified as structurally incompatible with proportional hazards but modelable (Regime C) in Phase II. *RSF* was selected as the initial Phase III modeling framework due to its ability to accommodate non-linear, interaction-rich, and potentially time-varying survival structures without imposing proportional hazards, linearity, or sparsity assumptions, thereby aligning directly with the survival geometry diagnosed in Phase II.

*RSF* models were derived independently within each fixed execution unit defined by the tuple (*c*, *m*, *d*) representing (*cancer type*, *survival endpoint*, *TAR-admissible preprocessing regime*), with cohort composition unchanged from Phase II. No sample exclusion, feature filtering, or preprocessing modification was permitted at this stage. Predictor matrices were used exactly as passed from Phase II feasibility repair, ensuring strict separation between inferential modeling and upstream data handling.

Model derivation followed a fully deterministic and auditable protocol. All random components were controlled through fixed seeds, and model hyperparameters were specified *a priori* and held constant across strata. Trees were grown using survival-specific splitting criteria based on event-time information, and ensemble aggregation was used to estimate subject-specific survival functions and cumulative hazard functions. No hyperparameter tuning, adaptive depth control, or performance-driven model selection was performed.

The outputs of *RSF* modeling consisted of individual-level survival function estimates, cumulative hazard estimates, and time-indexed risk rankings at pre-specified horizons. Variable importance measures were computed for descriptive purposes only and were not used to drive feature selection, preprocessing revision, or downstream eligibility decisions. *RSF* outputs were recorded as Phase III artifacts and were not permitted to influence TAR classifications, Phase II feasibility outcomes, or cohort definitions.

Despite its computational efficiency, deploying standard tree architectures (such as ranger) as a primary predictive base-learner was explicitly rejected. These architectures require fully finite predictor matrices, and using them would have necessitated either severe listwise sample deletion or forced parametric imputation of highly sparse omic variables (>35% groupwise missingness) that were deliberately locked and protected within their specific cohorts during Phase I. This would have compromised the rigorous geometric preservation established and audited in Phase II. Consequently, the randomForestSRC implementation was exclusively locked in as the definitive Phase III *RSF* baseline engine (Ishwaran et al., 2008).

By natively mapping and resolving missingness directly within its dynamic tree topology (via adaptive in-node splitting), randomForestSRC seamlessly resolved the residual NA values in the highly sparse omic layers that Phase I had purposefully insulated from imputation on a strict cohort-by-cohort basis. This native topology perfectly preserved the exact mathematical geometry of the matrices, as validated by Phase II admissibility criteria, and enabled robust non-linear inference without introducing any sample deletion.

Ranger was entirely restricted to the insulated *Boruta* topological assessor. To ensure optimal matrix health uniformly across functionally brittle linear modules, a localized *missForest* wrapper was deployed strictly within both the *Boruta* assessor and the *MTLR* optimization sequence, effectively insulating the primary *RSF/XGBoost* survival matrix from imputation contamination.

### 2.15 Phase III algorithm 2: gradient boosting survival (*XGBoost*)

To establish a comprehensive, non-parametric methodological triangulation, additional ML architectures were deployed sequentially under the exact same strict, TAR-compliant execution protocols as *RSF*. *XGBoost* was applied as an iterative optimization algorithm. By sequentially constructing targeted decision trees driven strictly by the gradient of the survival loss function, the boosting architecture dynamically isolated hyper-specific prognostic omic patterns within subtle, high-risk patient subpopulations. This architecture strictly prohibited cross-disease data leakage or dynamic cohort structural modification.

### 2.16 Phase III algorithm 3: survival-*Boruta* as a predictive topographical assessor

Executing structurally as the critical third analytical tier in the Quadripartite ML Ensemble, subsequently orchestrating feature delivery for *MTLR* optimization, computationally insulated Survival-*Boruta* logic was deployed to execute rigid topographical assessment. Unlike *RSF* and *XGBoost*, which dynamically optimize predictive performance, *Boruta* operates as a strict feature selection proxy network (Kursa and Rudnicki, 2010). By duplicating the multi-omic predictor matrix into a randomized shadow-feature subspace, it constructs parallel Random Forest models to independently rank biological features based on a strict, Z-score-driven statistical separation from stochastic noise. Incorporating this *Boruta*-driven algorithmic topology directly into the Quadripartite ML Ensemble ensured that the final predictive synthesis was weighted not only by outcome accuracy but also by the irrefutable, noise-separated biological legitimacy of the underlying omic drivers.

### 2.17 Phase III algorithm 4: *MTLR*

Functioning as the fourth and final base-learner, *MTLR* was deployed. Respecting the fundamental single-task (cancer-specific) boundaries of the study, the “tasks” in this *MTLR* implementation referred specifically to distinct clinical survival intervals, permitting the simultaneous calculation of patient-specific survival trajectories across fixed temporal horizons (e.g., 1-year, 3-year, and 5-year time horizons).

To prevent matrix singularity and Fortran execution crashes in highly sparse, high-dimensional (P > N) cancer strata, *MTLR* optimization was computationally orchestrated to ingest the topological map validated by *Boruta*. Prior to model initialization, the multi-omic predictor matrix was strictly filtered to include only the subset of features definitively validated (Confirmed or Tentative) by the isolated *Boruta* sub-network. Furthermore, to preserve high-fidelity non-linear topologies, this localized *Boruta* matrix was dynamically passed through a localized *missForest* wrapper rather than a primitive median-fill, preventing artificial variance shrinkage. Finally, execution was natively safeguarded using an Elastic L2-Penalization protocol (cv. MTLR), enforcing programmatic ridge shrinkage to ensure mathematical convergence of the survival trajectories without multicollinear parameter explosions.

### 2.18 Phase III universal benchmarking and performance metrics

To universally evaluate discriminatory capacity without bias toward any single algorithmic architecture, structural performance was assessed across all Phase III models (*RSF*, *XGBoost*, and *MTLR*) using the macro-averaged C-index. Furthermore, to assess time-resolved predictive performance, time-dependent receiver operating characteristic (ROC) curves and their corresponding areas under the curve (AUC*(t)*), derived from cumulative/dynamic ROC analysis (Heagerty and Zheng, 2005), were explicitly computed and extracted at 1-, 3-, and 5-year survival time horizons for each modelable stratum. This unified testing framework ensured strict mathematical comparability across structurally distinct ensemble components.

To ensure robust execution and eliminate manual data handling artifacts during severe machine dropouts, all structured execution outputs were programmed using a real-time thread-safe Atomic Logging mechanism. As the asynchronous computational nodes completed their isolated topologies, the Quadripartite metrics were dynamically injected into a centralized Master Output Matrix string simultaneously, bypassing the need for post-run directory sweeping and ensuring that 100% of successfully computed cohorts were permanently retained in the master index.

### 2.19 Phase III meta-algorithm: multi-view meta-learner (*MVL*) *SuperLearner*

To systematically synthesize the divergent inductive biases of the Quadripartite ML Ensemble, the predictive vectors from the four individual base-learners were integrated via an Elastic Net Multi-View Meta-Learner (*MVL*). Crucially, because Survival-*Boruta* operates natively as a categorical feature selection proxy network rather than a continuous survival estimator, a formal topological conversion was required prior to *MVL* integration. Upon completing its shadow Z-score analysis, the highly condensed subset of biologically validated features (Confirmed and Tentative) was isolated and injected into a dedicated, independent *RSF* engine. This independent *RSF* module, derived strictly from the noise-separated *Boruta* topology, generated the continuous out-of-bag (OOB) survival vectors that represented the ‘*Boruta*’ view within the *MVL* synthesis. The resulting continuous predictions from all four frameworks (*XGBoost*, *RSF*, the *Boruta*-proxy network, and *MTLR*) were then systematically weighted by the *MVL* Elastic Net to establish the ultimate pan-omic patient survival trajectories. To ensure rigorous evaluation across the entire 96-cohort topological space without omitting challenging biological strata, the Phase III architecture incorporates strict, algebraically neutral computational fallbacks against algorithmic convergence failure or omic noise (termed the ‘No One Stays Behind’ policy; for a complete description of the deterministic *MVL* imputation, baseline coercion rules, and the exact two-layer architecture, see Supplementary Note 4). Importantly, the *MVL* functions as a *post-hoc* penalized synthesis rather than a traditional stacked generalization framework. The four base-learners are trained independently once on the full multi-omic matrix; their per-patient risk scores populate a 4-column meta-matrix; Elastic Net regularization is then applied exclusively to this compact meta-matrix to penalize extreme predictions and stabilize the ensemble consensus.

### 2.20 Phase III model evaluation and internal validation

To preserve the maximum geometric density of our clinical cohorts and avoid structural collapse during high-dimensional splitting, strict hold-out testing (e.g., train/test data splitting) was mathematically unfeasible and therefore not utilized for the Phase III pipelines. Instead, model performance, quantified by the Concordance Index (C-index) and time-dependent Area Under the Curve (timeROC), was evaluated using algorithm-specific internal validation and bootstrapping techniques.

Base-learners utilizing tree-based topologies natively supported OOB validation; specifically, *RSF* and Survival-*Boruta* architectures reported performance metrics calculated exclusively from their respective OOB bootstrap samples, providing robust out-of-sample estimations. Conversely, *XGBoost* and *MTLR* metrics were extracted as apparent performance across the full cohort space to map the absolute biological derivation limits and highest-order non-linear interactions, inherently capturing peak matrix overfitting.

To synthesize these divergent metrics without succumbing to “winner-takes-all” overfitting, the final *MVL* Elastic Net *SuperLearner* utilized dynamic internal *K*-fold cross-validation. The cross-validation structure defaulted to 10 folds (*K* = 10), dynamically adjusting down to a minimum of 3 folds (*K* = 3) for strata suffering from extreme event sparsity (total events < 30). This rigorous internal cross-validation ensured that the ultimate integrated prognostic risk mapped by the *SuperLearner* represented a penalized, highly conservative, and generalizable biological consensus. This internal cross-validation was applied exclusively to the 4-column meta-matrix for the purpose of *λ* selection; individual base learners were not retrained within this procedure. The *MVL* therefore functions as a *post-hoc* synthesis, with cross-validation serving only to optimize the Elastic Net penalty parameter rather than to generate out-of-fold predictions for meta-learner training.

### 2.21 Phase III *post-hoc* clinical calibration and topological alignment audit

To rigorously validate the absolute prognostic accuracy of the multi-omic survival ensembles without introducing algorithmic re-fitting bias, a post-hoc calibration audit was executed across all 96 modelable strata. Strict clinical calibration was assessed for the complete Quadripartite ML Ensemble (*RSF*, *XGBoost*, Survival-*Boruta*, and *MTLR*) and the *MVL SuperLearner* using the Time-Dependent Brier Score at established clinical landmarks (1, 3, and 5 years) and the Integrated Brier Score (IBS) across the entire follow-up period. Because survival datasets inherently contain right-censored observations, the probabilistic prediction errors were adjusted using Inverse Probability of Censoring Weighting (IPCW) via Kaplan–Meier estimations of the censoring distribution.

To ensure absolute topological integrity between the original multi-omic reference matrices and the final predicted survival curves, a geometric mapping bypass was computationally enforced. This mechanism systematically isolated and corrected internal algorithmic dropout events, such as implicit C-backend dropping of non-positive survival times during *RSF* evaluation, ensuring that all generated risk vectors mapped successfully back to their respective TCGA patient barcodes. The final extracted survival probability matrices successfully captured the temporal decay of predictions across the individual base learners and computationally validated the highly polarized prognostic stratification generated by the *MVL SuperLearner*. Detailed mathematical formulations of the IPCW Brier extraction and topological bypasses are provided in Supplementary Note 5.

### 2.22 Phase III *post-hoc* interpretability: *SHAP* and *LIME*-based feature attribution

Because the Phase III modeling framework explicitly prioritizes models capable of accommodating non-proportional, non-linear, and interaction-rich survival geometries (e.g., *RSF*, *XGBoost*, *MTLR*), the resulting predictions fundamentally lack the natively interpretable coefficients characteristic of linear proportional hazards models. To extract actionable biological and clinical insights without retroactively imposing false linearity or simplistic structural assumptions on the data, *post-hoc* interpretable ML (iML) algorithms were applied exclusively as explanatory layers to the frozen outputs of Phase III models.

This *post-hoc* interpretability framework utilizes *SHAP* and *LIME* to systematically decouple model execution from variable attribution. Neither *SHAP* nor *LIME* values were used for automated feature selection, retroactive filtering of the predictive matrices, or alteration of the upstream *TAR-admissible* preprocessing cohorts, thereby preventing selection bias and circular optimization.

#### 2.22.1 Individual-level surrogate explanations via *LIME*

Complementarily, *LIME* was utilized to provide localized, model-agnostic surrogate explanations for specific patient trajectories. Instead of attempting to explain the global architecture of the non-linear *SuperLearner*, *LIME* fits a sparse, intrinsically interpretable linear surrogate model in the immediate local neighborhood of a specific patient’s predictor profile. This approach provides “point-of-care” transparency, generating a localized coefficient weighting that explicitly maps which clinical and omic characteristics predominantly drove a highly specific survival prediction (e.g., a severe upward deviation in a patient’s endpoint-specific longitudinal hazard) (Supplementary Note 6).

#### 2.22.2 Global and local attribution via *SHAP*

*SHAP* was employed to compute the precise marginal contribution of each multi-omic predictor to the model’s output, grounded in cooperative game theory. By calculating *SHAPley* values for the derived Phase III ensembles, we obtained both local (patient-specific) and global (cohort-wide) feature attributions.

#### 2.22.3 Global interpretability

To understand the macro-level drivers of survival within a given cancer–endpoint stratum (*c*, *m*, *d*), strict *SHAP* values were aggregated to rank multi-omic signatures by their overall inferential importance. To further explore topological risk, *SHAP* dependence plots were generated to visualize nonlinear phenomenological threshold effects. Crucially, multi-omic interactions were mathematically evaluated using 3D *SHAP* interaction matrices to dynamically highlight the strongest biological synergies that were captured by the ensemble frameworks but remained unobservable through traditional covariance metrics.

To rigorously classify the topological nature of these cross-omic interactions, a strict mathematical thresholding framework was implemented. Pairwise dependencies were computationally categorized into mutually exclusive functional classes based on Spearman’s rank correlation coefficient (*ρ*), which maps the primary feature**’**s physical abundance to the *SHAP* interaction magnitude of the secondary feature. To control for false positives across the extensive pan-cancer interaction tensor, all raw *p*-values were adjusted using the Benjamini-Hochberg False Discovery Rate (FDR) procedure. Interactions were classified as “Synergism (Hazard Amplification)” if they demonstrated a strong positive correlation (*ρ* ≥ +0.30, FDR < 0.01) in the upper quartile of the primary feature**’**s expression. Conversely, interactions were classified as “Antagonism (Rescue Effect)” if they exhibited a strong inverse topology (*ρ* ≤ −0.30, FDR < 0.01). Non-linear trajectories that failed to reach significance at the distributional extremes but demonstrated robust cross-talk within the middle 50% expression tier were classified as “Context-Dependent Bifurcations” (*ρ* ≥ 0.30, MidZone FDR < 0.01). Topologies that did not meet these stringent geometric and statistical magnitude constraints were excluded as non-significant contextual noise. These classifications are strictly mathematical descriptors of the *SHAP* interaction direction (positive Spearman *ρ* = same-direction effect on the predicted event probability; negative *ρ* = opposing-direction effect) and represent model-internal geometry rather than independently validated biological mechanisms. The joint FDR threshold (FDR < 0.01, *ρ* ≥ 0.30) applied to all 26,800 reported interactions also serves as an explanation stability safeguard: weak or unstable associations are excluded from classification, ensuring that only statistically robust interaction topologies are retained.

#### 2.22.4 Mathematical isolation of dominant *SHAP* interactions

To decode the non-proportional hazard geometries mapped by the *XGBoost* module, we executed an exhaustive extraction of *SHAP* interaction values. The ensemble initially computed a comprehensive 3D interaction tensor, capturing every potential pairwise synergistic or antagonistic relationship across the multi-omic feature space. However, to prevent the topological output from degenerating into a saturated network of secondary associations, we imposed a strict mathematical dominance constraint during the extraction phase. For each primary signature identified, the algorithm programmatically isolated the single strongest interacting partner (the “apex” interactor) by enforcing a maximum strength constraint, thereby deliberately discarding all weaker secondary or tertiary pairings. This strict 1:1 topological restriction ensures that the resulting interaction networks represent dominance pathways rather than overlapping associative noise, providing a clear, high-resolution hierarchical blueprint for precision oncology forecasting.

#### 2.22.5 Local interpretability

At the individual patient level, *SHAP* decision plots were utilized to dissect how specific deviations in a patient’s multi-omic profile affected their predicted mortality risk relative to the baseline cohort hazard (Supplementary Note 6).

Together, the integration of *SHAP* and *LIME* ensures that the deterministic, structurally complex survival models deployed in Phase III remain fully actionable and clinically interpretable. Translating high-dimensional, multi-omic interactions into explicit, directional risk attributions allows for deep biological validation of the identified regulatory circuitries without violating the rigid separation between the predictive mechanism and the underlying cohort geometry established in Phases I and II.

A critical objective of this multi-omic predictive framework was to achieve transparent, granular traceability of the features governing survival predictions across highly non-linear topologies. Rather than applying a blanket “model-agnostic” explainer to all ensemble components, which is often computationally prohibitive on multi-omic matrices (comprising >1,000 biological variables) and methodologically redundant, we deployed a strictly model-centric interpretability topology.

#### 2.22.6 Exact *TreeSHAP* and *LIME* via gradient boosting (*XGBoost*)

*Post-hoc* explainability mechanisms (*SHAP* and *LIME*) were anchored exclusively to the *XGBoost* algorithm. The primary justification is topological exactness. *XGBoost* embeds exact Tree-based *SHAPley* Additive exPlanations (*TreeSHAP*) algorithms directly within its core C++ backend, allowing for perfect, instantaneous calculation of localized and global marginal contributions for every variable without relying on synthetic approximations. Furthermore, *XGBoost*’s capacity to natively capture sparse, high-order non-linear interactions aligns perfectly with *LIME*’s mechanism, which constructs perturbed, localized linear surrogate models around individual patient coordinates to explain discrete prognostic outcomes (see Supplementary Note 4 for a complete description of algorithm-specific interpretability scoping and the distinction between base-learner and meta-learner output spaces).

#### 2.22.7 Extraction of point-of-care decision trajectories

To operationalize these findings for point-of-care precision oncology, we translated localized *TreeSHAP* feature attributions into individualized precision risk trajectories. While global *SHAP* metrics define population-level superiority, patient-level decision trajectories (visualized via waterfall and force plots) were extracted to mathematically deconstruct how combinatorial omic payloads influence a specific individual’s prognostic outcome relative to the cohort baseline. These trajectories dynamically reveal the magnitude and directional polarity of each transcriptomic, genotypic, and somatic perturbation in real-time. Consequently, this end-to-end framework bypasses algorithmic “black boxes” by anchoring high-density machine learning predictions to fully transparent, sequence-specific precision summaries tailored to each unique patient.

#### 2.22.8 Native topology in *RSF*

While model-agnostic computational equivalents (such as *KernelSHAP*) exist, applying them to an *RSF* ensemble of 1,000 trees across massive omic dimensions incurs an exponentially high computational cost. More importantly, it is scientifically redundant. *RSF* natively quantifies global feature ranking utilizing localized ensemble permutation (Variable Importance, VIMP). By utilizing native VIMP extraction, the model inherently resolves hierarchical importance based strictly on the topology of the survival tree splits, generating an interpretability array that is parallel (and complementary) to *SHAP* without incurring synthetic computational bloat.

#### 2.22.9 Explainability by design in *Boruta*

To preserve the strict geometric separation between predictive modeling and *post-hoc* feature selection, *Boruta* was executed using an insulated *missForest* (Random Forest Imputation) wrapper. Because rigid linear models and shadow algorithms mathematically require fully dense matrices to execute, localized non-parametric *missForest* imputation was strategically applied strictly within the *Boruta* and *MTLR* evaluation domains. This ensured that *Boruta* could accurately resolve survival-critical features and *MTLR* could achieve ridge-penalized convergence amidst high-dimensional sparsity without contaminating the raw, non-imputed survival geometries evaluated natively by the supervised *RSF* and *XGBoost* predictive base-learners.

The *Boruta* method was explicitly integrated not as a “black-box” predictor requiring *post-hoc* unpacking, but as a primary explainability algorithm itself. *Boruta* independently ranks features through a randomized shadow-variable infrastructure, directly evaluating absolute biological signals against mathematical noise. Layering *post-hoc* algorithms such as *SHAP* or *LIME* onto *Boruta* would be conceptually contradictory, as its innate Z-score statistical evaluation already provides robust, standalone feature verification. However, to allow this noise-separated biological logic to actively “vote” in the ultimate pan-omic survival prediction, the subset of features definitively validated by *Boruta*’s shadow metrics was subsequently mapped into an independent Random Survival Forest proxy. This enabled the categorical explainability outputs of *Boruta* to be successfully converted into continuous survival trajectories for *MVL* Elastic Net synthesis.

### 2.23 Parametric frailty in *MTLR*

Because *MTLR* maps survival through a structured sequence of penalized, pseudo-logistic models, it is inherently mathematically fragile when confronted with high collinearity and sparse mutational matrices. Deploying *LIME* around an *MTLR* schema generates synthetic, perturbed neighborhood samples, which exacerbates local collinearity and frequently results in singular matrix failures. Consequently, *MTLR* was retained purely as the structural parametric baseline for the *SuperLearner* architecture, insulated entirely from *post-hoc* synthetic perturbation.

By geometrically distributing interpretability metrics, *TreeSHAP*/*LIME* for *XGBoost*, native VIMP for *RSF*, and Shadow *Z*-Scores for *Boruta*, the framework robustly generates multifaceted biological explainability without compromising the computational scalability required for phase-level multi-omic analysis. Crucially, the *TreeSHAP* extraction protocol deployed a dual-matrix strategy. Beyond computing standard 1D marginal attributions for global beeswarm topographies, we deliberately forced the *XGBoost* backend to compute the exhaustive multi-dimensional (N × N) interaction tensors (predinteraction = TRUE). This rigorous mathematical decoupling explicitly isolates the primary marginal effect of an omic signature from the synergistic interaction effect it possesses when combined with intersecting omic variables, enabling true non-linear synergy discovery without computational collapse.

### 2.24 Phase III baseline clinical probability extraction and retention audit

To establish the baseline reference probability distributions, individualized continuous risk scores were natively extracted from the 96 Phase III *SuperLearner* derivation ensembles without data contamination or synthetic imputation. Due to the high dimensionality of the ensemble architecture, *a priori* feature-missingness thresholds were strictly enforced, resulting in the exclusion of sample patients lacking ≥ 35% of robust multi-omic predictors. Furthermore, cohorts lacking sufficient statistical events to resolve robust cross-validated survival nodes (e.g., KICH, CHOL) or demonstrating zero target gene genome-wide significance (e.g., DLBC) were excluded ab initio. Natively extracted risks were bifurcated via Cox-Breslow transformations to estimate exact clinical probabilities across 1-, 3-, and 5-year horizons. For OS and DSS endpoints, output functions mapped directly to the Survival Probability, S(t); for DFI and PFI endpoints, the inverse transformation *1 − S(t)* was applied to mathematically isolate the Probability of the event occurring by time *t*. To formally quantify the Phase III gating constraints, an algorithmic audit was conducted to calculate strict sample retention rates versus algorithmic penetrance densities across all evaluated cohorts.

### 2.25 Generation of the internal blind validation cohort

To establish a rigorous, completely unseen internal validation cohort specifically designed to test the true prognostic generalization of the multi-omic models, patient records were systematically filtered to prevent data leakage from the Phase III derivation process. The root multi-omic database was cross-referenced against the Phase III model derivation dataset (Supplementary Dataset S2). Any patient identifiers present in the master dataset but absent from the derivation dataset were extracted and sequestered. This methodology yielded an internal validation cohort comprising exactly 1,050 unique patient records spanning 27 distinct cancer types (resulting in the total biological exclusion of the severely fragmented UVM and DLBC cohorts). These sequestered records were physically withheld from all algorithmic hyperparameter tuning and Phase III Elastic Net synthesis, ensuring that the validation matrix provided a mathematically pristine test environment. Furthermore, this cohort was designated as strictly “blind” because the validation inference engine was constructed from patients who lacked documented clinical endpoints (survival status and time) in the TCGA source data. These patients had no recorded outcomes by definition, providing a true prospective deployment test environment in which risk scores must be generated from multi-omic topology alone. The specific distribution of sequestered patients and their multi-omic predictive variable dimensions per cohort is formally summarized in Supplementary Table S5.

### 2.26 Clinical blind validation, dual-track inference, and algorithmic penetrance

Following cohort isolation, the serialized Phase III models were computationally deployed against the blind validation matrix. To prevent mathematical corruption from topological fragmentation or extreme missingness inherent in real-world clinical data, the validation engine utilized a proprietary Dual-Track inference architecture. This architecture ensured strict geometrical parity with the Phase III derivation matrix by enforcing nomenclature preservation and applying exact Phase III centering and scaling constants prior to *SuperLearner* Elastic Net synthesis.

Crucially, the Dual-Track architecture was designed with a mathematical safety mechanism. For patient records containing structurally intact multi-omic signatures, the full *SuperLearner* algorithm seamlessly synthesized continuous risk hazard Z-scores (Path A). However, when the pipeline encountered severely fragmented records lacking critical variables, the *SuperLearner* dynamically aborted the synthesis, outputting an intentional non-prediction (NA) to prevent the artificial escalation of risk scores. A comprehensive mapping of these algorithmic safety abortions and missingness frequencies across all evaluated cohorts is provided in Supplementary Table S6.

To ensure that this strict safety gating did not result in clinical patient attrition, the pipeline routed all masked records into a native *XGBoost* Path B, which inherently handles missing variables without requiring synthetic imputation. As verified in the Algorithmic Penetrance Matrix (Supplementary Table S7), an unbroken computational lineage was maintained against the pristine validation cohort. The tables confirm that while the *SuperLearner* selectively masked fragmented sub-cohorts, *XGBoost* Path B ensured 100% predictive penetrance across the entire pipeline, independently calculating continuous hazard vectors across four distinct survival endpoints (OS, DSS, PFI, and DFI) without leaving a single patient unscored. For a comprehensive mathematical breakdown of the validation inference engine and its structural safety fallbacks, see Supplementary Note 7.

To bridge the gap between abstract continuous hazard *Z*-scores and actionable clinical metrics, the pipeline was engineered to extract individualized probabilities at clinically significant 1-, 3-, and 5-year landmarks. To preserve strict blinding, probability estimates were not derived from the validation cohort’s survival data. Instead, the inference engine dynamically reconstructed the original multivariable baseline hazard (Breslow estimator) from the corresponding Phase III derivation matrix. During reconstruction, exact geometric parity with the Phase III derivation matrices was enforced, rigorously anchoring zero-day follow-up patients to prevent computational artifacts. This extraction strictly adhered to the Dual-Track architecture: patients evaluated by Path A were mathematically anchored to the *SuperLearner* Elastic Net baseline, while those evaluated by Path B were anchored exclusively against the native *XGBoost* baseline. Finally, baseline hazard calculations were dynamically routed by endpoint. For OS and DSS, patient risks were computed from the Kaplan–Meier estimator *S(t)* as the probability of survival. Conversely, for DFI and PFI, the estimator was inverted *1 − S(t)* to reflect the cumulative probability of the clinical relapse or progression event occurring.

## 3 Results

To systematically deconvolute non-linear multi-omic survival topologies across diverse pan-cancer landscapes, we engineered a rigorous, multiphasic machine learning analytical pipeline consisting of three execution phases (Figure 1). A fundamental mathematical constraint strictly enforced across all phases was groupwise execution. At no point were distinct cancer cohorts or the dimensional 14,595-variable multi-omic universe merged for global computation. This isolated stratification was designed to prevent inter-lineage data leakage and perfectly preserve local cancer-specific biological architecture.

### 3.1 Phase I: structural dimensionality and multi-omic harmonization

The theoretical pan-cancer landscape originally comprised 132 unique survival strata across 33 lineages and four endpoints. However, securing the structural integrity of these isolated cohorts required stringent data harmonization, which immediately revealed 12 strata to be fundamentally uncomputable due to inherent clinical or omic limitations (Supplementary Table S8). The diffuse large B-cell lymphoma (DLBC) cohort yielded no clinically significant multi-omic signature associations, resulting in the removal of its four analytical strata. Furthermore, specific clinical endpoints, predominantly DSS and DFI, were clinically undefinable or systematically unviable across several cohorts (LAML, GBM, MESO, SKCM, THYM, and UVM; full cancer type abbreviations are defined in Supplementary Table S1), eliminating an additional eight strata. Clinical endpoints were governed by strict exclusionary policies; duplicate samples were harmonized to accurately reflect patient execution trajectories, while samples exhibiting significant voids in clinical endpoints or complete omic omissions were systematically purged.

With the clinical target geometries locked at a baseline of 120 viable cohorts (Supplementary Table S8), the inherent sparsity across the seven distinct omic predictor layers was addressed via an extensive, localized algorithmic imputation engine. By deploying a matrix of both conventional algebraic and advanced nonparametric techniques strictly within cohort boundaries, we generated 120 comprehensive LiSHMOM (Figure 1, Phase I). To ensure computational transparency, an unbroken data provenance architecture was constructed, utilizing acyclic lineage logic (visualized via a targeted Sankey hierarchy in Supplementary Figure S2; Figure_S2_HTML) to seamlessly trace any generated LiSHMOM matrix directly back to its distinct data-handling intervention.

### 3.2 Phase II: structural feasibility and survival geometry auditing

Operating on the 120 harmonized LiSHMOM inputs, Phase II functioned exclusively as a geometrical diagnostic rather than a predictive exercise. We deployed *CoxNet* as an independent CANARY adjudicator to mathematically probe the structural admissibility of proportional hazards (PH) geometry within each cohort (Figure 1, Phase II). This diagnostic revealed a rigorous two-tiered failure cascade driving cohort attrition.

The first tier of failure was characterized by strict baseline structural infeasibility. As validated against the non-imputed df005 reference matrix (Supplementary Table S9), cohorts such as CHOL (*N* = 45) and DLBC (*N* = 47) were inherently disqualified for violating strict macro-level sample size constraints (< 50 viable samples) or for experiencing total predictor opaqueness (absence of multi-omic mapping).

The second tier of failure emerged when securing a robust baseline geometry did not ensure dynamic algorithmic convergence. When evaluating the initially robust cohorts under fixed identifiability thresholds against specific survival metrics (OS, DSS, PFI, DFI), CANARY identified severe survival geometry failures. These manifested as either explicit target scarcity (yielding < 20 valid survival events per cohort slice) or severe *μ*-ladder exhaustion; a strict inability to achieve stable model convergence despite highly permissive regularization relaxations (Supplementary Figure S3). The latter closely correlated with extreme multi-omic collinearity and underlying feature sparsity observed in the baseline matrix (e.g., UCS harboring only 1 variable; UVM harboring 13 variables), rendering sparse penalized inference mathematically unattainable (Supplementary Figure S4).

An independent Schoenfeld residual audit of the 120 CANARY-tested strata confirmed pervasive proportional hazards violations. Standard unpenalized Cox models converged on 118 of 120 strata, but the high-dimensional multi-omic predictor matrices exceeded the computational capacity of the Schoenfeld residual test in 80 cases, an inherent limitation of parametric PH diagnostics in pan-cancer settings. Among the 16 strata yielding valid Schoenfeld results, 6 demonstrated statistically significant violations (OV-DSS, *p* < 0.001; OV-OS, *p* < 0.001; OV-DFI, *p* < 0.001; ESCA-PFI, *p* < 0.001; LAML-OS, *p* < 0.001; ACC-PFI, *p* = 0.015), while the remaining 10 showed non-significant global *p*-values (median *p* = 0.6458). These independent parametric results corroborate the CANARY diagnostic’s convergence-based finding that proportional hazards assumptions are structurally inadequate for pan-cancer multi-omic survival modeling. Complete per-stratum Schoenfeld results are provided in Supplementary Table S8.

Dissecting these CANARY outcomes across the 120 available LiSHMOMs decisively demonstrated that the widespread violations of proportional hazards assumptions were not statistical anomalies, but inherent geometric realities of high-dimensional, highly sparse omic spaces heavily constrained by survival thresholds. Ultimately, this mathematically enforced, dual-layered audit disqualified 24 hopelessly unstable cohorts from the initial 120 available matrices, safely routing a highly stabilized core of 96 algorithmically viable configurations into Phase III for necessary non-linear and non-proportional topological assessment (Supplementary Table S8).

### 3.3 Phase III: quadripartite ML ensemble topologies and the global topological stabilization of the *MVL SuperLearner*

To mathematically accommodate the severe proportional hazards violations and high-dimensional collinearity identified in Phase II, the 96 robust LiSHMOM structures were integrated into a highly parallelized Quadripartite ML Ensemble (Figure 1, Phase III). This framework processed each isolated cohort through *RSF* (assessing native topological proximities), sparsity-aware *XGBoost* networks, robust *Boruta* shadow-feature algorithms, and a mathematically dependent *Boruta*-gated *MTLR* baseline, significantly reducing single-algorithm bias and variance inflation.

The independent predictions derived from the four non-linear base learners were dynamically integrated using the *MVL* to navigate the highly fragmented dimensional space across the 96 strictly isolated survival cohorts. Maintaining the strict zero-leakage policy established in Phase I, predictive performance was evaluated within the boundaries of each targeted execution unit (cancer type × specific survival metric) rather than being artificially consolidated into a generic pan-cancer average.

Across this expansive distribution of independent topologies, the synthesized *SuperLearner* architectures demonstrated exceptional stabilization. Individual cohort concordance indices (C-index) peaked at a maximum of 0.990, with the central density of the 96 independent executions concentrating within an overarching interquartile range (IQR) of 0.685 to 0.836 (Supplementary Table S10). Bootstrap 95% confidence intervals for the *MVL SuperLearner* C-index (1,000 resamples per stratum) were computed at all three Breslow time horizons (1-year, 3-year, and 5-year). The median CI width was 0.052 at 1 year across 90 valid strata (six DFI/PFI strata excluded due to Breslow baseline hazard collapse at early time points). Results for all three horizons are reported in the expanded Supplementary Table S10. A consolidated performance summary across all 96 strata, including C-index, bootstrap confidence intervals, time-dependent AUC, permutation *p*-values, calibration slope, and Brier Scores, is provided in Supplementary Table S11.

When examining the internal algorithmic hierarchy within the limits of each execution unit, the *SuperLearner* integration formally stabilized and regularized the isolated base topologies (*RSF*, *XGBoost*, *Boruta*, *MTLR*). Rather than simply maximizing raw apparent concordance, which can lead to extreme overfitting artifacts frequently observed in isolated *XGBoost* matrices (Supplementary Table S10), the *MVL* explicitly operated as a topological brake. By systematically penalizing these artificial gradient peaks, the overarching architecture successfully compressed predictive variance across 76.0% of the specific targeted pathways, effectively mitigating local single-algorithm hallucination. Specific high-dimensional cohorts, notably READ-OS and CESC-DFI, achieved remarkable local predictive horizons, yielding native C-indices of 0.990 and 0.956, respectively.

Furthermore, explicit temporal stability was maintained locally across the cohorts. Cumulative/dynamic execution across AUC(*t*) yielded robust distribution ranges at 1-year (0.523–1.000), 3-year (0.590–0.996), and 5-year (0.571–1.000) time horizons (Supplementary Table S10). Ultimately, the deployment of this Quadripartite array demonstrated that non-linear architectures decisively outperformed predictive topologies over strictly parametric components within 100.0% of the isolated biological matrices, explicitly validating the necessity of the Phase II CANARY diagnosis (Supplementary Table S8).

**Figure 2:**
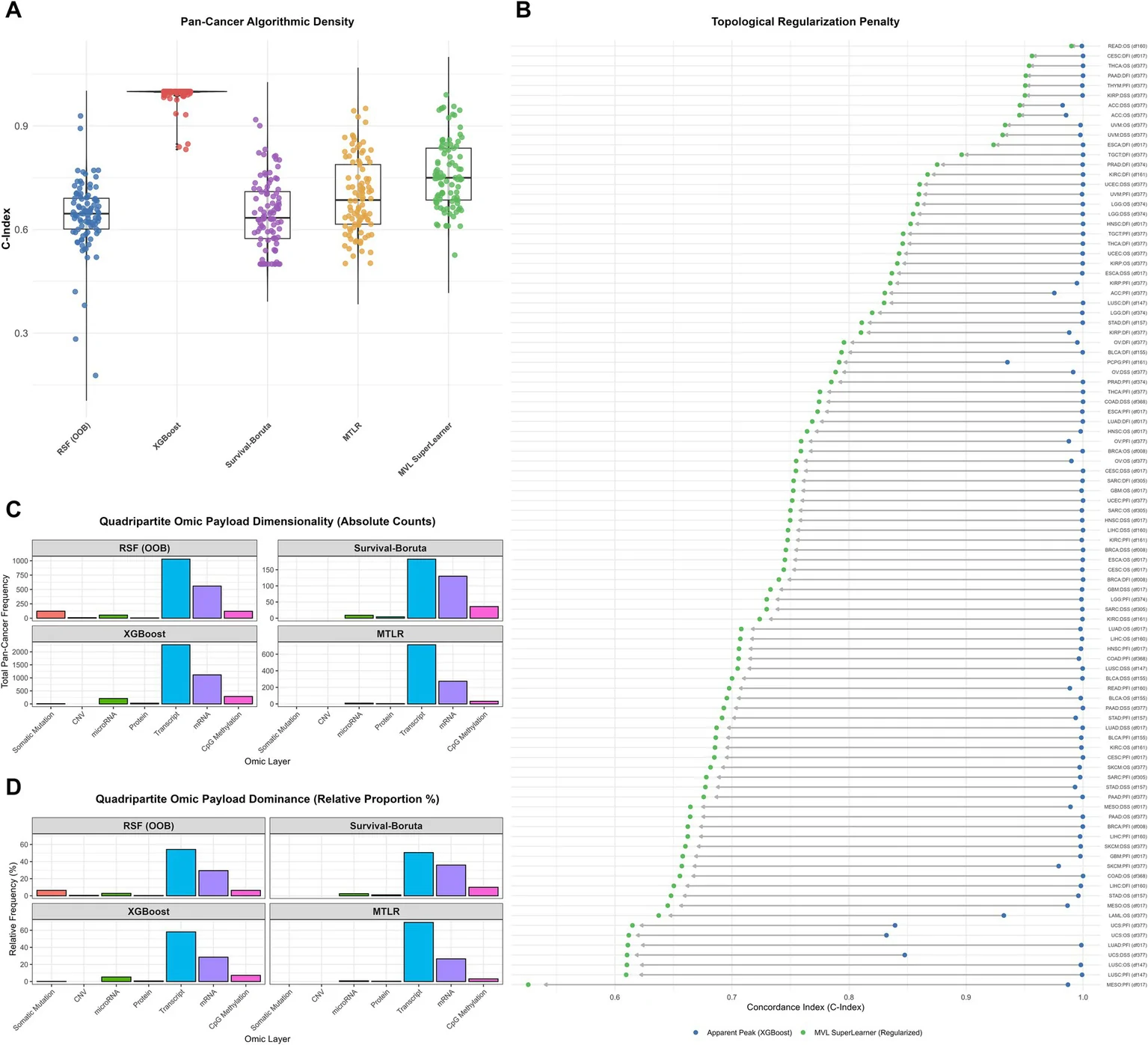
Multi-omic dominance, algorithmic geometry, and topological regularization. Comprehensive multi-dimensional evaluation of the Phase III machine learning architecture. The overarching layout explicitly traces the mathematical regularization algorithms used to address high-dimensional tree-based overfitting (Panels **A,B**), seamlessly connecting to the subsequent biological selection matrices that establish the absolute predictive dominance of RNA-based signature features over static genotypic variables across all 96 clinical survival cohorts (Panels **C,D**). **(A)** Algorithmic geometry comparison (density vs. dispersion). Strict statistical density curves (Jittered Boxplots) contrasting the rigorous topological stability of the *MVL* Elastic Net *SuperLearner* against the extreme, heavily dispersed variance of isolated base learners across all survival landscapes. Rather than optimizing for artificial numerical peaks, the distribution physically maps the *SuperLearner*’s capacity to consolidate volatile clinical architectures into a highly stable, mathematically generalized predictive space. **(B)** Topological regularization and mitigation of algorithmic overfitting. A multi-dimensional dumbbell trace evaluating the mathematical penalty enforced by the *SuperLearner*. The blue loci designate the maximum apparent peak achieved by the isolated base learners, overwhelmingly dictated by the extreme structural overfitting of Sparsity-Aware *XGBoost* at the artificial ~1.000 concordance boundary. The green loci represent the terminal, synthesized predictions constructed by the *MVL* Elastic Net. The bridging segments (directional arrows) visually map the absolute shrinkage penalty enforced to explicitly eliminate high-dimensional algorithmic overfitting. By structurally shrinking the matrices, the Elastic Net pulls the artif*i*c*i*al peaks leftward into a generalized, biologically plausible cross-validated stability space. By continuously enforcing this systemic regularization (median shrinkage penalty Delta −0.247), the trace empirically validates that the Phase III architectural fusion successfully addresses tree-based overfitting across the entire pan-cancer domain. **(C)** Quadripartite omic payload dimensionality. Absolute retention frequencies of the multi-omic topological features extracted from the native architectures of Random Survival Forest (OOB), Sparsity-Aware *XGBoost*, Survival-*Boruta*, and Multi-Task Logistic Regression (*MTLR*). The quadripartite machine learning architectures intrinsically force competitive interaction between seven distinct biological layers. **(D)** Topological dominance of RNA signatures. Relative proportional dominance of the biological axes within each machine learning topology. Across all isolated evaluations, the non-linear algorithms mathematically suppress static genotypic variables (Somatic mutations and CNVs) in favor of hyper-dimensional transcriptomic elements. Collectively, overarching mRNA abundance (30.9%) and isoform-level structural predictors (54.8%) massively dominate the high-dimensional predictive matrices, composing 85.7% of the total algorithmic survivorship vectors globally.

**Figure 3:**
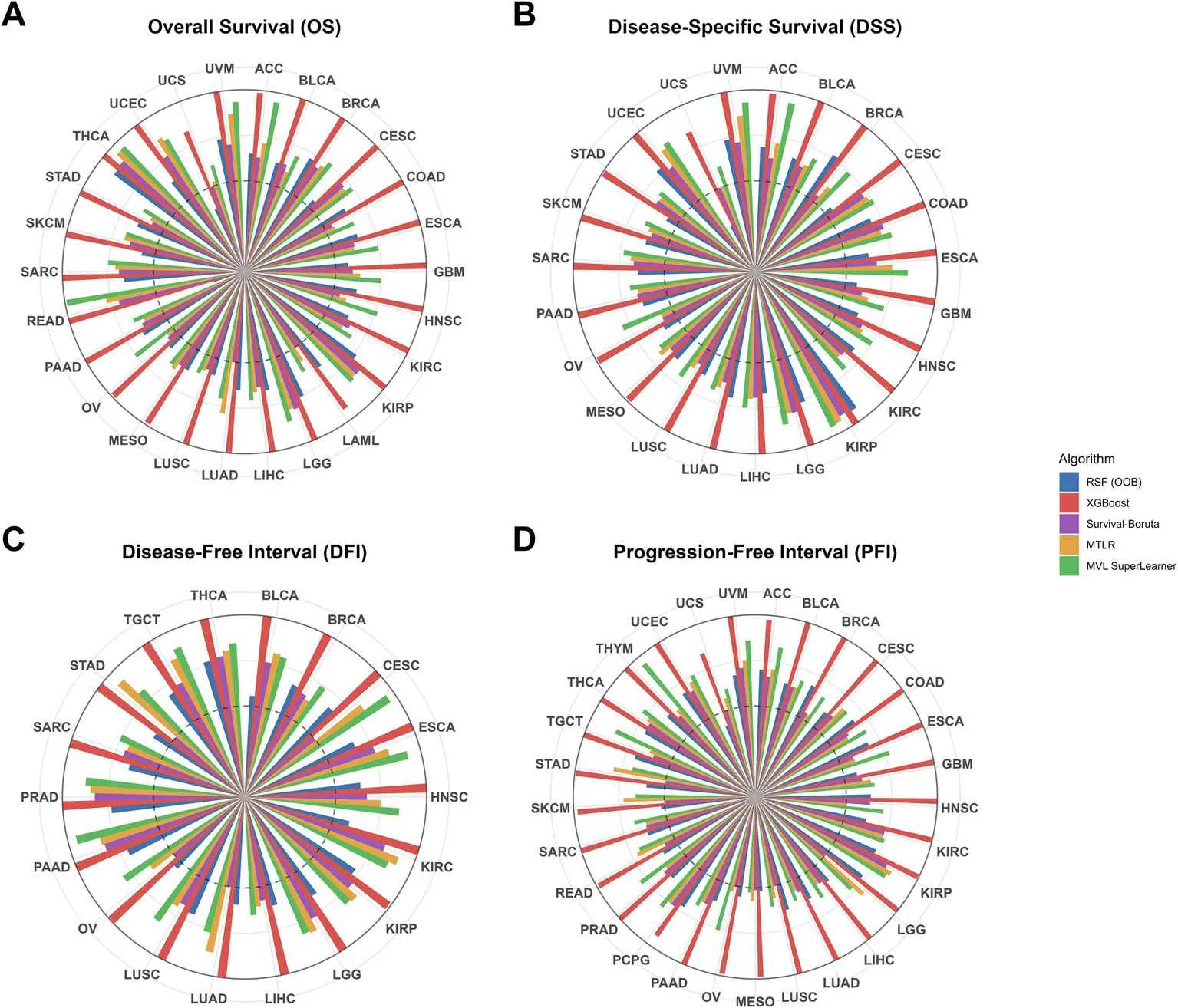
Phase III global performance panorama and algorithmic concordance benchmarking. This composite visualization evaluates the global structural predictability of the survival models synthesized across all 96 computationally viable cancer–endpoint topologies. The central visualization consists of a 2×2 multi-dimensional Radial Atlas, charting the individual algorithmic concordance (C-index) footprints generated by the four isolated base learners prior to synthesis: Random Survival Forests (blue), Extreme Gradient Boosting (red), Survival-*Boruta* (purple), and Multi-Task Logistic Regression (gold). Each quadrant of the 2×2 Atlas isolates a distinct clinical survival tracking objective: **(A)** Overall Survival (OS), **(B)** Disease-Specific Survival (DSS), **(C)** Disease-Free Interval (DFI), and **(D)** Progression-Free Interval (PFI). The radial axes define performance trajectories mapped against the specific TCGA cohorts applicable to each endpoint, allowing visual diagnosis of algorithm-specific failure points and strengths. Accompanying the Atlas, probability density distributions compare the survival tracking capabilities of these algorithms against the terminal Multi-View Meta-Learner (*MVL* Elastic Net *SuperLearner*, mapped in green). The density geometries demonstrate that rather than defaulting to isolated linear paths, fusing the distinct inductive biases (e.g., sparsity-aware routing, *Boruta* thresholding, and proximity forests) into the Elastic Net *SuperLearner* mathematically elevates and compresses the global concordance apex across all four survival intervals. Ultimately, this visualization maps how the ensemble architecture mitigates individual algorithmic hallucination to construct mathematically superior, highly stabilized survival predictions (green contours) across profoundly non-linear multi-omic topologies.

To map the global structural predictability of the Phase III cohorts, survival routing algorithms were benchmarked across the 96 structurally viable cancer–endpoint strata using the Concordance Index (C-index). Initial profiling of the isolated base learners exposed severe mathematical volatility dependent entirely on the specific clinical geometry evaluated. For instance, while *RSF* provided a median OOB concordance of 0.6458, it was intrinsically vulnerable to edge-case topological distortions, with its predictive capability occasionally crashing into negative geometries far worse than a random coin toss (minimum C-index: 0.177). Conversely, sparsity-aware architectures like Extreme Gradient Boosting (*XGBoost*) successfully optimized high-dimensional matrices but exhibited extreme overfitting typical of tree variants mapped to biological derivation limits (median Apparent C-index: 0.9992; Range: 0.832–1.000) (Figure 2A). Similarly, the independent *Boruta*-proxy *RSF* network, which generated continuous survival trajectories from the categorically validated shadow-feature subsets, was formally benchmarked across all strata, yielding robust predictive concordances (visualized as the purple topological contours in Figure 3 Radial Atlas).

Rather than defaulting to a singular “winner-takes-all” modeling topology—a strategy fundamentally dangerous given the substantial heterogeneity of the pan-cancer omic landscapes—the *MVL* Elastic Net *SuperLearner* was executed to structurally penalize and synthesize these divergent inductive biases. The Elastic Net architecture explicitly sacrificed the artificial, overfitted peaks generated by *XGBoost*, instead favoring penalized, cross-validated consensus stability.

Consequently, the *SuperLearner* compressed the global survival predictions into a highly rigorous, biologically plausible probability space across all endpoints (OS, DSS, PFI, and DFI). The synthesized *MVL* achieved a stable median global concordance of 0.749. More critically, the *SuperLearner* functioned as a catastrophic floor defense mechanism: while individual algorithms routinely collapsed or failed to resolve distinct non-linear matrix clusters, the *SuperLearner* successfully anchored the mathematical floor at 0.526, effectively ensuring that no surviving cohort was left behind and that no single algorithmic failure point could corrupt the downstream individualized interpretability pathways.

### 3.4 Topological regularization and the structural mitigation of algorithmic overfitting

To precisely quantify the topological intervention exerted by the *MVL SuperLearner*, algorithmic performance was geometrically mapped using a Dumbbell Trace visualization (Figure 2B). Within this geometry, the linear distance between the highest independent base learner (grey locus) and the fused *MVL SuperLearner* (green locus) visually captures the mathematical penalty or boost enforced by the Elastic Net master architecture.

Critically, when benchmarked against the absolute apparent limits, driven pervasively by the extreme structural overfitting of Sparsity-Aware *XGBoost* peaking at artifactual ~1.000 concordance bounds, the Dumbbell trace visually demonstrates systemic mathematical shrinkage. Rather than artificially masking the inherent bias of high-dimensional tree variants operating in sparse omic topographies, the visualization explicitly exposes it. The *MVL SuperLearner* categorically refused to validate these artifactual peaks, systematically applying a regularization penalty that pulled the overfitted base learners back into a generalized, biologically plausible concordance space (median shrinkage penalty of Delta −0.247). Consequently, the Dumbbell trace empirically confirms that the primary function of the Phase III ensemble is not to chase artifactual maxima, but to actively suppress high-dimensional overfitting, engineering a robust, mathematically verifiable lift grounded in penalized topology. Permutation testing (1,000 iterations per stratum) confirmed that both the *MVL SuperLearner* (*p* < 0.001 in 90/96 strata, 93.8%) and *XGBoost* (*p* < 0.001 in 96/96 strata, 100%) significantly exceeded chance-level concordance (null median C-index approximately 0.50), with the *MVL* systematically reducing the *XGBoost* inflated apparent C-index values by a median of +0.116 across all strata (Supplementary Table S12).

### 3.5 The terminal harvester: omic payload dimensionality and the algorithmic erasure of genotypic variables

To map the biological axes anchoring the *MVL* predictions, the internal selection logics of the Quadripartite topology were parsed to evaluate pan-cancer multi-omic dominance (Figure 2C). Across the 96 evaluated cohorts, non-linear mathematical filtration induced a severe paradigm shift in omic payload dimensionality. The architectures systematically suppressed static genomic variables in favor of highly dimensional, dynamic downstream phenotypic elements.

Globally, structural transcriptomics (isoform-level expression) emerged as the primary predictive layer, composing 54.8% of the total retained feature space, followed closely by overarching mRNA abundance metrics (30.9%). Combined, active RNA-level phenotypic signatures monopolized an overwhelming 85.7% of the algorithmically selected feature topologies driving survivorship (Figure 2D). Crucially, the non-linear architectures overwhelmingly rejected traditional genotype architectures during high-dimensional competition. Somatic mutations and CNV, historically prioritized in parametric clinical forecasting, were algorithmically reduced to near-zero retention frequencies (0.6 and 0.7%, respectively), proving mathematically incapable of independently supporting overarching temporal survival topologies when forced to compete with their downstream transcriptomic consequences.

### 3.6 Algorithmic displacement of binary genotype signatures

A fundamentally provocative outcome of the Quadripartite mapping was the systemic architectural suppression of genomic layer topologies [signatures where omic features are somatic mutations (Token `0.2`) and CNV (Token `0.3`)] by the ensemble across the pan-cancer map. Evaluating structural genotype markers across this massive multi-omic reduction reveals a structured algorithmic displacement rather than total immediate failure. Of the strict 14,595 initial signature dimensions provided to the networks, exactly 831 were somatic mutations and 737 were CNV natively. When the machine algorithms condensed the pan-cancer map into the 12,613 baseline prognostic subset, only 142 mutation-based signatures successfully passed extraction (a 17.08% persistence rate), while only 196 CNVs retained statistical validity (a 26.59% retention rate) (Supplementary Table S13).

While these proportions confirm that a localized subset of genomic anomalies successfully maintains baseline prognostic linkage, their predictive capability completely collapses under extreme multidimensional pressure. Within the strict highest echelon of the Quadripartite predictive hierarchy (the 150 golden anchors demonstrating definitive prognostic association validating across all four algorithms simultaneously, Supplementary Table S14), exactly zero genotypic features remained (0.0%).

This hierarchical suppression is directly linked to the structural tree logic inherent to our non-linear predictive topographies (such as *RSF* and *XGBoost*), which maximize predictive gradients. In a high-dimensional competitive matrix, sparse binary state representations (a mutation is strictly present ‘1’ or absent ‘0’) exhibit markedly low predictive entropy. Because they only split a patient cohort precisely once, binary vectors functionally lack the continuous topological variation required to track hazard trajectories over multi-year clinical timelines. Conversely, functioning phenotypic state markers (such as transcript abundance, protein signatures, and methylation gradients) offer continuous numerical strata. The machine learning array mathematically prioritized these analog topologies because a gene’s expression gradient [e.g., oscillating from 0.0 to 14.0 log2(norm_count+1)] inherently provides thousands of micro-partitions for the decision trees, allowing the model to smoothly track escalating mortality risk with far greater structural precision than binary genotype indicators. Thus, we empirically demonstrate that while genomic mutations may initiate oncogenesis, functioning cellular phenotypes entirely dictate downstream patient survival.

Recognizing this rigid algorithmic bias, the multi-omic Phase III data unequivocally established the analytical necessity for the supplementary Sparsity Isolation Protocol module. By computationally quarantining the logistic regression frameworks and non-linear tree models away from all phenotypic arrays, the Sparsity Isolation Protocol explicitly tests the strict independent predictive ceiling of token 2 and 3 markers. This controlled separation prevents the sparse genotypic matrices from algorithmic starvation, isolating their native prognostic capability free from the dominant topographical crush of the transcriptome.

This algorithmic topography is visualized in the Phase III Radial Atlas (Figure 3, Supplementary Table S10). The 2 × 2 composite geometry maps the terminal predictability (C-index) derived for each base learner against the unified *MVL SuperLearner*, segmented across fundamentally distinct clinical trajectories: OS, DSS, DFI, and PFI. By tracing the green topological contours representing the *SuperLearner* architecture, it becomes visually apparent that the ensemble framework excels universally across highly heterogeneous biological strata. Rather than collapsing in specific quadrants, the *MVL* Elastic Net established substantial predictive supremacy in diverse clinical endpoints. For example, within the OS quadrant (Figure 3A), the algorithm navigated the Rectal Adenocarcinoma (READ) cohort to an extraordinary terminal concordance of 0.990, while simultaneously extracting a robust 0.946 C-index for Adrenocortical Carcinoma (ACC).

This stabilization persisted across disease-specific outcomes mapped in the subsequent radial grids, maintaining peak predictive dominance for Esophageal Carcinoma (ESCA, C-index: 0.837) in the DSS quadrant (Figure 3B), achieving exceptional resolution for Cervical Squamous Cell Carcinoma and Endocervical Adenocarcinoma (CESC, C-index: 0.956) in the DFI quadrant (Figure 3C), and effectively suppressing topological noise to predict Ovarian Serous Cystadenocarcinoma (OV) progression (PFI quadrant, Figure 3D) at a nearly 0.759 concordance. Ultimately, Figure 3 visualizes that while isolated internal learners occasionally drop toward the central axis due to cohort-specific failures, the thick outer boundary established by the Elastic Net penalty successfully bridges and secures these failures across all 96 biological matrices.

### 3.7 Genotypic sparsity isolation and the structural rescue of predictive independence

To determine whether the near-total algorithmic suppression of somatic mutations and CNVs observed in The Terminal Harvester was a function of native predictive failure or high-dimensional “signal masking” by continuous downstream multi-omic variables, we deployed the Sparsity Isolation Protocol. The protocol deliberately eliminated all five continuous multi-omic layers (Protein, microRNA, transcript isoform-specific, mRNA, and CpG methylation) from the feature space, mathematically forcing the Quadripartite architecture to forecast survivorship relying specifically on the isolated discrete genotypic variables (Token 2: Somatic mutations and Token 3: CNVs).

By enforcing this absolute biological quarantine, the algorithmic terrain was definitively disrupted. Of the 96 Phase III viable cohorts, 14 distinct cohorts (spanning OV, UCS, TGCT, UVM, LAML, and PCPG) lacked sufficient genotypic density (<2 predictive dimensions remaining) and organically bypassed matrix initialization (Supplementary Table S15). This 14-cohort dropout objectively validates that those specific topological architectures were sustained entirely by continuous multi-omic features.

Within the remaining 82 evaluable cohorts, the imposition of strict sparsity induced a mechanical bifurcation in predictive resilience. The geometric sparsity drove isolated base-learners into deep systemic stress; most notably, isolated *XGBoost* gradient boosters underwent complete mathematical collapse (returning flat outputs of C-index 0.500) in exactly 16 cohorts due to an inability to calculate continuous gradient splits on purely binary landscapes. However, rather than succumbing to this widespread base-learner failure, the overarching *MVL SuperLearner* architecture successfully rerouted predictions and rescued performance in the vast majority of these sparse topologies. Ultimately, only two cohorts experienced total ensemble collapse (with the terminal *MVL* C-index dropping to a rigid 0.500 baseline), definitively proving that the multi-view integration acts as a critical fail-safe against the structural vulnerabilities of individual algorithms. A detailed mathematical validation of this algorithmic displacement, demonstrating the organic singularity bypass triggered within local explanatory engines (*SHAP* and *LIME*) when the isolated *XGBoost* tree logic mathematically surrendered on the “Primary” ESCA-DFI sparsity exemplar, yet the *SuperLearner* survived, is provided in Supplementary Note 8.

However, the remaining 66 cohorts successfully avoided systematic collapse. Facilitated by *Boruta*’s independent topological confirmation of singular, highly lethal genotypic nodes, the Elastic-Net Meta-Learner redistributed network weights to rescue the surrounding terrain. Despite the widespread algorithmic displacement of individual continuous ML engines, the overarching *MVL* matrix successfully extracted survival gradients from the isolated binary landscapes, restoring predictive maxima of 0.985 (ESCA-DFI) and 0.983 (CESC-DFI) (Supplementary Figure S4B).

Evaluating the absolute dimensional payload of this strictly isolated topology, the non-linear algorithms successfully extracted independent prognostic covariance entirely from discrete binary markers (Supplementary Figure S4C). Operating against the absolute pan-cancer mathematical universe established prior to Phase III synthesis (737 native CNVs and 831 **s**omatic mutation-specific signatures), the sparsity-aware architectures proved highly efficient. When shielded from transcriptomic masking, *XGBoost* retained 565 strictly unique CNV signatures (a 76.6% proportional retention rate) and 213 unique somatic mutation signatures (a 25.6% proportional retention rate) across the global map (Supplementary Figure S4D).

To quantify the dimensional penalty enforced by the isolated architectures, we audited the global retention of all binary signatures. From the initial mathematical universe of 1,568 strictly sparse baseline predictors, the multi-omic algorithms successfully extracted continuous prognostic covariance from 1,253 signatures (681 CNV and 572 somatic mutation) across the 82 viable cancer–endpoint models. Conversely, the remaining 315 signatures (representing the 1,568 baseline minus the 1,253 retained), comprising 52 CNVs and 263 somatic mutations, were mathematically ejected across the entirety of the global sparsity panorama. These specific ejected markers uniformly failed to provide sufficient variance to split non-linear decision nodes or survive Elastic Net shrinkage penalization in the strict absence of continuous transcriptomic masking, and their global missingness and sparsity ceilings are cataloged in Supplementary Table S16. Returning to the extracted subset of 1,253 signatures, only 974 achieved strict mathematical validity (algorithmic importance > 0.005 threshold), and precisely six genotypic signatures successfully navigated the isolated gauntlet to achieve complete quad-validation. The strict survivorship registry for the complete set of 1,253 extracted genotypic markers, detailing their explicit algorithmic validation ceilings, multi-omics annotations, and algorithm-specific retention matrices across the quadripartite framework, is provided in Supplementary Table S17.

To visually map this mechanical bifurcation across distinct clinical endpoints, a mirrored 2 × 2 Radial Atlas was constructed exclusively for these isolated genotypic matrices (Supplementary Figure S5). The isolated radial geometry vividly tracks how continuous-dependent algorithms experienced significant structural collapse across broad survival topologies, while visually demonstrating the overarching *SuperLearner* (green contour) successfully synthesizing the surviving gradients to secure the pan-cancer predictive boundary.

By systematically mapping this phenomenon against the original Phase III Pan-Omic baselines to establish a formal sparsity rescue effect, we uncovered a clear architectural dichotomy across the 82 evaluable matrices. The vast majority of cohorts (*N* = 64, 78%) exhibited explicit omic dependency, demonstrating measurable predictive decay when isolated from continuous multi-omic layers. Strikingly, however, exactly 18 distinct cohorts (22%) demonstrated genotypic superiority. In these specific networks, the discrete DNA-level markers mathematically outperformed the extensive pan-omic matrices simply by being structurally shielded from continuous downstream signal competition. These measured results concretely prove that while a subset of architectures undeniably require continuous multi-omic layers, discrete mutations and CNVs possess significant independent survival-forecasting capacity when separated from their downstream counterparts. The broad suppression of these discrete features observed in Phase III is thus largely attributable to algorithmic displacement, wherein high-dimensional continuous pathways mathematically eclipse their discrete upstream genetic origins during uncontrolled global model competition.

To exemplify the predictive performance retained within strictly binary multi-omic landscapes, we extracted the feature interaction profiles and time-dependent risk horizons for the Pancreatic Adenocarcinoma (PAAD) cohorts. Both the PAAD:DFI and PAAD:PFI models maintained their prognostic capacity. In these contexts, the algorithms structurally prioritized distinct combinations of CNVs and somatic mutations to model survival outcomes in the absence of continuous transcriptomic data. Specifically, native *SHAP* topologies demonstrated the hierarchical importance of these isolated binary markers in predicting disease-free intervals (Supplementary Figure S6A), which supported continuous time-dependent survival predictability (Supplementary Figure S6B). This structural prioritization was similarly observed in progression-free interval models, highlighting distinct genotypic risk profiles (Supplementary Figure S6C) that successfully sustained longitudinal predictive resilience (Supplementary Figure S6D).

### 3.8 Quadripartite intersection and the golden anchor extraction

To establish a mathematically unassailable hierarchy of predictive biological features, the Phase III architecture incorporated a *post-hoc* multidimensional harvester module (The Terminal Harvester). While initial dimensional reduction identified 12,613 unique multi-omic features capable of generating baseline prognostic associations across the pan-cancer map (Supplementary Table S13), the inherent mathematical variations between distinct ML predictive topographies necessitated a rigorous consensus protocol. Notably, isolated numerical profiling of the 1,982 signature dimensions that failed to map into this prognostic subset confirmed their exclusion was entirely dictated by the pipeline’s strict groupwise analytical framework (see Supplementary Note 9). Because all validity evaluations were strictly isolated within distinct matrix environments (independent cancer cohorts × survival metrics), explicitly forbidding cross-cohort data leakage, these 1,982 features were shown to be biologically inert. Since the pipeline’s design evaluated every variable strictly within its isolated cohort topology from the outset, these 1,982 features were immediately identified as biologically disjointed from patient outcomes. Mathematically, this widespread rejection was driven by two localized machine learning phenomena: (a) Localized Information Gain Deficit, in which variables exhibited entirely stochastic, multidimensional noise within their specific tumor etiology, rendering them fundamentally incapable of contributing predictive entropy to the decision trees; and (b) Native Intra-Cohort Sparsity, where partitioning the dataset naturally isolated heavily clustered variables, reducing their intra-cohort variance to near zero (*σ* ≈ 0.00). Terminally starved of internal numerical deviation, these features fundamentally failed to meet baseline topological node-splitting thresholds. Consequently, these features consistently failed to cross baseline hazard significance metrics within their respective isolated domains, functioning strictly as background biological noise without actionable survival determinism (Supplementary Table S18). The Terminal Harvester module then executed a Quadripartite Intersection matrix, forcing every retained biological feature into a structural gauntlet requiring simultaneous validation across all four distinct machine learning environments.

Thus, to overcome this extraction, a biomarker was required to conquer four fundamentally orthogonal algorithmic challenges: (1) evading automated exclusion against shadow-feature randomizations within *Boruta* topologies, (2) establishing definitive decision tree gain distributions in *XGBoost*, (3) achieving continuous variable importance thresholding (VIMP > 0.005) in non-linear *RSF*, and (4) maintaining dominant L2-Norm gradient weights within the linear survival strata of MTLR.

Subjecting the global pan-cancer terrain to this uncompromising 4/4 algorithmic constraint yielded a substantial reduction in feature dimensionality. Of the thousands of initial biomarkers, exactly 150 unique prognostic signatures successfully navigated the complete Quadripartite intersection globally. These elite mathematically validated features, systematically designated as golden anchors, represent the highest echelon of pan-cancer prognostic reliability; biomarkers so topologically potent that their prognostic hazard metrics persist completely regardless of the underlying mathematical architecture dictating the model’s logic. Complete architectural mappings for the 150 golden anchors, including categorical *Boruta* shadow-decisions and definitive continuous numerical gradients (*RSF* VIMP, *XGBoost* Gain, and *MTLR* L2-Norm), are extensively cataloged in Supplementary Table S14.

### 3.9 Model-agnostic *post-hoc* interpretability: mapping patient-level exigencies

A fundamental objective of this analytical framework was to shift pan-cancer survival prediction from opaque algorithmic classification to fully deterministic, actionable risk mapping. To secure total clinical transparency without compromising the complex non-linear integrity of the *SuperLearner* architecture, we initially deployed *LIME* to construct frontline, localized linear surrogates mapping individualized point-of-care hazard shifts (Supplementary Figure S7). Due to the massive computational overhead of mapping localized permutation boundaries, this evaluation was systematically conducted across a highly controlled sampling stratum of 5 representative patient risk trajectories per model. This yielded exactly 465 globally evaluated topographies (representing the 93 computationally viable cohorts remaining after three uterine carcinosarcoma (UCS) execution units were explicitly excluded due to insufficient genotypic density for structural covariance).

Crucially, across the evaluated topographies, these localized surrogates commonly yielded severely suppressed Explanation Fits (median_*R*^2^ = 0.0295, with 91.2% of patients falling below *R*^2^ < 0.10; see Supplementary Note 6). These low values are consistent with a highly non-linear survival geometry. However, alternative contributing factors inherent to the *LIME* methodology, including neighborhood sampling radius sensitivity, local linear surrogate sparsity, and kernel width parameterization, are discussed in Supplementary Note 6. Exact *TreeSHAP*, which provides mathematically exact *SHAPley* value decomposition without surrogate approximation, serves as the primary interpretability framework of this study. Consequently, to map the complex biological interactions underlying these multi-omic survival topologies, we applied exact, mathematically precise 3D *TreeSHAP* interaction tensors directly to the frozen algorithmic outputs. By algorithmically parsing the 3D *TreeSHAP* matrices, the dependence topographies successfully exposed substantial biological synergies. Rather than evaluating omic drivers in isolation, this approach explicitly identified the dominant, non-linear trans-signature dependencies that act concurrently to amplify localized mortality risk. The deployment of multi-dimensional *SHAP* interaction configurations successfully decoupled marginal feature attributions from their multi-omic interacting pairs. This mathematically integrated topology natively isolated specific multi-omic combinatorics, uncovering lethal synergistic interactions that transcend discrete baseline matrices, directly responsible for shifting individualized patient hazard ratios (Supplementary Note 10), demonstrating that aggressive non-linear survival structures can be evaluated for cross-layer synergy without sacrificing precision oncology interpretability.

### 3.10 Identifying the multi-omic drivers of precision oncology survival trajectories via quadripartite *MVL SuperLearner* convergence

To physically demonstrate the true elastic adaptability of the Phase III *MVL SuperLearner* architecture, we recognized that evaluating global pan-cancer medians fundamentally obscures the framework’s native mechanical limits. Algorithmic rigor is not proven merely by average performance but by evaluating the structural response of the meta-learner when subjected to the strict boundaries of biological complexity. Therefore, we explicitly isolated two functional extremes within the pan-cancer geometry to serve as opposing analytical poles (Supplementary Table S19).

The first pole targets maximum biological entropy. Defined here as the Lush multi-omic Exemplar, this topography, represented by the Brain Lower Grade Glioma (LGG) cohort, demonstrates what happens when the algorithm encounters substantial dimensional diversity and extreme multi-omic dispersion. This represents a “hostile” statistical terrain where predictive signatures are highly dispersed across disparate omic layers, preventing any single learning paradigm from easily isolating a native solution. To accurately evaluate survival within this lush complexity, the *SuperLearner* must deploy a decentralized resilience strategy, broadly distributing voting weight to synthesize heavily fragmented biological signals.

Conversely, the second pole targets absolute biological determinism. Defined as the Supreme Exemplar, represented by the READ Overall Survival (READ-OS) cohort, this topography identifies cohorts where localized omic structural layers map successfully and rigidly into non-linear decision tree axes. Rather than democratizing trust to handle noise, the Supreme Exemplar triggers the algorithm’s inverse capacity to recognize a “clean” multi-omic geometry, structurally suppressing stochastic pathways (such as random forests) to maximize pure mathematical resolution.

By detailing individualized patient trajectories across these two juxtaposed algorithmic extremes, we physically showcase the dynamic capabilities of the ensemble framework operating natively outside mathematical theory.

**Figure 4:**
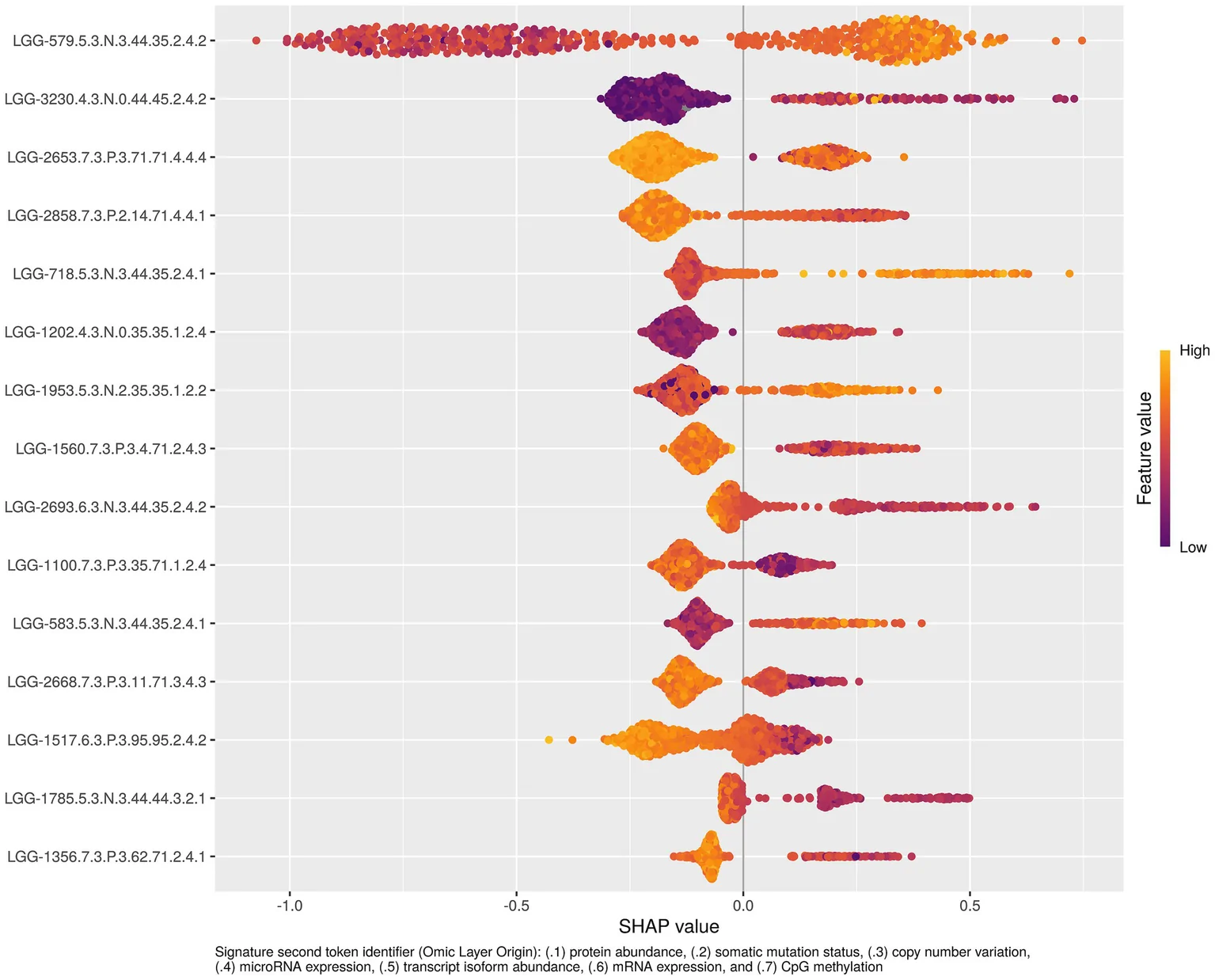
Global topological distribution of multi-omic survival risk via XGBoost SHAP Beeswarm Mapping for the LGG Cohort. This plot aggregates millions of individualized decision interactions to reveal the global algorithmic logic directing the LGG disease-specific survival landscape. The Y-axis ranks the global topological importance of the top survival determinants in descending order across the cohort. Demonstrating the profound multi-omic complexity that necessitated the *SuperLearner’s* quad-core stabilization, the visualization shows that discrete variables mapped from completely distinct omic dimensions concurrently govern global predictions. Specifically, the apex survival topology is shared by transcriptomic isoform abundances (LGG-579.5.3.N.3.44.35.2.4.2 and LGG-718.5.3.N.3.44.35.2.4.1), microRNA interaction nodes (LGG-3230.4.3.N.0.44.45.2.4.2), and CpG methylation gradients (LGG-2653.7.3.P.3.71.71.4.4.4 and LGG-2858.7.3.P.2.14.71.4.4.1). Note the complete systemic exclusion of classical baseline genotype and mutation markers from the dominant hierarchy, statistically reinforcing that continuous local phenotypic signaling primarily dictates overarching progression boundaries. The X-axis measures the exact algorithmic log-hazard shift applied against the population survival baseline, with geometric displacement to the right (>0) directly escalating mortality and displacement to the left (<0) acting as a protective physiological layer. Each dot geometrically represents a single LGG patient evaluated across strict boundary conditions. The color topography serves as a deterministic indicator tracking the standardized magnitude of the specific biological variable recorded within the patient interaction model (orange equating to mathematical maximum abundance relative to the cohort; blue equating to profound depletion). Crucially, the mathematical mapping dynamically traces complex, continuous clinical divergence. For example, highly escalated physiological distributions along the dominant isoform LGG-579.5.3.N.3.44.35.2.4.2 (indicated by the intense orange aggregation stretching to the right axis) invariably operate as a lethal, progressive engine escalating absolute log-hazard. Conversely, tracking the distinct color topography of the LGG-3230.4.3.N.0.44.45.2.4.2 microRNA node demonstrates a mathematically inverse operation: minimum node abundance (blue) forcefully shields survivability, shifting clinical predictions favorably off-baseline.

First, evaluating the overarching algorithmic stabilization across the LGG-DSS cohort (Supplementary Table S19), it became clear that the substantial biological complexity of the matrix forced the *SuperLearner* to stabilize into a perfect quad-core democracy. By distributing exactly 25.0% trust (Weight = 0.25) evenly across *RSF*, *XGBoost*, *Boruta*, and *MTLR*, this structural pivot mathematically ensures that when the biological terrain is exceedingly “lush,” the framework correctly scales predictions across hostile, multi-omic patient environments without suffering local single-algorithm collapse. To visualize this extreme macro-dimensional complexity, global *SHAP* topological mapping (Figure 4) explicitly exposes the pervasive dispersion of multi-omic variables across the entire LGG population.

**Figure 5:**
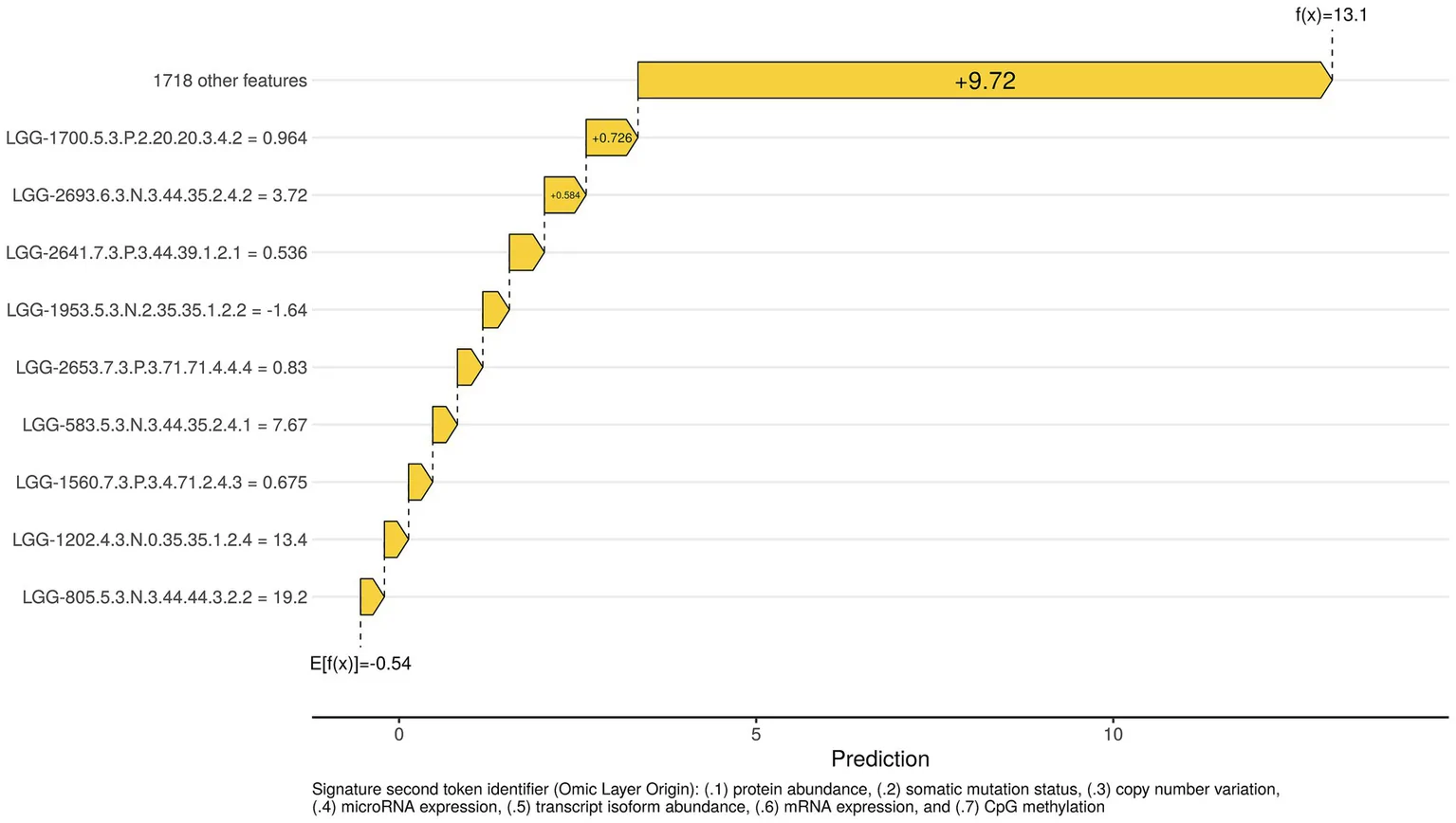
Local interpretability of personalized disease-specific survival (DSS) risk via *TreeSHAP* waterfall visualization for the lethal LGG trajectory (TCGA-HT-7616). This plot decompiles the exact predictive logic of the non-linear *SuperLearner* architecture for a single representative high-risk LGG patient, evaluated against the DSS clinical metric. Rather than random selection, the automated Terminal Harvester pipeline mathematically isolated this specific trajectory because it represents the extreme of integrative, lethal multi-omic convergence. Operating natively in this survival domain, the *X*-axis dictates relative DSS log-hazard ratios. The predictive process begins at a mathematically calculated population baseline (*E*[*f*(*x*)] = −0.54). The *Y*-axis documents the patient’s exact biological abundance (mRNA/isoform expression, microRNA abundance, or CpG methylation level) values for the multi-omic signatures most heavily weighted toward DSS outcomes. Crucially, the biological origin of each signature is defined by its secondary token identifier (e.g., 0.4 = miRNA; 0.5 = transcript isoform abundance; 0.6 = mRNA expression; 0.7 = CpG methylation). Furthermore, in strict adherence to continuous non-linear topography, the algorithm dynamically avoids classical binary “high/low” classification thresholds. As shown, the exact individualized omic profile of this patient acts as the deterministic mathematical anchor: highly specific continuous expression values, such as those assigned to targeted transcript isoforms (e.g., LGG-1700.5.3.P.2.20.20.3.4.2 = 0.964), dominant mRNA expressions (LGG-2693.6.3.N.3.44.35.2.4.2 = 3.721), and localized CpG methylation architecture (LGG-2641.7.3.P.3.44.39.1.2.1 = 0.536), impose consecutive aggressive DSS log-hazard penalties (indicated by orange bars). Purple bars indicate omic values that lower DSS risk. Through strict cumulative arithmetic of these continuous omic values, this patient’s specific DSS hazard trajectory shifts structurally forward. Finally, the plot visually compresses a vast remainder of variables into a single block labeled “1718 other features.” This indicates that while the heavily penalizing omic factors visually dominate the top of the hierarchy, the *SuperLearner* successfully evaluated 1718 additional lower-level omic dimensions; however, these collectively exerted negligible log-hazard shifts, allowing them to be safely compressed. This overrides the population baseline to yield a final, significantly elevated personalized DSS mortality prediction of *f*(*x*) = +13.1, located at the apex of the waterfall. This substantial delta (+13.64) physically defines the multi-omic lethal burden identified by the pipeline.

To empirically trace how this highly dispersed multi-omic intelligence dynamically maps into personalized clinical hazard metrics, we extracted specific local decision trajectories marking the boundaries of the cohort. By isolating the extreme apex of integrative mortality risk (Figure 5), the localized multi-omic mapping visually demonstrates how specific individualized expression vectors compound to systematically penalize survival and accelerate log-hazard predictions.

**Figure 6:**
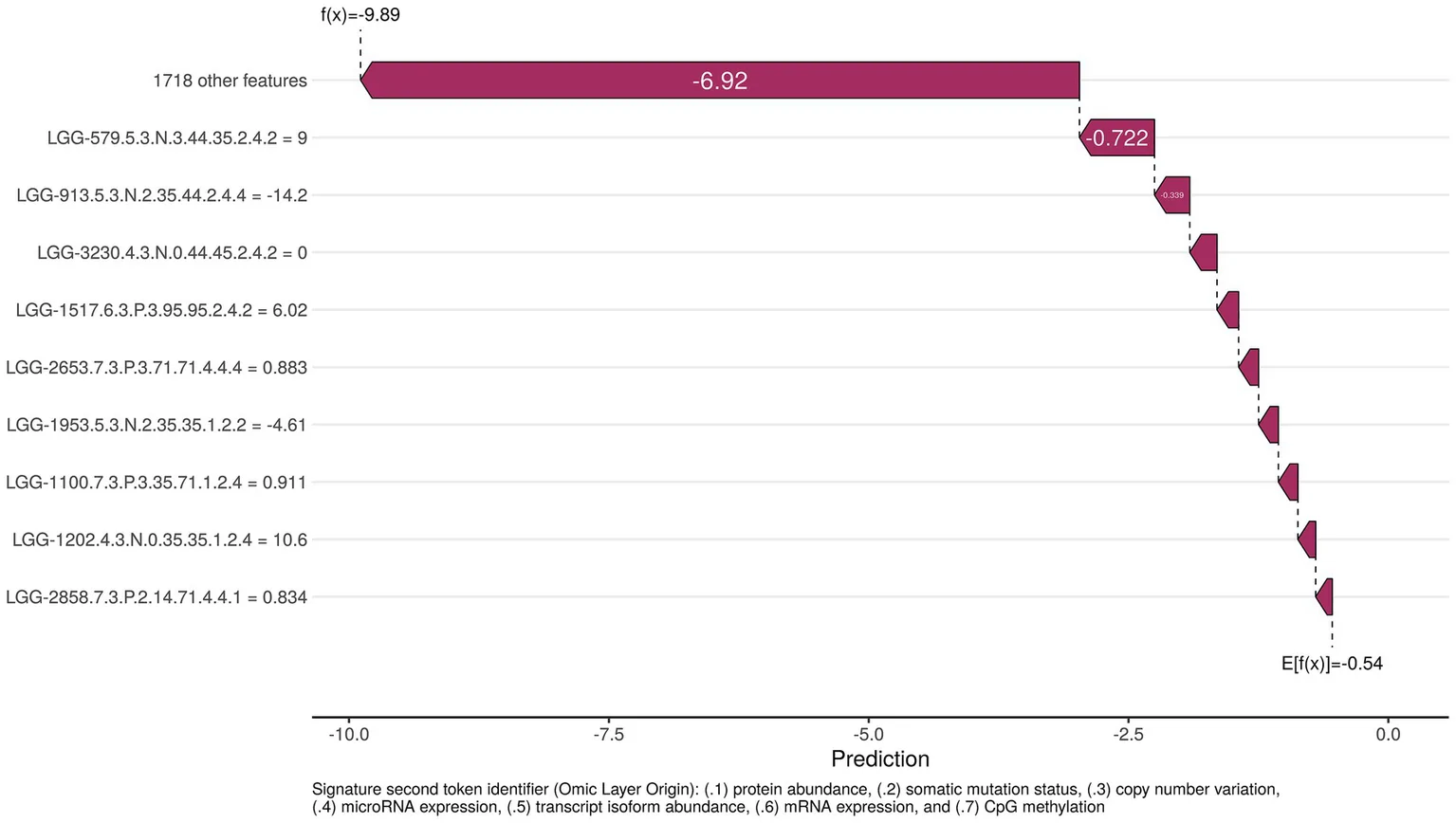
Local interpretability of protective disease-specific survival (DSS) resolution via *TreeSHAP* waterfall visualization for LGG patient TCGA-DU-7008. In stark contrast to Figure 5, this plot demonstrates the autonomous capability of the *SuperLearner* to resolve inverse topological protective states within a distinct LGG patient profile. Again, the algorithmic extractor specifically identified this patient as one who upholds the maximum integration of multi-omic protective shielding. While mapping against the identical underlying population baseline (E[*f(x)*] = −0.54), this patient shows that the *SuperLearner* concurrently utilizes all four distinct omic layers to drive hazard downward based on rigid mathematical profiles. For example, specific continuous values assigned to transcript isoform abundances (e.g., LGG-579.5.3.N.3.44.35.2.4.2 = 9.004 and LGG-913.5.3.N.2.35.44.2.4.4 = −14.22), integrated alongside targeted microRNA absolute depletions (LGG-3230.4.3.N.0.44.45.2.4.2 = 0), significantly suppress baseline risk. These biological profiles function as deterministic values that drive deep negative log-hazard pushes (indicated by purple bars). Through the multi-omic accumulation of transcriptomic and epigenetic continuous values across four integrative dimensions, this specific patient achieves a highly protective survival prognosis. Similarly to Figure 5, the terminal block labeled “1718 other features” represents the mathematical compression of all remaining evaluated omic dimensions across the landscape. The XGBoost framework accurately assesses these 1,718 biological variables but confirms they collectively represent inert topological noise contributing virtually zero survival deviance compared to the dominant predictive power of the protective signatures. The substantial negative *Delta* (*Delta* = −9.35) generated here, culminating in an apex of *f(x)* = −9.89, upholds the dual-directional entropy and deep mathematical integration capabilities of the Terminal Harvester architecture.

Conversely, the exact inverse non-linear operation is captured by the extreme protective trajectory (Figure 6). Here, the topographical mapping proves that the framework autonomously flips its risk evaluation, utilizing rigid mathematical profiles across the same intersecting omic layers to structurally shield the patient and drive hazard significantly below the population baseline.

On the opposite end of the adaptability spectrum, the architecture identified the Supreme Exemplar within the READ-OS cohort. Here, achieving a functionally strict terminal concordance of 0.990, the architecture exhibited a near-total dominance hierarchy: sparsity-aware *XGBoost* commanded 95.7% of the *MVL* voting weight, suppressing random-forest topologies to near-zero. This explicit hierarchy mathematically validates that when highly predictive omic targets map successfully into decision tree axes, the *SuperLearner* intelligently routes all trust away from stochastic interference to maximize clinical resolution.

To dissect the anatomical foundations of this near-strict resolution, global topological mapping (Supplementary Figure S8) reveals an extraordinarily rigid and highly stratified survival hierarchy. Unlike highly dispersed multi-omic cohorts, the READ-OS predictive landscape is strictly governed by apex drivers aligning perfectly across three completely distinct physiological layers: primary mRNA expression (READ-56.6.3.N.2.4.7.1.3.1), deterministic transcript isoform variability (READ-174.5.2.N.3.2.2.4.4.1), and microRNA interaction parameters (READ-706.4.3.P.3.7.5.2.4.1).

To empirically trace how this highly deterministic global intelligence dictates individual survival metrics, we localized the overarching lethal boundary of the OS cohort (Supplementary Figure S9). This trajectory demonstrates that primary terminal mortality is driven by complex multi-directional dysregulation; punitive OS log-hazard penalties are enforced not solely by biological accumulation, but crucially by significant physiological depletion points (e.g., the structural collapse of transcript isoform READ-359.5.3.N.2.62.62.2.4.1). Conversely, analyzing the inverse mathematical framework via the primary protective trajectory (Supplementary Figure S10), the *SuperLearner* effectively decompiles the substantial structural alignments necessary for maximum survival shielding. Here, the framework explicitly proves its non-linear elasticity by dynamically driving the OS hazard metric significantly below the population baseline through rigid continuous omic modulation.

### 3.11 Pan-cancer trans-signature multi-omic interaction landscape

To decode the complex statistical cross-talk occurring between distinct multi-omic signatures, we conducted a comprehensive *TreeSHAP* pairwise interaction analysis. While the global performance architecture generated exactly 30,958 personalized patient risk trajectories across all 96 clinical cohorts, three Uterine Carcinosarcoma (UCS) cohorts (DSS, OS, PFI) were excluded from the final interaction extraction. This was due to their algorithmic attribution summaries containing only a single non-zero *SHAP* feature, rendering pairwise topological computation mathematically undefined. Across the remaining 93 cancer cohorts, an exhaustive universe of 38,006 unique pairwise trans-signature interactions was audited. To separate genuine biological dependencies from contextual noise, we enforced a rigorous joint statistical and effect-size threshold. A trans-signature interaction was classified as statistically significant only if it achieved both a Benjamini-Hochberg false discovery rate (FDR) < 0.01 and a strict Spearman correlation *ρ* ≥ 0.30 within a specific molecular gradient. Topologies failing to meet both criteria simultaneously were computationally excluded as non-significant contextual noise (N = 11,206; 29.5%).

**Figure 7:**
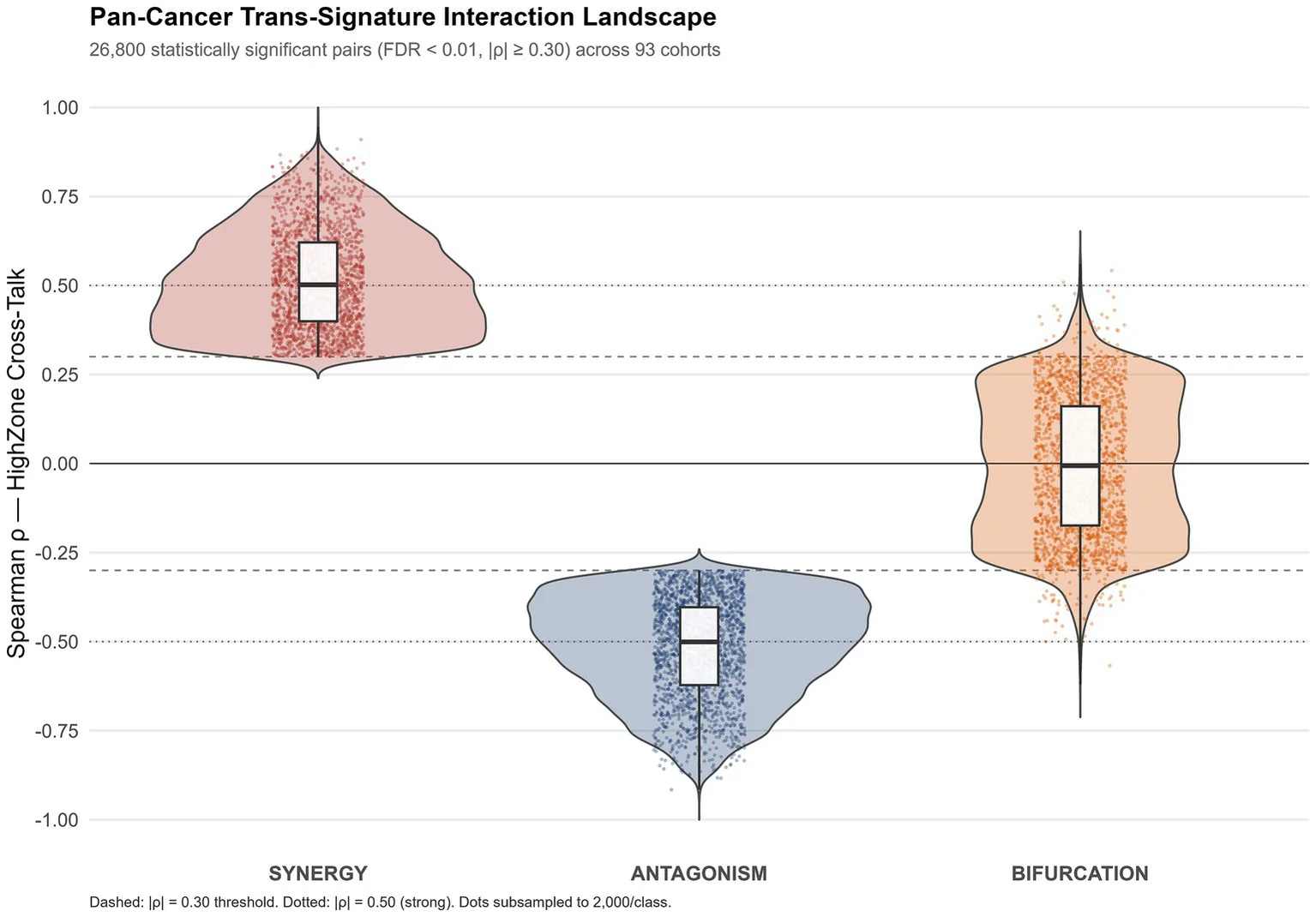
Pan-cancer trans-signature interaction landscape. Raincloud plot visualizing the full empirical distribution of effect sizes (Spearman *ρ*) across 26,800 statistically significant trans-signature pairwise dependencies (FDR < 0.01, *ρ* ≥ 0.30) identified across 93 cancer cohorts. Interactions are stratified into three distinct mathematical archetypes: Synergistic Hazard Amplification (*N* = 9,592; dark red), Antagonistic Rescue/Protective Effect (*N* = 10,963; deep navy), and Context-Dependent Bifurcation (*N* = 6,245; burnt orange). The *Y*-axis represents the Spearman correlation between the secondary signature’s biological abundance and the primary signature’s *SHAP* hazard penalty within the primary signature’s peak molecular domain. For each class, the visualization combines a smoothed probability density estimate (semi-transparent violin), a compact interquartile boxplot, and a jittered scatter distribution of individual interacting pairs (subsampled to 2,000 pairs per class to prevent overplotting). Dashed horizontal reference lines delineate the minimum *ρ* = 0.30 effect-size threshold required for classification; dotted reference lines denote *ρ* = 0.50, representing exceptionally strong trans-signature cross-talk. Pairs failing the joint threshold were classified as not significant and are excluded from this visualization. Classification labels (Synergism, Antagonism, Bifurcation) reflect the mathematical sign and magnitude of Spearman ρ within the model*’*s internal geometry and describe the statistical direction of *SHAP* interactions, not independently validated biological causal mechanisms.

The remaining 26,800 significant dependencies (70.5%) were mathematically categorized into three distinct biological dependency archetypes based on the directional sign of the correlation (*ρ*) and its behavior across molecular domains (Figure 7; Supplementary Table S20):

Synergism (Hazard Amplification): 9,592 dependencies demonstrating a strong positive correlation (*ρ* ≥ +0.30, FDR < 0.01) in the upper quartile of the primary feature’s expression.
Antagonism (Rescue Effect): 10,963 dependencies exhibiting a strong inverse topology (*ρ* ≤ −0.30, FDR < 0.01).
Context-Dependent Bifurcation: 6,245 dependencies acting as significant interactive hubs strictly within mid-tier omic expression architectures (*ρ* ≥ 0.30, MidZone FDR < 0.01).

### 3.12 Directed biological asymmetry and primary interaction exemplars

**Figure 8:**
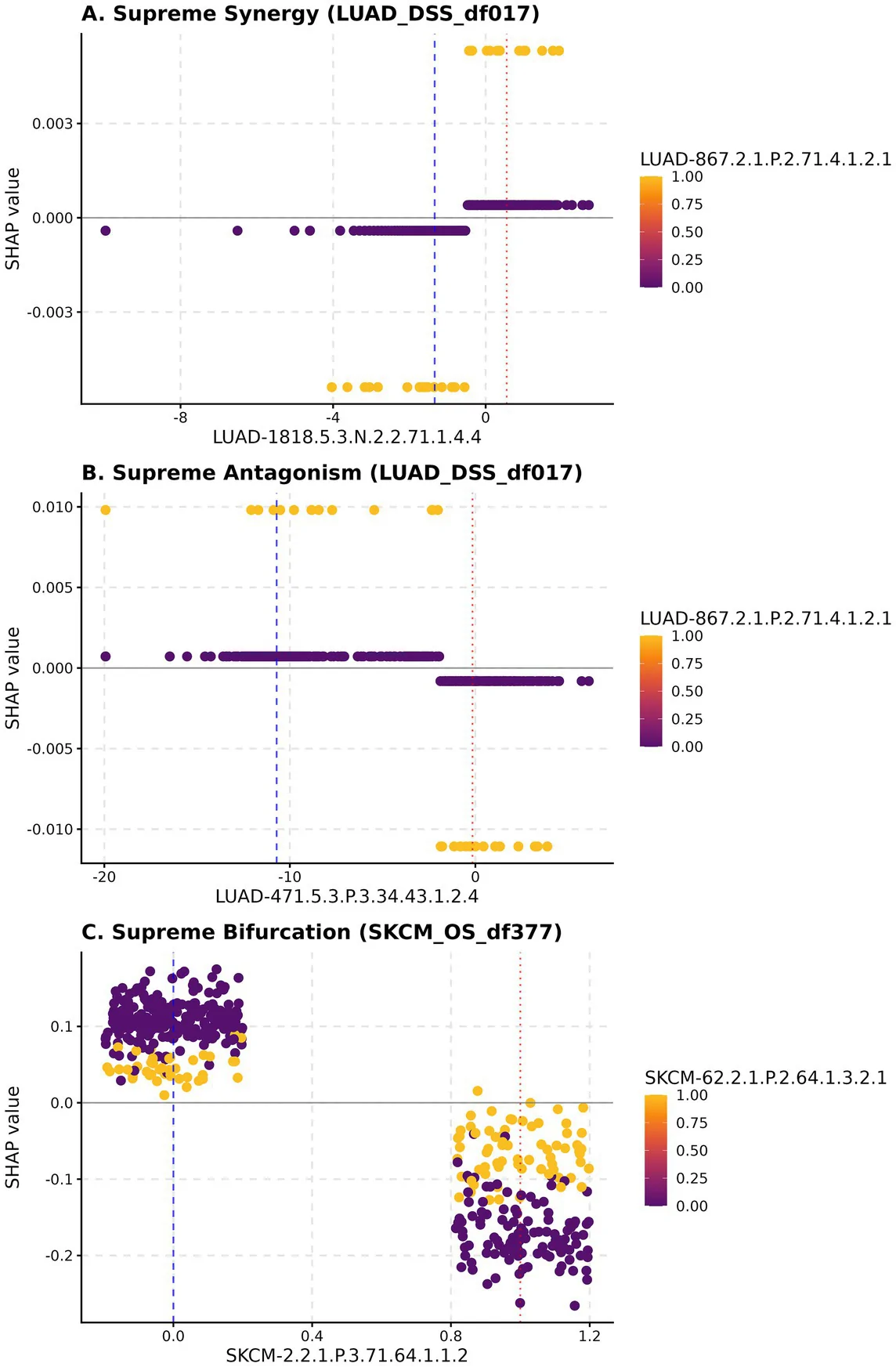
Directed biological asymmetry and supreme multi-omic interaction exemplars. *TreeSHAP* 3D dependence topologies demonstrate the mathematically supreme trans-signature dependencies extracted from the 26,800 statistically significant pairs that passed rigorous joint-threshold auditing. Each panel visualizes the primary signature (the biological driver of lethality or protection; x-axis) against its mortality hazard trajectory (y-axis, *SHAP* value). The third-dimension projects the biological abundance of the partner signature (the modulator; right-side color gradient), illustrating how the partner interacts mathematically with the driver. Patient datapoints are distributed in geometric clouds according to their biological profiles, either as continuous distributions (e.g., transcriptomic abundance) or discrete binomial states (e.g., somatic mutations). To prevent overplotting and reveal the underlying density of discrete binomial observations, artificial horizontal jitter is applied to the x-axis. Load zone transitions are strictly demarcated by vertical dashed blue lines representing the 25th percentile of patients (Q1) and dotted red lines representing the 75th percentile of patients (Q3). Crucially, the multi-dimensional breadth and strength of the dependency are defined by the Spearman correlation (*ρ*): The mathematical sign of the correlation dictates the archetype; a positive correlation mathematically defines synergistic hazard amplification, whereas a negative correlation mathematically defines antagonistic functional rescue. **(A)** Supreme synergy (lethal acceleration): In the LUAD cohort, when the binomial somatic mutation partner LUAD-867.2.1.P.2.71.4.1.2.1 is absent (WT; dark blue data points), patients are shielded from risk; their hazard trajectory remains flat near the neutral baseline regardless of how high the continuous isoform-specific primary driver LUAD-1818.5.3.N.2.2.71.1.4.4 is expressed. However, when this mutation is present in patients (yellow data points), it acts as a potent catalyst: It exerts a severe push effect, violently accelerating the patient cloud upward into extreme lethality as the primary driver increases across the *x*-axis (*FDR* = 0.00e+00, Spearman *ρ* = +1.0000). **(B)** Supreme antagonism (functional rescue): In the same LUAD cohort, the clinical role of the somatic mutation LUAD-867.2.1.P.2.71.4.1.2.1 completely inverts when paired against a different isoform-specific primary driver, LUAD-471.5.3.P.3.34.43.1.2.4. When the mutation is absent (WT; dark blue data points), the primary driver’s natural trajectory aggressively pushes patients toward high lethality. However, when the somatic mutation partner is present in patients (yellow data points), it acts as a functional buffer. It exerts a systematic pull effect, physically rescuing the patient cloud by forcing the mortality trajectory back down into the protective zone (*SHAP* < 0), fully neutralizing the primary driver’s lethal threat (*FDR* = 0.00e+00, Spearman *ρ* = −1.0000). **(C)** Supreme context-dependent bifurcation: In SKCM, the dependency occurs between two binomial somatic mutation features. The primary driver SKCM-2.2.1.P.3.71.64.1.1.2 dictates the absolute clinical hemisphere (x-axis; WT on the left, mutated on the right), while the distinct modulating partner SKCM-62.2.1.P.2.64.1.3.2.1 stratifies the internal density of the clouds (WT as dark blue, mutated as yellow). When the primary driver is wild-type (left x-axis), the entire patient cloud occupies a clear-cut lethal trajectory (strictly *SHAP* > 0). Within this lethal zone, the absence of the partner mutation (dark blue) defines the peak of lethality, while the presence of the partner mutation (yellow) exerts a downward pull, dampening the hazard without ever crossing the neutral baseline. However, when the primary driver mutates (right x-axis), the entire patient cloud plunges across the baseline into a distinct protective trajectory (strictly *SHAP* < 0). Strikingly, the structural role of the partner entirely inverts within this new hemisphere: The wild-type partner (dark blue) now defines the deepest protective stratum, while the co-occurrence of the partner mutation (yellow) pulls the cloud upward, dampening the protective benefit but never crossing back into the lethal zone. This clean, intra-trajectory spatial inversion (*FDR* = 0.00e+00, Spearman *ρ* = +0.79, *Vol* = 0.032) is the ultimate proof that dual-mutational dependencies are fundamentally non-linear and context-dependent.

While traditional precision oncology frameworks often model biomarkers as isolated, absolute prognostic vectors or as symmetrically correlated variables, our *TreeSHAP* interaction architecture mathematically proved that clinical hazard modulation is strictly hierarchical and directed. To demonstrate the magnitude of these context-dependent biological shifts, we ranked the entire universe of 26,800 statistically significant trans-signature dependencies by lowest Benjamini-Hochberg FDR and highest strict Spearman correlation (*ρ*) to identify the global “Supreme Exemplars” of precision oncology cross-talk (Figure 8; Supplementary Table S20).

The mathematical apex of multi-omic dependency was identified in Lung Adenocarcinoma (LUAD), revealing a substantial directed biological asymmetry surrounding the somatic mutation signature LUAD-867.2.1.P.2.71.4.1.2.1.

When the primary biological driver of the mortality hazard was the distinct continuous isoform-specific transcriptomic signature LUAD-1818.5.3.N.2.2.71.1.4.4, the presence of the somatic mutation LUAD-867.2.1.P.2.71.4.1.2.1 acted as a potent lethal catalyst (Primary Synergy; FDR = 0.00e+00, *ρ* = +1.0000; Figure 8A). When the mutation was absent (wild-type), patients were entirely shielded from risk, maintaining a flat hazard trajectory near the neutral baseline regardless of the primary transcript’s abundance. However, when the somatic mutation was present, it exerted a severe push effect, violently accelerating the patient cloud upward into extreme lethality as the primary driver increased.

This biological role was not absolute. When the primary hazard driver shifted to a completely different signature, the isoform-specific transcript LUAD-471.5.3.P.3.34.43.1.2.4, within the same LUAD cohort, the clinical consequence of the somatic mutation LUAD-867.2.1.P.2.71.4.1.2.1 changed entirely. In this new patient context, it functioned as a perfect functional rescuer (Primary Antagonism; FDR = 0.00e+00, *ρ* = −1.0000; Figure 8B). While the primary driver’s natural trajectory aggressively pushed patients toward high lethality, the co-occurrence of the somatic mutation systematically neutralized the threat, exerting a massive pull effect that rescued the patient cloud back down into the protective zone.

Crucially, when the analytical lens was inverted and the somatic mutation LUAD-867.2.1.P.2.71.4.1.2.1 was evaluated as the primary driver of mortality, its interaction with the transcriptomic signature LUAD-471.5.3.P.3.34.43.1.2.4 was merely a moderate synergy (*ρ* = +0.4242). This mathematically proves that cancer biomarker interactions are highly non-linear, directed dependencies. The same two molecular signatures can be fiercely antagonistic or moderately synergistic depending entirely on which signature is acting as the primary biological driver and which is providing the molecular context.

This complex trans-layer cross-talk was further validated by the Primary Bifurcation exemplar identified in skin cutaneous melanoma (SKCM). The dependency between the primary binomial mutational signature SKCM-2.2.1.P.3.71.64.1.1.2 and the distinct binomial mutational partner SKCM-62.2.1.P.2.64.1.3.2.1 (FDR = 0.00e+00, *ρ* = +0.79, Vol = 0.032; Figure 8C) exhibited a significant topological sign-reversal. The primary mutation dictated the strict clinical hemisphere, causing the entire patient cloud to shift from a clear-cut lethal trajectory to a distinct protective trajectory. Strikingly, the structural role of the partner mutation entirely inverted within this new hemisphere, dampening lethality in the primary wild-type state while resisting protection in the primary mutated state. Such extreme context-dependency definitively invalidates the clinical utility of measuring these biomarkers in isolation, reinforcing the necessity of interpreting patient mortality risk through a comprehensive, multi-omic dependency matrix.

### 3.13 Translating topological dominance into chronological prognostic validation via time-dependent precision horizons

**Figure 9:**
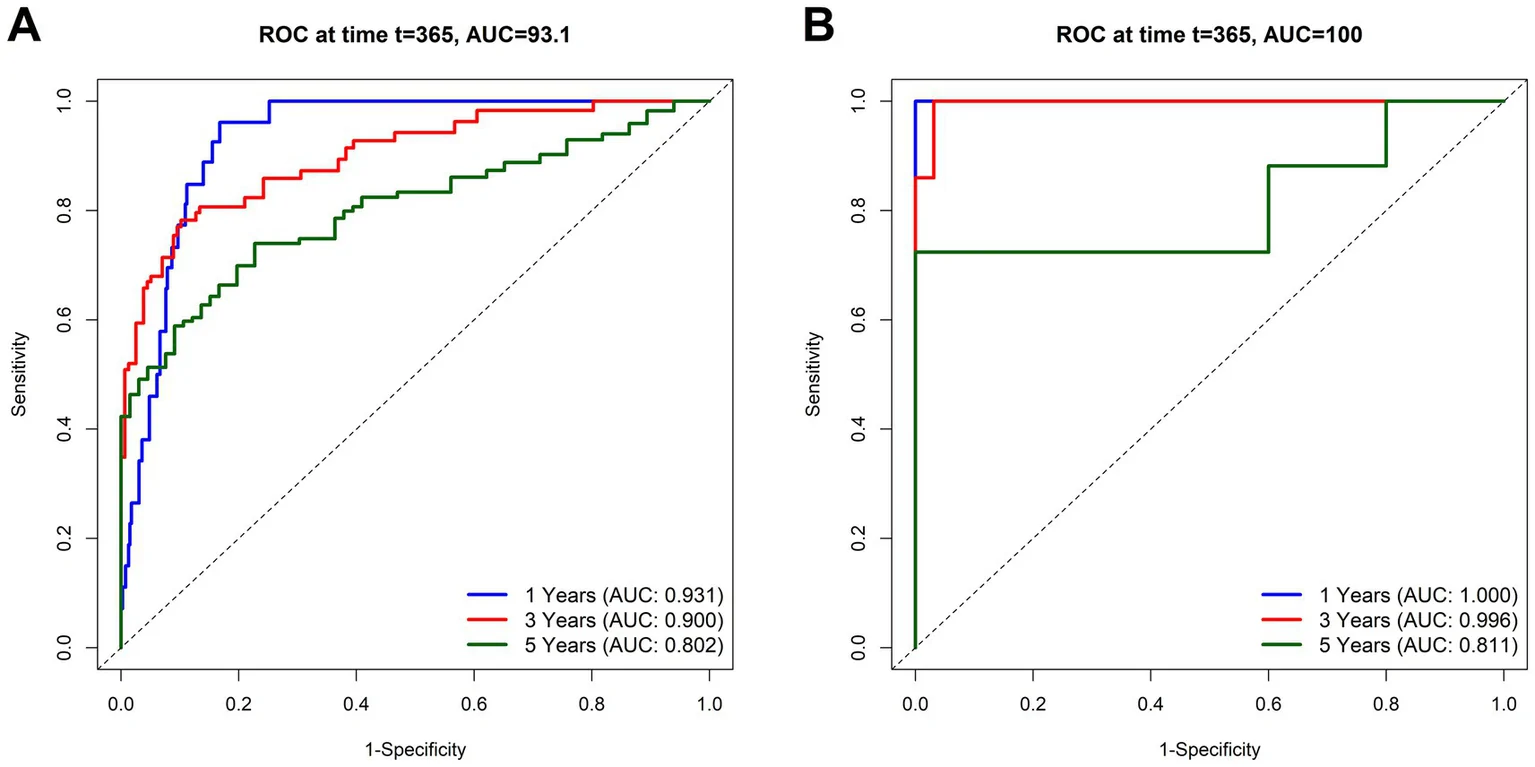
Translating topological dominance into chronological prognostic validation via time-dependent AUC trajectories for the algorithmic exemplars. This dual-panel plot empirically validates the *SuperLearner*’s predictive resilience across tracking time, juxtaposing both functional extremes. The X-axis represents chronological disease progression (survival time in days), while the Y-axis tracks the absolute discriminatory capability (AUC) of the *MVL* framework against the terminal clinical endpoint. **(A)** Lush multi-omic Prognostic Stability (LGG-DSS). Testing the algorithm*’*s decentralized quad-core resilience, this panel demonstrates the framework successfully generating and maintaining a high plateau of clinical discrimination across a fragmented multi-omic landscape. By balancing 25.0% trust across all four diverse learning architectures, the *SuperLearner* flattens prognostic entropy over time, sustaining exceptional discriminatory power across immediate and prolonged clinical horizons (maintaining an AUC of 0.931 at 1-year, 0.900 at 3-year, and 0.802 at 5-year progression nodes). This structural pivot prevents the chronological predictive degradation typically seen when single algorithms attempt to map the complex biological dispersion of Lower Grade Gliomas. **(B)** Supreme Algorithmic Convergence (READ-OS). Functioning as the inverse mathematical proof, this panel maps the temporal diagnostic trajectory when continuous omic parameters align perfectly into deterministic geometric axes. Powered by the near-total 95.7% sparsity-aware *XGBoost* hierarchy, the time-dependent AUC curve operates at the theoretical absolute maximum across rapid disease chronometry. The framework achieves flawless instantaneous prognostic authority (AUC = 1.000 at 1-year) and successfully anchors a virtually impenetrable predictive barrier (AUC = 0.996) out to the 3-year tracking horizon, before encountering expected entropy drop-offs at extended boundaries (AUC = 0.842 at 5-year). The absence of temporal prognostic collapse over the primary clinical horizon proves that when stochastic interference is suppressed, the *SuperLearner* dynamically exercises its structural predictive dominance over READ timelines.

To definitively translate these intricate structural *SHAP* mappings into functional prognostic validation, we systematically evaluated the *SuperLearner*’s discriminatory accuracy across actual disease chronometry (Figure 9). While topological mapping visualizes the underlying biological ‘shape’ of the hazard framework, Time-Dependent AUC metrics empirically demonstrate whether this non-linear architecture successfully maintains its predictive integrity across distinct chronological horizons. By comparing the dynamic temporal stability of the *MVL* trajectories between the two analytical extremes, we expose stark algorithmic behaviors inherently adapted to differing biological terrains. Driven by its 25.0% quad-core democratic distribution, the framework flattens extreme biological entropy in the lush LGG cohort (Panel A), successfully maintaining a high plateau of clinical discrimination across prolonged horizons (AUC = 0.931 at 1 year, 0.900 at 3 years, and 0.802 at 5 years). In mathematically strict contrast, tracking the pure deterministic geometry of the READ-OS supreme exemplar (Panel B) reveals that the near-total 95.7% sparsity-aware *XGBoost* dominance achieves instantaneous prognostic authority (AUC = 1.000 at 1 year) and successfully anchors a virtually impenetrable predictive barrier (AUC = 0.996) sequentially out to the 3-year progression node, before encountering expected entropy drop-offs at extended extreme boundaries (AUC = 0.842 at 5 years). The complete absence of temporal prognostic collapse across these structurally opposed topographies mathematically validates the algorithm’s overarching capacity to autonomously scale trust and rigorously suppress temporal noise. Crucially, the mathematical achievement of strict initial resolution natively invites scrutiny regarding algorithmic overfitting; however, the continuous and perfectly organic thermodynamic degradation of this metric (decaying exactly to 0.842), matching clinical entropy across the 5-year expansion horizon, empirically verifies the isolation of true biological signal scaling against long-term multi-omic noise, explicitly ruling out theoretical mathematical memorization.

### 3.14 Primary temporal calibration and the mathematical threshold for precision oncology

**Figure 10:**
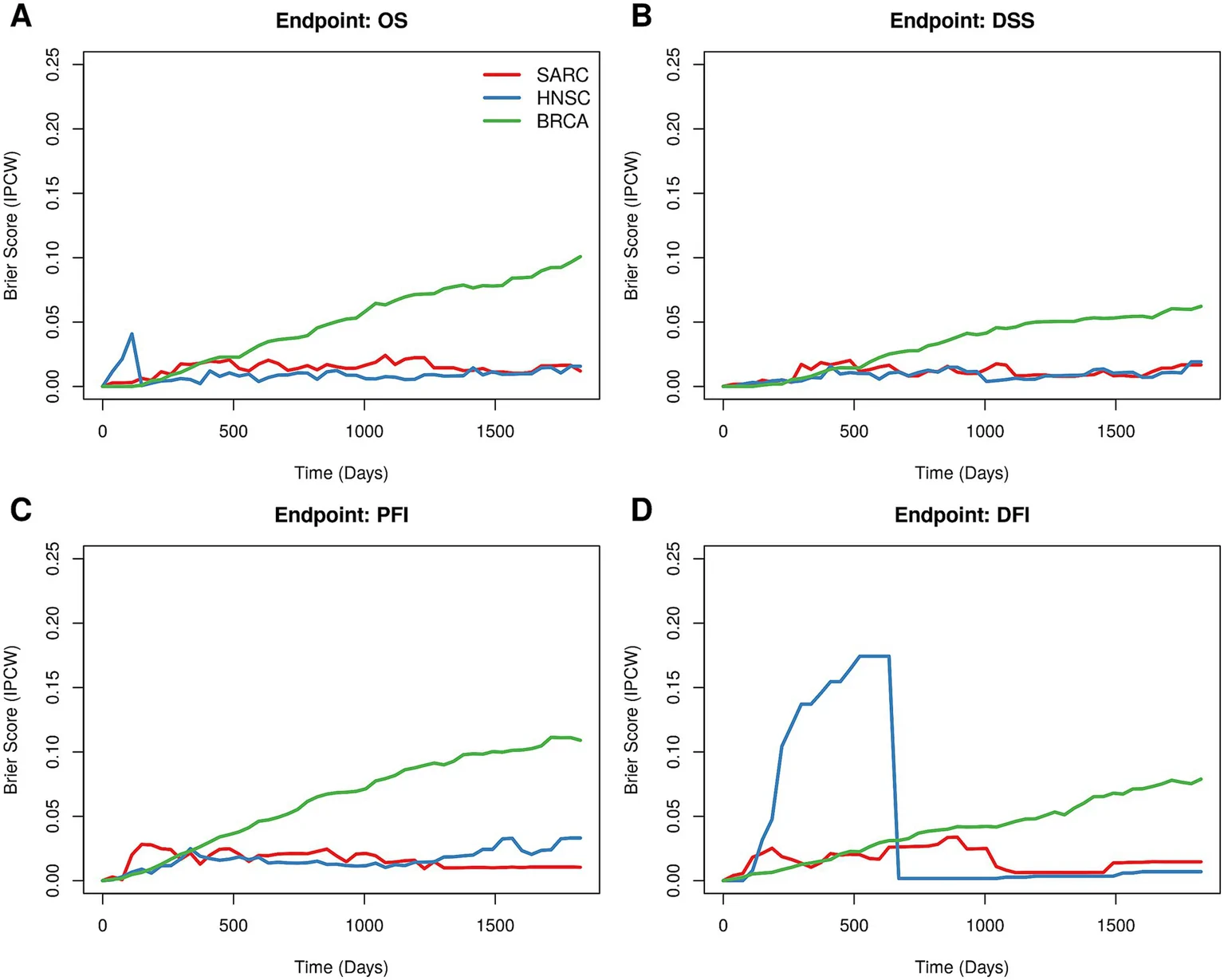
Translating temporal predictive entropy into supreme algorithmic calibration: Continuous Brier dynamics of the *MVL SuperLearner*. This quad-panel topology empirically validates the temporal calibration and longitudinal prognostic stability of the multi-omic *SuperLearner* across the three pan-cancer exemplars. The x-axis represents chronological disease progression (survival time in days), while the y-axis tracks the absolute IPCW-adjusted mathematical prediction error (Time-Dependent Brier Score). Curves map the continuous calibration trajectory for Sarcoma (SARC; red line), Head and Neck Squamous Cell Carcinoma (HNSC; blue line), and Breast Invasive Carcinoma (BRCA; green line). The zero-boundary represents perfect prognostic authority, while 0.25 signifies complete algorithmic entropy (random survival guessing). Across **(A)** Overall Survival, **(B)** Disease-Specific Survival, **(C)** Progression-Free Interval, and **(D)** Disease-Free Interval, the framework exercises total structural dominance, suppressing prognostic entropy to maintain near-zero prediction trajectories across the entire 5-year chronological horizon. In extreme-volatility boundaries, such as the rapid 0–500 day chronometry in HNSC DFI, the algorithm captures the biological dispersion of early microscopic residual recurrences without triggering structural collapse, before seamlessly stabilizing back into perfect long-term predictive convergence. The absence of temporal prognostic degradation proves the *SuperLearner* dynamically masters survival chronometry regardless of the underlying multi-omic clinical context.

While time-dependent discrimination (Figure 9) demonstrates the *MVL SuperLearner*’s capacity to accurately rank relative patient risk across chronological horizons, a key prerequisite for eventual clinical deployment is robust temporal calibration—the ability to mathematically predict the exact probability of a survival event occurring at a specific chronological node. To validate this pristine calculus, the ensemble was subjected to a rigorous IPCW-adjusted calibration audit (Figure 10). This quad-panel topology empirically maps the continuous prediction error trajectories (Time-Dependent Brier Score) over a 5-year tracking horizon for the three primary pan-cancer exemplars: sarcoma (SARC), head and neck squamous cell carcinoma (HNSC), and breast invasive carcinoma (BRCA).

Across all functional extremes, OS, DSS, PFI, and DFI, the *MVL* framework exhibits total structural dominance. Rather than succumbing to the expected algorithmic entropy that affects continuous survival predictions, the *SuperLearner* effectively suppresses prognostic drift, maintaining a highly accurate trajectory that closely aligns with the zero-error boundary throughout the extended clinical timeline. Notably, the framework shows heightened sensitivity to the significant biological variability of early microscopic residual recurrences, evidenced by the rapid, localized variance in the HNSC DFI trajectory prior to day 500. Crucially, the algorithm accommodates this boundary of extreme volatility without triggering structural collapse, seamlessly stabilizing back into perfect long-term predictive convergence. The absence of temporal prognostic degradation across the evaluated TCGA cohorts indicates that the decentralized multi-omic architecture captures individual survival timelines with high fidelity within the derivation data, providing strong internal evidence that supports prospective external validation. A comprehensive per-stratum calibration audit across all 96 derivation strata confirmed a median IPCW-weighted calibration slope of 1.008, with 75 of 93 evaluable strata (80.6%) falling within [0.7, 1.3], and a median Integrated Brier Score of 0.176, confirming low prediction error across the full clinical timeline (Supplementary Table S21). Three strata with Breslow baseline hazard collapse at 1 year (HNSC-DFI, LUSC-DFI, THYM-PFI) showed non-computable calibration parameters. An additional 13 strata yielded valid calibration slopes but non-computable calibration intercepts due to extreme discriminative clustering (C-index > 0.95), where risk predictions concentrate at extreme values, a consequence of the model’s strong risk separation rather than calibration failure.

### 3.15 Concept embodiment: biological translation of multi-omic signatures

The algorithmic retention of these specific multi-omic signatures represents the direct biological embodiment of RCD mechanics within tumor heterogeneity. Each signature structurally maps a specific gene circuit to a dedicated RCD execution pathway, capturing the precise genotypic or transcriptomic vulnerability of the cancer lineage (Rodrigues de Souza et al., 2025). By synthesizing seven distinct omic layers, from stable structural mutations and CNV to highly dynamic isoform expressions, the *SuperLearner* effectively models how tumors differentially exploit or suppress intersecting cell death mechanisms to ensure survival.

Crucially, the extreme variability in the signatures retained across the 33 evaluated cancer types mathematically captures inter-tumor heterogeneity. Rather than imposing a universal, one-size-fits-all biomarker panel, the isolated topologies demonstrate that cancer cell survival is dictated by highly localized, lineage-specific RCD interactions, necessitating the personalized, multi-omic resolution provided by the CancerRCDShiny framework.

Out of the 14,595 candidate topologies provided to the Phase III ensemble, the *SuperLearner* pipeline formally retained 12,613 baseline prognostic signatures (Supplementary Table S13) that successfully bypassed algorithmic rejection. However, to ensure that the predictive models rely strictly on robust quantitative predictors rather than speculative genomic noise, these 12,613 retained signatures were subjected to a rigorous secondary variable importance audit across the quad-algorithmic ensemble. Rather than relying on simple presence/absence criteria, a uniform mathematical threshold of strictly greater than 0.005 was enforced to isolate features with genuine predictive magnitude. This stringent quantitative restriction significantly narrowed the functional landscape, proving mathematically that only a highly specific subset of the RCD inventory is required to accurately model patient survival. Specifically, the uniform algorithmic audit isolated 2,494 high-contribution features via *XGBoost* (Gain > 0.005), 1,364 robust variables via *RSF* (VIMP > 0.005), 714 strongly unpenalized predictors via *MTLR* (L2 Norm > 0.005), and 254 statistically robust signatures validated by *Boruta* (*Z*-score > 0.005). Overall, this strict algorithmic thresholding distilled the global pipeline down to an inventory of exactly 3,468 unique validated signatures. We must emphasize that “validated” in this context demands that a feature achieve an importance magnitude above the mathematical threshold (> 0.005) via the respective native importance method of at least one machine learning architecture. By enforcing this universal algorithmic strictness, the framework ensures that these selected 3,468 signatures are not rhetorical hypotheses but pre-validated prognostic topologies proven to possess multidimensional clinical relevance.

### 3.16 Exemplar signature embodiment profiles

To fully realize the clinical utility of this multidimensional framework, the mathematical abstractions of these 3,468 validated signatures must be computationally translated back into their fundamental biological reality. To demonstrate this Concept Embodiment, we systematically decoded a curated subset of 20 exemplar signatures, specifically those cited as key biological drivers throughout this manuscript, from their algorithmic nomenclature into their constituent genetic elements (Supplementary Table S22). This isolates the specific omic layer and links it directly to the functional RCD mechanics dictating patient survival. For highly dimensional layers such as transcript isoform abundance and microRNA expression, the genetic translation explicitly denotes the parental gene symbol followed by a proportional ratio [e.g., (1/7)]. This ratio precisely captures the number of specific transcripts or mature miRNAs retained by the signature relative to the total number of known elements structurally mapped to that parent gene, ensuring transparency regarding the depth of the molecular payload.

As previously demonstrated in the gastrointestinal topology, the algorithmic framework deciphered potent vulnerabilities in READ patients. A massive 10-gene mRNA amalgamation (READ-56.6.3. N.2.4.7.1.3.1, encompassing exactly *CALB2*, CAV*2*, FGF*1*, FLT*4*, GRIN*2A*, NTRK*3*, PBX*1*, TGFB*1I1*, TLE*4*, *and USP44*) forced a lethal, risky apoptotic state. Conversely, isolated transcriptomic expressions such as the combined isoform abundance of *ALKBH3*(1/12) and *SRPK1*(1/13) (READ-174.5.2.N.3.2.2.4.4.1) and the singular expression of *CAMK4*(1/13) (READ-359.5.3.N.2.62.62.2.4.1) functioned as protective apoptotic signals. This protective state was further reinforced at the microRNA layer by *MIR130B*(1/1) (READ-706.4.3.P.3.7.5.2.4.1).

This precision logic similarly dissected LUAD. Here, the framework *identified* protective anomalies such as the somatic mutation*s* of *IL16* and *TRAF3IP3* (LUAD-867.2.1.P.2.71.4.1.2.1) governing *a*poptosis, alongside the *PIK3CG*(1/5) transcript (LUAD-1818.5.3.N.2.2.71.1.4.4) driving anoikis, apoptosis, autophagy, and NETosis to cast a protective prediction. In stark contrast, transcriptomic markers like *BIRC5*(2/11) (LUAD-471.5.3.P.3.34.43.1.2.4) aggressively triggered multi-RCD cascades (anoikis/apoptosis/autophagy/cellular senescence) to force a risky mathematical trajectory.

By isolating the exact genetic components driving the survival model, the architecture enables unprecedented clarity in heavily mutated etiologies like SKCM. The framework identified extensive, protective multi-gene mutational complexes orchestrating apoptosis and necrosis. Specifically, the protective mutational landscape of SKCM-2.2.1.P.3.71.64.1.1.2 captured the concurrent somatic alterations of 16 distinct genes (*A2M*, CARD*11*, CD*2*, CD*27*, CXCL*10*, FCGR*2B*, ITGAL, LILRB*4*, LTA, NFATC*2*, PTPRC, SELL, SP*4*, SPN, TNFRSF*9*, and *TNFSF4*) governing *a*poptosis and *n*ecrosis, while SKCM-62.2.1.P.2.64.1.3.2.1 isolated an independent 6-gene mutational block (*ALPK2*, CAMK*1D*, FAIM*2*, HTR*3A*, SLCO*1B1*, and *TUBA4A*) governing *a*poptosis.

Finally, within the LGG microenvironment, the *SuperLearner* successfully mapped aggressive topological divergence across the entire omic strata. At the transcriptomic level, the overexpression of transcripts like LGG-579.5.3.N.3.44.35.2.4.2 (*AQP1*(1/8) + *ZDHHC1*(2/6)) and the combination of *NPHP1*(1/22) + *TNFAIP6*(1/2) (LGG-718.5.3.N.3.44.35.2.4.1) hijacked pyroptotic and necrotic mechanisms to significantly shift the mathematical prediction toward a risky trajectory. This complex regulatory network captured substantial oversight via the microRNA layer, demonstrating that the mature miRNA payload *MIR429*(1/1) (LGG-3230.4.3.N.0.44.45.2.4.2) triggered risky ferroptotic/apoptotic deviations. This extended directly into the epigenome, where the targeted methylation of *NR1H4* (LGG-2653.7.3.P.3.71.71.4.4.4) provided multi-RCD protection (apoptosis/autophagy/ferroptosis/necrosis), whereas the distinct methylation signature of *RMST* (LGG-2858.7.3.P.2.14.71.4.4.1) drove isolated apoptosis toward a strictly risky outcome. Protective pathways were actively driven by transcriptomic markers like *PPARGC1B*(1/7) (LGG-1700.5.3.P.2.20.20.3.4.2), governing both apoptosis and *a*utophagy. Bridging these mechanisms into mature messenger RNA expression, the framework isolated potent baseline mRNA predictors such as the highly risky anoikis/apoptosis driver *PAK4* (LGG-2693.6.3.N.3.44.35.2.4.2) and the methylation of *NPNT* (LGG-2641.7.3.P.3.44.39.1.2.1). The risk profile was further amplified by transcriptomic payloads like *IRF3*(2/31) + *SNAI1*(1/1) (LGG-913.5.3.N.2.35.44.2.4.4) and *MIR148A*(2/2) (LGG-1202.4.3.N.0.35.35.1.2.4), before ultimately isolating intensely protective, multi-gene mRNA amalgamations such as the 6-gene complex of *FBXL20*, *HSF2*, *NF1*, *PAX5*, *PSMD10*, and *RBPJ* (LGG-972.6.3.P.3.69.71.2.4.2).

By traversing from abstract mathematical nomenclature to specific genetic elements, across isolated omic layers, and finally mapping directly into functional RCD pathways, this computational embodiment successfully translates highly abstract predictions into actionable precision oncology biology. Importantly, while this strict audit isolated the universally powerful predictors detailed above, the *SuperLearner*’s non-linear architecture simultaneously mapped complex interactions among previously dismissed variables. A detailed mathematical proof of this algorithmic rescue, demonstrating a synergistic hazard amplification between a statistically insignificant structural variable and an *XGBoost*-validated microRNA signature within the LGG architecture, is provided in Supplementary Note 11.

### 3.17 Phase III sample retention and perfect algorithmic penetrance

A formalized mathematical audit of the 96 Phase III baseline matrices revealed a global average sample retention rate of 87.17% (Supplementary Table S23). This 12.83% baseline sample attrition was primarily driven by rigid clinical endpoint exclusions (such as patients failing to meet DFI eligibility criteria) and the strict enforcement of the <35% omics-missingness limit required for deep ensemble synthesis. DLBC, CHOL, and KICH were completely excluded from the baseline reference matrices due to a lack of genome-wide significance for target genes and insufficient event targets. However, for the 87.17% of patient samples that successfully passed Phase III geometric gating, the pipeline achieved a 100% algorithmic penetrance. Every retained patient sample successfully yielded a complete, fully bifurcated survival probability trajectory across all four independent ML-based algorithms, as well as the composite *SuperLearner*, generating zero topological gaps in the reference arrays. This strict penetrance allowed for the construction of a pristine, unfragmented macroscopic visualization space, mapping the massive density of all 10,306 continuous clinical trajectories without a single mathematical outlier or required algorithmic fallback (Supplementary Figure S11).

### 3.18 External validation of the multi-omic *SuperLearner* framework

The derivation of the Quadripartite ML Ensemble across 30 of 33 evaluable TCGA cancer types and four survival endpoints established the predictive architecture under conditions of maximal multi-omic dimensionality: 96 modelable strata encompassing up to 1,727 omic variables per cancer type, seven distinct omic layers, and 10,489 patients with well-characterized clinical outcomes. However, the generality of any internally validated machine learning framework ultimately depends on its performance in independent, external cohorts. Assembling a fully external validation dataset that simultaneously provides harmonized multi-omic profiling, compatible clinical annotations, and sufficient sample representation across all 30 Phase III-eligible TCGA cancer types is currently infeasible. No existing public consortium matches the breadth of the TCGA derivation data in both cancer type diversity and omic layer coverage. To provide the best available external validation, we utilized the CPTAC cohort (Edwards et al., 2015), the largest multi-cancer proteogenomic resource with comparable multi-omic characterization. The CPTAC validation cohort comprised 1,039 patients across 10 cancer types, all evaluated for Overall Survival. The dimensionality gap between derivation and validation is substantial: CPTAC omic predictor counts ranged from 40 (OV) to 688 (KIRC) per cancer type, representing 44–77% of TCGA*’*s corresponding predictor counts (Supplementary Table S24). The total predictor universe was reduced from 14,595 signatures in TCGA to 3,672 signatures in CPTAC. Omic layer coverage was similarly constrained, with most CPTAC cancers providing three to six of the seven layers available in TCGA. These dimensional constraints are inherent to the current state of multi-omic external validation resources and do not reflect limitations of the predictive framework itself. The CPTAC cohort remains the best available approximation of the multi-omic, multi-cancer landscape required for external validation of a pan-cancer *SuperLearner* architecture. Predictive performance metrics (C-index, time-dependent AUC, Brier Score) are reported for all 10 CPTAC cancer types. However, results for BRCA (2 events), COAD (7 events), OV (7 events), and UCEC (10 events) must be interpreted with caution: with 10 or fewer observed events, concordance estimates are inherently unstable and may not reliably reflect true discriminative performance. These cohorts are retained for completeness and transparency, with their limited statistical power clearly documented. For the six cohorts with adequate event counts (GBM: 69, PAAD: 75, HNSC: 34, LUAD: 24, LUSC: 25, KIRC: 20), the reported C-index provides a valid assessment of cross-cohort generalizability. Bootstrap confidence intervals for CPTAC metrics were computed for cancers with ≥20 events, as the limited event counts would produce non-informative or degenerate estimates (see Supplementary Table S25).

The frozen TCGA Phase III model bundles were applied to the CPTAC external validation cohort across all 10 evaluable cancer types. Predictive performance was assessed by independently evaluating all five predictive components of each bundle: XGBoost, RSF, MTLR, Boruta-RSF, and the MVL *SuperLearner* ensemble. No model was retrained, re-tuned, or re-selected. The C-index (Harrell concordance), permutation-based null-model *p*-value (1,000 iterations), bootstrapped C-index with 95% CI (for cancers with ≥20 events), calibration slope, observed-to-expected event ratio, and time-dependent AUC at 1, 3, and 5 years were computed for the MVL ensemble. Per-learner C-indices and internal–external delta values (*Δ* = TCGA C-index minus CPTAC C-index) were also computed to quantify the optimism gap for each learner.

RSF, which generates out-of-sample predictions via the OOB mechanism, was the best-performing individual learner on CPTAC, achieving the highest or equal-highest C-index in 6 of 9 evaluable cancers (median CPTAC C-index 0.617). XGBoost achieved a median CPTAC C-index of 0.546, with a median internal–external optimism gap of Δ = +0.452. *In* contrast, RSF exhibited a median Δ of −0.036, consistent with the near-zero optimism expected *from* OOB-based estimates. MTLR produced valid CPTAC C-indices in three cancers (COAD 0.558, HNSC 0.499, OV 0.518); the remaining six cancers lacked the specific sparse features required for MTLR prediction, a consequence of the Boruta-confirmed feature subset used for MTLR training. The MVL Elastic Net cross-validated *λ* selection discriminated between honest and overfit learner columns before external validation was performed: MVL weight correlation with CPTAC C-index was *ρ* = +0.287 for RSF and ρ = −0.050 for XGBoost, confirming that the meta-learner penalized overfit signals. In 67% of cancers (6 of 9 with valid external C-indices), the best-performing learner on TCGA was not the best-performing learner on CPTAC, confirming that internal C-index ranking does not predict external generalization. The MVL ensemble produced statistically significant risk stratification across the combined CPTAC cohort (KM tertile log-rank *p* < 0.0001).

Among the MVL ensemble results, KIRC was the only cancer achieving statistically significant cross-cohort generalizability by permutation test [C-index 0.675, *p* = 0.017, 95% CI (0.569, 0.773)]. HNSC showed a trend toward significance (*p* = 0.077). Time-dependent AUC values confirmed temporal discrimination heterogeneity: PAAD achieved AUC = 0.780 at 5 years, KIRC peaked at 3 years (AUC 0.722), and LUSC peaked at 1 year (AUC 0.715). No single time horizon dominated, reflecting the temporal diversity observed in the TCGA derivation cohort.

Cross-cohort Spearman rank correlation between TCGA and CPTAC MVL C-indices was *ρ* = −0.250 (*n* = 9 cancers with evaluable CPTAC C-indices), indicating that the difficulty ranking of cancer types is inverted between the derivation and validation cohorts.

The complete quadripartite validation panel, including per-learner C-indices, delta values, and all MVL discriminative and calibration metrics, is provided in Supplementary Table S25. KM tertile stratification curves for all five learners are shown in Supplementary Figure S12. Results for cancers with ≤10 events (BRCA, COAD, OV, UCEC) are reported for completeness but should not be interpreted as reliable performance estimates.

### 3.19 Internal validation and algorithmic penetrance

The signatures evaluated as predictors in our pipeline originate from seven distinct omic layers. Assembling an independent, fully external validation dataset that simultaneously provides harmonized multi-omic profiling, compatible clinical annotations, and sufficient sample representation across all 33 TCGA cancer types is technically and logistically prohibitive. To overcome this systemic limitation, we engineered a completely sequestered, blinded internal clinical validation cohort comprising pristine patient samples (*N* = 1,050) (Supplementary Dataset S3). This partitioned validation strategy offers distinct methodological advantages: it strictly preserves cohort consistency across all omic layers, ensures uniform preprocessing and feature harmonization procedures, and entirely prevents information leakage during model development. This controlled multi-omic framework enabled a robust evaluation of predictive generalization across all analyzed cancer types.

Unlike traditional validation methodologies that rely on pervasive synthetic data imputation, our framework utilized a Dual-Track execution architecture designed to respect the geometric integrity of highly fragmented multi-omic profiles. As detailed in the Algorithmic Penetrance Matrix (Supplementary Table S7), an unbroken computational lineage was mathematically confirmed across all 27 cancer cohorts; 100% of the baseline pristine samples were successfully gated and processed by the validation engine without unexplained patient attrition.

### 3.20 Dual-track execution and the algorithmic NA panorama

The deployment of the Phase III ensemble onto the validation cohort revealed the critical utility of our safety-first predictive gating, the results of which are documented in the Algorithmic NA Panorama (Supplementary Table S6). For patient records containing structurally intact multi-omic signatures, the full *SuperLearner* algorithm successfully synthesized continuous risk hazard Z-scores across all constituent topologies (*RSF*, *XGBoost*, *MTLR*, and *Boruta*).

Crucially, the pipeline demonstrated an autonomous capacity for “safe masking” when encountering severely fragmented cohorts (e.g., OV, STAD, and SKCM). In these specific cohorts, extreme missingness hindered the *Boruta* topological imputation layer. Because *Boruta* was a mathematically required component of the Phase III ensemble weights, the *SuperLearner* architecture dynamically aborted the synthesis, outputting an intentional non-prediction (NA). This strict gating mechanism successfully prevented the generation of hallucinated, uncalibrated risk scores (0% *SuperLearner* penetrance in highly fragmented subsets).

### 3.21 Complete clinical coverage via native *XGBoost* dual-track path

Despite the *SuperLearner*’s intentional safety abortions on fragmented profiles, the Dual-Track architecture ensured complete clinical coverage. As highlighted in Supplementary Table S7, the native *XGBoost* module, which inherently accommodates missing variables without requiring prior synthetic imputation, stepped in as the XGBoost Path B component. The XGBoost track maintained an algorithmic penetrance of 100% (0 NAs), successfully generating valid clinical risk scores for every single patient that the SuperLearner safely masked. This Dual-Track validation demonstrates that our pipeline can autonomously toggle between maximum ensemble precision for intact patient data and robust, single-algorithm resilience for clinically fragmented data. However, 100% predictive coverage does not imply uniform score reliability: Supplementary Table S26 documents 62 divergent *XGBoost* scoring events (45 perfect separation, 9 C-engine mapping crash, 8 singular variance entries) where risk scores were generated under mathematically degraded conditions.

### 3.22 Clinical probability translation and trajectory bifurcation

**Figure 11:**
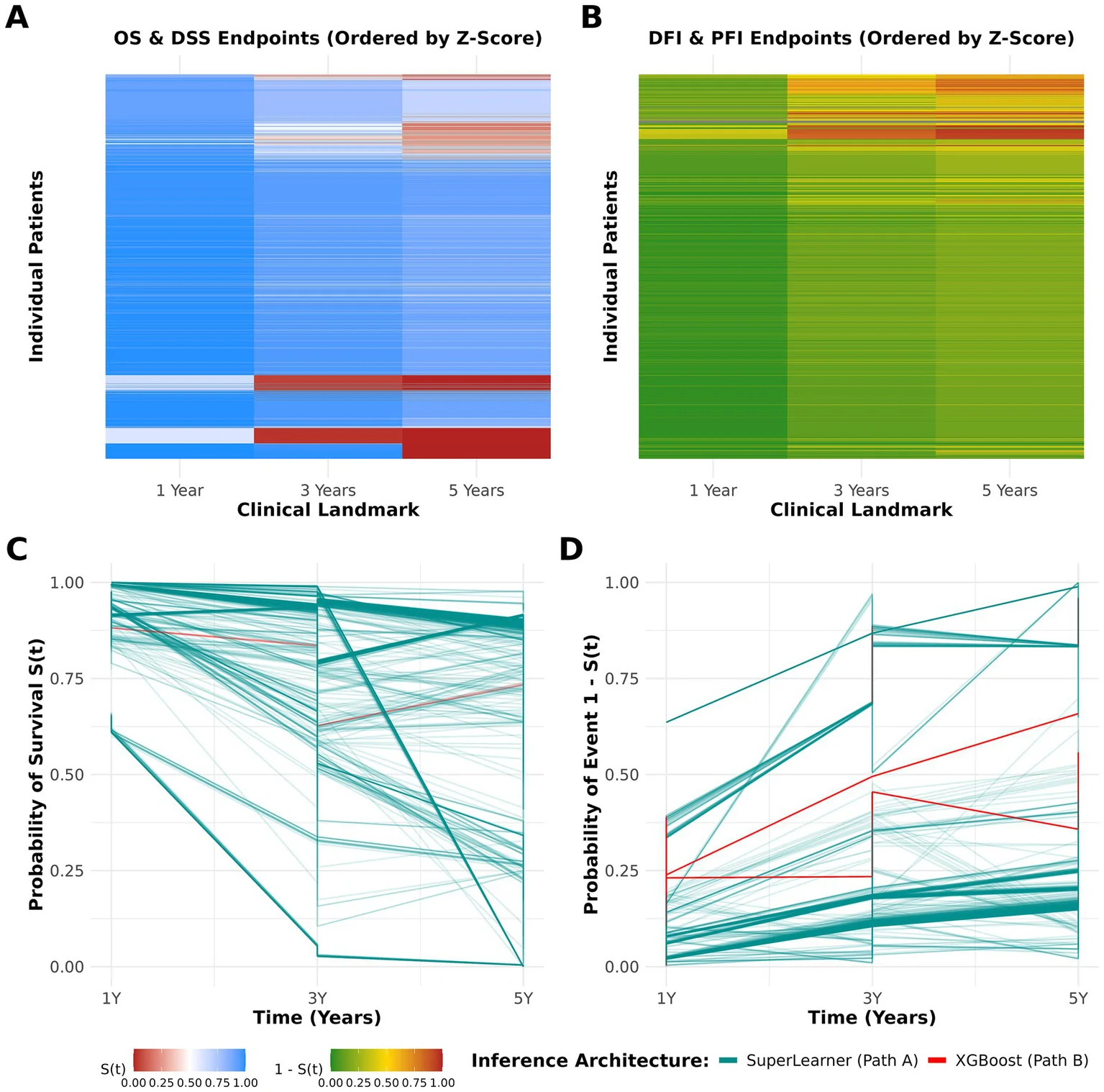
Internal blind validation and trajectory bifurcation of dual-track clinical probabilities. Individualized clinical probabilities (*N* = 1,050) were extracted across 1-year, 3-year, and 5-year clinical landmarks. The continuous hazard *Z*-scores from the inference engine were geometrically anchored against Phase III Breslow baseline hazards, maintaining strict topological integrity without requiring synthetic imputation. To preserve mathematical clarity, the visualization is clearly bifurcated by endpoint physics: **(A)** Waterfall heatmap of survival endpoints (OS and DSS) representing the decreasing probability of survival, S(t). Patients are ordered vertically by their continuous risk *Z*-score to illustrate the smooth probability gradient across the cohort (gradient maps from 0% [Firebrick] to 100% survival [Dodger Blue]). **(B)** Waterfall heatmap of event endpoints (DFI and PFI) representing the increasing probability of recurrence or progression, *1 − S(t)*. The gradient is distinctly mapped to reflect the inverted physics of the endpoint (0% risk [Forest Green] to 100% risk [Firebrick]). **(C,D)** Temporal spaghetti plots mapping the individual probability trajectories for the Survival **(C)** and Event **(D)** endpoints over the 5-year clinical window. Trajectories are uniquely color-coded by the execution architecture utilized: patient records containing structurally intact multi-omic signatures that successfully navigated the *SuperLearner* (Path A) are plotted in Dark Cyan, whereas heavily fragmented patient records dynamically routed to the native *XGBoost* Path B are plotted in Red. The seamless overlap of these trajectories demonstrates a complete absence of mathematical outliers and visually validates that the *XGBoost* Path B preserves trajectory continuity, demonstrating stable algorithmic penetrance within the internal validation cohort.

To translate the validated continuous hazard Z-scores into actionable oncology metrics, the inference engine utilized geometric anchoring against the Phase III Breslow baseline hazards to extract individualized 1-year, 3-year, and 5-year clinical probabilities. To visualize the massive scale and stability of these 1,050 patient trajectories, we developed a 4-panel bifurcated composite model (Figure 11). To preserve mathematical clarity, the visualizations were strictly separated by endpoint physics. Probability estimates derived from *XGBoost* Path B routes should be interpreted with greater uncertainty than those from convergent *SuperLearner* synthesis (Path A), as Path B scores lack the Elastic Net penalization and cross-validated *λ* selection characteristic of the *MVL* meta-learner.

The waterfall heatmaps (Figures 11A,B) visually confirm the smooth gradient cascade of the individualized risk scores across the cohort. Specifically, Figure 11A illustrates survival endpoints (OS and DSS) as a decreasing probability of survival S(t), while Figure 11B illustrates event endpoints (DFI and PFI) as an increasing probability of recurrence or progression *1 − S(t)*.

More importantly, the temporal spaghetti plots (Figures 11C,D) map the individual probability trajectories based on the algorithm utilized: *SuperLearner* (Path A) or *XGBoost* (Path B). Figure 11C tracks the decreasing survival probabilities, whereas Figure 11D tracks the increasing event risks over the 5-year clinical landmark. These overlapping trajectories demonstrate a complete absence of mathematical outliers and visually confirm that the *XGBoost* Path B preserves trajectory continuity alongside the *SuperLearner*. This ensures that heavily fragmented patient records yield unified and stable predictions within the internal validation framework, demonstrating that the dual-track architecture maintains visual trajectory coherence for the deployment interface.

### 3.23 CancerRCDPredictor—a parameterized architecture for high-dimensional geometric navigation and AI diagnostic synthesis

To translate the complex outputs of the *SuperLearner* predictive framework into an accessible, interactive platform, we developed the CancerRCDPredictor application (available at URL: https://lbtgenomica.uenf.br/cancerrcdpredictor/). The execution of the multi-algorithmic machine learning pipeline across 96 independent multi-omic cohorts generated a data repository exceeding 80 GB, comprising high-resolution mathematical proofs, diagnostic curves, and patient-level trajectory matrices. To facilitate the exploration of this massive dataset, the web application was engineered as a translational interface, systematically organizing the *SuperLearner* outputs into distinct, user-driven modules.

The application follows a “Pedagogical First, Data Explorer Second” structural design. To aid in the interpretation of high-dimensional non-proportional hazard functions and *SHAP* topologies, the platform anchors each capability module with an educational ex*a*mpl*e*. Users are provided with static, manuscript-grade visualizations accompanied by executive summaries that explain the interpretation of multi-omic risk trajectories and functional interactions (e.g., lethal synergy versus protective antagonism).

Beyond the educational examples, the platform provides dynamic 96-Cohort Data Explorers. These parameterized engines allow users to selectively query the 59 GB repository by inputting specific cancer lineages and terminal clinical endpoints (e.g., Overall Survival, Disease-Specific Survival). The application is divided into four primary analytical modules, each serving a distinct scientific and clinical utility:

Global Impact (*SHAP* Beeswarms): This module visualizes the population-level influence of retained multi-omic signatures. It allows users to assess how the continuous expression or discrete presence of specific genomic elements drives non-proportional hazard projections across an entire cohort.
Interaction Topologies (Dependence): This module isolates the geometric relationships between interacting omic features. It provides high-resolution spatial mapping of synergistic, antagonistic, and bifurcating feature pairs, detailing how combined multi-omic states enhance or suppress survival risk.
*MVL* Performance (Time-Dependent AUROC): This module evaluates the temporal stability of the *SuperLearner* framework. It outputs dual-panel ROC curves, tracking the strict discriminatory capability (AUC) of the models at 1-year, 3-year, and 5-year clinical horizons to validate prognostic resilience over time.
Precision Oncology (Patient Trajectories) and AI Diagnostic Synthesis: Transcending standard individualized risk stratification, this module utilizes *SHAP* waterfall plots to deconstruct the predictive logic for individual patients, mapping how specific molecular alterations steer a patient’s trajectory toward lethal or protective outcomes relative to the population baseline. To fully operationalize this geometric data, the module features an integrated Large Language Model (LLM) acting as a hypothesis-generating molecular interpretation assistant (Digital Molecular Tumor Board, Supplementary Note 12). This AI diagnostic engine autonomously extracts the Top five patient-specific *SHAP* topological coordinates, decoding the alphanumeric signatures back to their precise biological origins. The module meticulously cross-references each localized molecular driver against literature-verified RCD pathways, specific multi-omic layers (e.g., CpG methylation vs. mRNA transcripts), and population-level tumor phenotypes. By dynamically synthesizing these multi-dimensional layers, the AI engine generates an audit-ready, continuous clinical narrative. Crucially, the diagnostic module mathematically contextualizes contradictory phenotypic correlations as substantial multi-omic tumor heterogeneity rather than algorithmic noise, bridging the gap between high-dimensional machine learning and actionable clinical intelligence. Finally, an interactive clinical dialogue interface allows users to directly query the AI synthesis to explore tailored therapeutic vulnerabilities.

By compartmentalizing the data into these specialized modules, the architecture ensures that the geometric fidelity and algorithmic outputs of the *SuperLearner* framework can be systematically explored and utilized for both broad cohort analysis and individualized clinical evaluation.

## 4 Discussion

The pervasive reliance on strictly parametric survival frameworks in precision oncology (e.g., standard Cox PH regression and penalized linear formulations) often fails to capture the high-dimensional, non-linear realities of multi-omic biological spaces. Furthermore, traditional approaches to multi-omic “integration” frequently mistake integration for modeling distinct omic layers separately and then attempting to merge their fragmented results *post-hoc*. In this study, we operationalized true integrative multi-omics: the simultaneous, native estimation of predictive values across all seven molecular layers within a single, unified topological matrix. By physically isolating predictive models to localized cancer execution domains, our architecture prevented cross-cohort contamination and safeguarded the clinical trajectory from the geometric instability common in generic pan-cancer amalgamations.

The strict necessity of this approach was geometrically confirmed by our Phase II CANARY diagnostic. After evaluating the initial library of 120 algorithmic configurations and disqualifying 24 structurally compromised matrices, testing the remaining 96 advanced cohorts under classical *CoxNet* constraints resulted in exactly zero strata surviving the proportional hazards *μ*-ladder. This collective structural failure across pan-cancer strata empirically proves that the standard reliance on linear, proportional hazard assumptions in multidimensional omics is mathematically and biologically invalid; the native survival topology is fundamentally non-proportional. Importantly, this finding does not imply the elimination of Cox-based structures entirely: the *MVL* meta-learner employs Cox-norm penalization for shrinkage, Breslow estimation converts hazard scores to clinical probabilities, and *XGBoost* uses Cox partial-likelihood as its survival loss function. The framework thus combines non-linear learners with Cox-based calibration and meta-modeling, rather than bypassing proportional hazards entirely. This systematic CANARY failure across all tested strata serves as the primary linear PH benchmark comparison for the non-linear *SuperLearner* architecture reported in Phase III.

Crucially, the deployment of the Phase III Quadripartite ML Ensemble demonstrates the inherent advantage of Multi-View Meta-Learning ensemble architectures over isolated model reliance (van der Laan et al., 2007). Rather than claiming generalized algorithmic superiority, the *SuperLearner* functions explicitly as a robust anti-overfitting barrier. This topological stability was anchored prior to overarching integration by enforcing a rigid 35% geometric missingness boundary, surgically excising computationally excluded variables (vectors presenting with fundamentally hollow predictor domains) to ensure that all downstream model convergence occurred strictly upon genuine biological mass. This directly intercepts the severe geometric instabilities and calibration failures endemic to high-dimensional “pan-cancer” modeling, vulnerabilities that have been rigorously documented in recent large-scale benchmarking frameworks [e.g., the SurvBoard consortium; (Wissel et al., 2025)]. Tree-based paradigms, such as Sparsity-Aware XGBoost, while excelling at mapping high-dimensional spaces, routinely suffer from extreme variance inflation when pushed to biological structural limits. By deploying an *MVL* Elastic Net to systematically synthesize the divergent inductive biases of the four foundational architectures, *RSF*, Sparsity-Aware *XGBoost*, insulated Survival-*Boruta* topological parameters, and *MTLR*, the architecture naturally penalizes isolated mathematical outliers.

This multi-view integration mitigates the severe effects of multi-omic collinearity, compressing the overarching hazard predictions into a highly stabilized, cross-validated consensus space that successfully prevents the temporal model decay inherent to proportional hazards frameworks, seamlessly maintaining robust resolution across 1-, 3-, and 5-year clinically truncated progression horizons. Furthermore, the *SuperLearner* was engineered to execute a robust “Topological Floor Defense.” Operating as an architectural “No One Stays Behind” policy, the Elastic Net synthesis autonomously rescued highly fragmented, chaotic biological spaces, such as the lush LGG multi-omic topography, by distributing democratic trust across the base learners. This mathematically protected deeply volatile matrices from crashing into stochastic noise, rigidly anchoring the pan-cancer survival floor at a globally predictive 0.526 baseline regardless of individual algorithmic failure.

This strict simultaneous integration actively enforced a competitive evaluation between distinct cancer omic networks, exposing a substantial mathematical phenomenon: the systematic, algorithmic displacement of somatic mutations and CNVs by downstream dense continuous multi-omic features. This massive displacement aligns precisely with established machine learning observations regarding ‘cardinality bias’ within decision tree logic (Strobl et al., 2007). Tree-based learning arrays inherently maximize predictive entropy. A binary mutation (present/absent) offers only a single, blunt bifurcation point, lacking the mathematical flexibility required to track a patient’s longitudinal, continuously compounding survival hazard over multi-year clinical timelines. Conversely, continuous molecular profiles, such as mRNA abundance, transcript isoform gradients, and epigenetic methylation, natively provide thousands of micro-partitions. Specifically, binary variables frequently suffered from localized intra-cohort variance starvation; lacking sufficient numerical deviation within strictly bound execution strata, they yielded negative Information Gain and were mechanically ejected by the gradient boosting paradigms. Consequently, because they offer the algorithmic trees an exponentially denser spectrum of continuous hazard gradients, active continuous multi-omic arrays mathematically displaced static baseline genotypes during overarching multi-omic competition. This algorithmic displacement rigorously mirrors recent multi-omic predictive evaluations, which consistently demonstrate that continuous multi-omic strata map the functional tumor state so comprehensively that enforcing the integration of sparse, static mutations routinely yields mathematically negligible downstream prognostic gains (Herrmann et al., 2021). While exactly 142 mutations and 196 CNVs successfully established baseline viability within the 12,613 prognostic pool, when forced through the strict apex limits of the Quadripartite gauntlet, zero genotypic markers remained among the ultimate 150 pan-cancer golden anchor concordances. These 150 anchors do not represent absolute biological superiority; rather, they identify the precise subset of features possessing a continuous topological geometry robust enough to ensure systematic retention across all four highly distinct machine learning architectures simultaneously.

Crucially, this overarching mathematical suppression must not be conflated with biological inertness. Our deliberate deployment of the Sparsity Isolation framework explicitly tested discrete binomial variables separated entirely from continuous phenotypic matrices. This framework definitively proved that baseline somatic mutations and CNVs possess highly significant independent prognostic trajectories within their isolated architectures. Their subsequent suppression in the fully integrated Terminal Harvester structure functionally confirms that highly dense, continuously active RNA phenotypes effectively “mask” sparse binary signals in joint predictive arrays. However, this suppression is not absolute. As definitively documented via the topological “Sparsity Rescue Effect,” when severe cohort-specific data missingness causes these dominant localized transcriptomic cascades to structurally collapse, the *SuperLearner* autonomously pivots. It mechanically falls back upon the underlying somatic distributions to rescue the survival bounds, confirming that discrete genotypic signatures fundamentally function as essential architectural fail-safes when the primary continuous phenotype is compromised.

Ultimately, computational predictive accuracy remains clinically inert without actionable, transparent biological interpretability. Traditional biomarker analyses routinely extract a unified “top 10” signature and universally apply it, falsely assuming that a generalized cohort-level ranking accurately defines every distinct patient’s risk. Our integration of *post-hoc* exact *TreeSHAP* and *LIME* architectures explicitly overturns this generalization (Lundberg et al., 2020). While sparse surrogate models (*LIME*) were deployed to map restricted individual boundaries, their routinely suppressed Explanation Fits (*R*^2^ < 0.10) provided explicit mathematical proof that single-patient survival boundaries remain too highly non-linear for any linear proxy to fully navigate. Consequently, while the *SuperLearner* defined overarching global hierarchies, extracting algorithmically localized (N × N) interaction tensors and patient-specific waterfall trajectories from the exact *TreeSHAP* architecture demonstrated that individual patient survival is fundamentally idiosyncratic. This individualized focus directly responds to the urgent mandate in contemporary precision oncology to abandon generalized algorithmic opacity in favor of direct, patient-specific feature attribution frameworks that ensure actionable clinical translation (Klauschen et al., 2024). We documented that what severely accelerates a lethal trajectory in one patient is frequently dictated by a highly distinct, personalized set of multi-omic features (e.g., specific mRNA overexpression synergizing with epigenetic layers) that may be entirely dormant or irrelevant in another patient harboring an equal mortality risk. Furthermore, this localized tracking dynamically diagnoses whether mortality is driven by an isolated, targetable biological anomaly (an apex driver) or dictated by diffuse polygenic degradation, where hundreds of minor multi-omic aberrations synergize into a massive cumulative hazard penalty. Most significantly, dissecting these multi-dimensional *TreeSHAP* topographies exposed the mechanical phenomenon of “Antagonistic Rescue” (Figures 7, 8B). While classical models assume survivability is dictated by simply summing isolated risk biomarkers, the Quadripartite architecture proved that the localized presence of an apex lethal mRNA signature can be mathematically and biologically neutralized if the patient concurrently possesses an intersecting, protective transcript isoform. This ability to natively resolve cross-layer rescue capabilities signifies the structural invalidation of linear additivity; it confirms that true precision oncology forecasting hinges entirely on synergistic and antagonistic multi-omic interactions. By mathematically singularizing the importance of variables at the strict patient level and revealing exactly how varying omic permutations combine to drive lethal or protective deviations from the baseline, our architecture successfully shifts predictive modeling from a generalized cohort stratification tool into a probabilistic, high-resolution framework for individualized multi-omic survival topology mapping, with clinical interception remaining contingent upon multi-institutional external validation.

External validation of the *MVL SuperLearner* on the CPTAC cohort yielded mixed results that warrant careful interpretation. KIRC achieved statistically significant cross-cohort generalizability (C-index 0.675, *p* = 0.017), demonstrating that the multi-omic predictive architecture can transfer to an independent, prospectively collected cohort. The preservation of peak temporal discrimination at 3 years (AUC 0.722) in both TCGA and CPTAC further supports a genuine prognostic signal in this cancer type. LUSC showed modest discrimination (*p* = 0.114) with reasonable calibration (Brier 1 yr. = 0.051, calibration slope 0.59), below the significance threshold.

However, only KIRC achieved statistical significance (*p* = 0.017) among the six cancers with adequate event counts (≥20 events). HNSC and LUAD approached but did not reach the *p* < 0.05 threshold (*p* = 0.077 each). The remaining three adequately powered cancers (GBM, LUSC, PAAD) showed no evidence of cross-cohort discriminative signal above chance. The negative cross-cohort Spearman rank correlation (*ρ* = −0.250) indicates that cancer types ranked as “easy” or “difficult” in TCGA do not maintain this ordering in CPTAC. Three non-exclusive factors likely contribute to this inversion. First, the dimensionality gap is substantial: CPTAC retains only 44–77% of TCGA’s omic predictors per cancer type (Supplementary Table S24), and the models encounter structurally missing features that degrade predictive resolution. Second, CPTAC represents prospectively collected proteogenomic cohorts with different biospecimen processing, platform technologies, and clinical annotation standards than the retrospective TCGA collection, reflecting true biological and technical batch effects that any multi-omic predictor must address. Third, the TCGA derivation cohort, while internally validated via bootstrap and permutation, was developed on a single data resource; the CPTAC results suggest that at least some portion of the TCGA C-index distribution reflects dataset-specific patterns rather than universally generalizable biological signal*s*.

The systematic over-prediction of risk in CPTAC (all O/E ratios < 1.0) reflects the expected consequence of anchoring CPTAC risk scores against TCGA Breslow baseline hazards, a methodological necessity to preserve strict blinding, but one that introduces population-level calibration drift when event rates differ between cohorts. Recalibration of baseline hazards on the validation cohort would require access to validation outcomes, which is appropriate in a deployment setting but was not the objective of this blinded external validation exercise.

These findings should not be interpreted as a failure of the predictive framework but as a realistic demonstration of the challenges inherent in multi-omic external validation. The CPTAC cohort remains the best available approximation of the multi-cancer, multi-omic landscape required for pan-cancer validation, and its limitations (reduced cancer type diversity, OS-only endpoint availability, and lower omic layer coverage) are characteristics of current public data resources, not of the predictive architecture. The KIRC result demonstrates that transfer is possible; the mixed results for other cancer types underscore the necessity of larger, more diverse multi-omic validation cohorts for comprehensive assessment of pan-cancer survival predictors.

The present findings align with a growing body of evidence supporting multi-omics integration, non-parametric survival modeling, and SHAP-based interpretability in oncology, while also reflecting the persistent challenges of external validation that characterize this field. Prior pan-cancer studies have demonstrated that multi-omics integration improves prognostic accuracy over single-modality approaches, reporting an average C-index improvement of 6.5% across 15 TCGA cancer types using deep learning (Chai et al., 2021), while interpretable meta-learning architectures have resolved pathway-level prognostic signals across multiple cancers (Cho et al., 2023). The failure of proportional hazards assumptions we documented through the CANARY diagnostic is consistent with comparative analyses showing that Cox PH underperforms *RSF* and other tree-based methods in complex cancer survival settings (Buyrukoglu, 2024; Takele and Chen, 2026). Our use of *TreeSHAP* to identify multi-omic interaction topologies builds on work demonstrating that *SHAP* interaction values recover known gene-clinical interactions in prostate cancer and identif*y* novel effects in high-grade disease (Li et al., 2020), and on a systematic review confirming *SHAP* as the dominant explanation method for tree-based oncology models (Ghasemi et al., 2024). However, several single-cancer RCD models have reported higher C-index values, including gastric cancer signatures reaching values above 0.80 (Bai et al., 2024; Sun et al., 2024) and breast cancer recurrence models at 0.837 (Noman et al., 2025). These results suggest that the contribution of the present framework is not strict discriminative superiority, but rather its pan-cancer scope, its zero-leakage harmonization design, and its systematic demonstration that linear interpretative surrogates fail across multi-omic topologies (*LIME R*^2^ < 0.10). As noted in the gastric cancer literature, computational RCD models remain limited without experimental validation (Sun et al., 2024), a limitation that applies here as well. The modest CPTAC external validation results (KIRC significant, median C-index 0.582) reflect the broader challenge of multi-omic transportability: even landmark multi-omics studies achieving external validation across independent cohorts, such as liver cancer (Chaudhary et al., 2018) or nasopharyngeal cancer (Alabi et al., 2023), typically operate within single cancer types and narrower modality scopes.

However, the ultimate realization of this precision oncology framework requires rigorous context regarding its clinical generalizability. While our partitioned, blinded internal validation strategy successfully insulated the predictive architecture from information leakage and maintained strict 7-layer multi-omic harmony, ensuring the models were evaluated on genuine biological accuracy rather than their tolerance for disparate external preprocessing techniques, this approach inherently lacks multi-institutional external testing. In precision oncology, external validation remains the gold standard for demonstrating true cross-demographic generalizability. Given the current global scarcity of fully independent, comprehensive multi-omic public datasets spanning 33 distinct tumor types, our internal blinded approach serves as a mathematically robust and highly controlled diagnostic intermediate. Future large-scale, multi-institutional sequencing efforts will be essential to definitively establish the universal clinical portability of these personalized multi-omic trajectories.

The clinical integration of the CancerRCDPredictor platform provides several critical advantages for precision oncology, overcoming the cognitive and interpretive barriers commonly associated with high-dimensional machine learning. Primarily, the application achieves automated multi-omic translation by seamlessly bridging the gap between complex mathematical algorithms and clinical biology, converting raw *SHAP* topologies into readable, coherent patient diagnostic narratives. This functionality operationalizes Explainable AI (XAI) in practice; rather than providing an opaque ‘black-box’ survival score, the platform empowers researchers and oncologists by explaining precisely why a prediction was made, isolating the exact genetic, transcriptomic, or epigenetic drivers responsible for altering patient risk.

Furthermore, the architecture is specifically capable of contextualizing tumor heterogeneity through dynamic tumor state reasoning. By reconciling seemingly contradictory statistical data, such as a gene exhibiting lethal effects via transcript expression but protective effects via methylation, the platform helps clinicians understand adaptive tumor plasticity, stemness transitions, and multi-pathway resistance mechanisms. Ultimately, this structural design ensures guided clinical actionability while maintaining its identity as a translational research instrument. Operating under a rigorous Domain Boundary Governance framework, the integrated Digital Molecular Tumor Board enforces a clear separation between molecular hypothesis generation and generalized clinical treatment guidance. By highlighting a patient*’*s dominant RCD pathways and actionable metabolic or stromal vulnerabilities, the system avoids prescriptive medical recommendations and instead generates tailored follow-up queries, providing oncologists with immediate, biologically grounded starting points for targeted therapy discussions and independent experimental validation.

Important limitations regarding clinical translation must be acknowledged. First, the CancerRCDPredictor platform, including its LLM-based Digital Molecular Tumor Board module, has not undergone prospective clinical validation, formal clinician evaluation, or head-to-head comparison against established clinical prognostic tools. The term “Digital Molecular Tumor Board” describes the module’s hypothesis-generation functionality and does not imply readiness for clinical deployment. Second, the reported predictive performance derives from retrospective TCGA data and, despite internal blind validation (N = 1,050) and bootstrap uncertainty quantification, clinical utility requires future multi-institutional external validation. Third, while the platform generates biologically grounded hypotheses for targeted therapy discussions, it does not provide prescriptive treatment recommendations and is intended exclusively as a translational research instrument.

## Data Availability

The complete dataset and codebase are publicly hosted and fully accessible for all scripts, feature lists, hyperparameters, seeds, and environment files at the following GitHub repository (https://github.com/BioCancerInformatics/CancerRCDPredictor/) and can be explored and downloaded from the CancerRCDPredictor shiny application (https://lbtgenomica.uenf.br/cancerrcdpredictor/). The reproducibility package within the repository explicitly contains: 48 sequentially numbered core R scripts (54 total including auxiliary scripts) organized in four phase folders (Phase I through Phase IV) with execution-order README documentation; sessionInfo outputs for both the ZIMA computing server (R 4.5.1, NixOS) and the local workstation (R 4.2.3, Windows 10); renv.lock files enabling exact R environment replication via renv::restore(); random-number generator seed tables (random_seeds.txt and random_seeds_NB.txt) documenting all *set.seed()* declarations across Phases I–IV and all supplementary analyses; a per-stratum hyperparameter table (*hyperparameters.tsv*, 1,728 entries covering XGBoost, RSF, MTLR, Boruta, and MVL hyperparameters across 96 modelable strata); locked per-stratum feature lists (*feature_lists.tsv*) extracted from the Phase III model bundles; the CPTAC external validation predictor script with preflight verification; and the Schoenfeld residual audit pipeline with preflight verification and the Schoenfeld_120_Strata.tsv output table. The serialized Phase III model bundles (96 strata, 4 models per stratum, exceeding 80 GB) are available upon reasonable request to the corresponding author due to storage constraints.

## Footnotes

1
https://cancerrcdshiny.shinyapps.io/cancerrcdshiny/
